# Clustered Distribution of Natural Product Leads of Drugs in the Chemical Space as Influenced by the Privileged Target-Sites

**DOI:** 10.1038/srep09325

**Published:** 2015-03-20

**Authors:** Lin Tao, Feng Zhu, Chu Qin, Cheng Zhang, Shangying Chen, Peng Zhang, Cunlong Zhang, Chunyan Tan, Chunmei Gao, Zhe Chen, Yuyang Jiang, Yu Zong Chen

**Affiliations:** 1 Department of Pharmacology and Pharmaceutical Sciences, School of Medicine, Tsinghua University, the Ministry-Province Jointly Constructed Base for State Key Lab-Shenzhen Key Laboratory of Chemical Biology, the Graduate School at Shenzhen, Tsinghua University, Shenzhen, and Shenzhen Technology and Engineering Laboratory for Personalized Cancer Diagnostics and Therapeutics, PO Box 518000, P. R. China; 2 Bioinformatics and Drug Design Group, Department of Pharmacy, and Center for Computational Science and Engineering, National University of Singapore, Singapore 117543; 3 NUS Graduate School for Integrative Sciences and Engineering, Singapore 117456; 4 Innovative Drug Research Centre and College of Chemistry and Chemical Engineering, Chongqing University, Chongqing, P. R. China; 5 Zhejiang Key Laboratory of Gastro-intestinal Pathophysiology, Zhejiang Hospital of Traditional Chinese Medicine, Zhejiang Chinese Medical University, Hangzhou, P. R. China

## Abstract

Some natural product leads of drugs (NPLDs) have been found to congregate in the chemical space. The extent, detailed patterns, and mechanisms of this congregation phenomenon have not been fully investigated and their usefulness for NPLD discovery needs to be more extensively tested. In this work, we generated and evaluated the distribution patterns of 442 NPLDs of 749 pre-2013 approved and 263 clinical trial small molecule drugs in the chemical space represented by the molecular scaffold and fingerprint trees of 137,836 non-redundant natural products. In the molecular scaffold trees, 62.7% approved and 37.4% clinical trial NPLDs congregate in 62 drug-productive scaffolds/scaffold-branches. In the molecular fingerprint tree, 82.5% approved and 63.0% clinical trial NPLDs are clustered in 60 drug-productive clusters (DCs) partly due to their preferential binding to 45 privileged target-site classes. The distribution patterns of the NPLDs are distinguished from those of the bioactive natural products. 11.7% of the NPLDs in these DCs have remote-similarity relationship with the nearest NPLD in their own DC. The majority of the new NPLDs emerge from preexisting DCs. The usefulness of the derived knowledge for NPLD discovery was demonstrated by the recognition of the new NPLDs of 2013–2014 approved drugs.

The focus of drug discovery has moved from natural products (NPs) to technology-derived synthetic molecules for about 20 years^1^ without the anticipated drug productivity improvement^2^. Although being largely sidelined, NPs are still relevant^3^,^4^ with NP-related small molecule drugs representing 29.5% of the 132 FDA approved drugs in 2008–2012 (Supplementary Table S1), NP-related drugs include NPs and NP semi-synthetic derivatives, mimetics, and pharmacophore-guided synthetic molecules^3^. The NPs from which the NP-related drugs have been derived are named as the NP leads of drugs (NPLDs). There is a renewed interest in discovering drugs^5^ from NP privileged structures^6^ and derivative libraries^7^. The knowledge of the distribution of the NPLDs in the chemical space provides useful clues for prioritizing the relevant efforts.

Although NPs are in well-defined subspaces of the chemical space^8^, because of their enormous number^9^, structural diversity^9^,^10^ and molecular complexity^11^, only a fraction of NPs can be practically explored in the foreseeable future. Drug discovery efforts need to be prioritized towards the NPs with higher discovery potentials. The key questions are which NPs to explore and where to find them. Evidences suggest that some NPLDs may congregate in specific drug-productive regions of the chemical space. Certain NP chemical classes (e.g. steroids and nucleosides) are drug prolific^12^. Half of the drugs are made of dozens of molecular frameworks^13^. For many drugs, their individual molecular, physicochemical and topological pharmacophore properties^14^,^15^,^16^ and the corresponding principal components^17^,^18^,^19^,^20^ are constrained in specific ranges. Moreover, the GPCR, kinase and protease targeting agents have been reported to each cluster together in the chemical space^1^. These studies have consistently shown that a substantial percentage of the NPLDs congregate in the chemical space. However, the extent and the detailed distribution patterns of the congregation of NPLDs in the chemical space and the mechanisms leading to such patterns have not been fully investigated.

There is a need to study these questions from different structural and molecular binding perspectives to gain a deeper understanding of the structural characteristics of NPLDs and to find clues for guiding the search of new NPLDs. In this work, we determined the distribution patterns of the 348 and 94 NPLDs of 749 pre-2013 approved and 263 clinical trial small molecule drugs (Supplementary Tables S2, S3) in the chemical space represented by the molecular scaffold trees^21^ and the molecular fingerprint based hierarchical clustering tree^22^,^23^ of 137,836 non-redundant NPs^3^,^24^,^25^. The number of NPs profiled here is comparable to those of the earlier large-scale NP studies^9^,^21^,^26^. Molecular fingerprints were used for representing NPs in the hierarchical clustering tree because of its demonstrated effectiveness in structural similarity searching, and its extensive applications in drug lead discovery^12^,^23^,^27^,^28^,^29^,^30^.

The derived distribution patterns were studied from the perspective of preferential binding of NPLDs to the privileged target-sites for determining whether it contributes to the formation of these patterns. We also evaluated whether these patterns are distinguished from those of the bioactive NPs and how they evolve with time. We further tested whether the derived knowledge can be explored for NPLD discovery by applying it to retrospectively judge the development potential of the new NPLDs of 2013–2014 approved drugs uninvolved in the derivation of the NPLD distribution patterns. New technologies are expected to significantly expand the currently accessible NP chemical space^31^,^32^ and their potential impact is not reflected in this study.

Drug scaffolds have been well analyzed^13^ and drug distribution in the chemical space have been extensively studied from the perspectives of specific molecular and physicochemical properties^1^,^14^,^15^,^16^,^17^,^18^,^19^,^20^. To the best of our knowledge, our work is the first large-scale and systematic study of the detailed distribution patterns of the largest set of NPLDs in the chemical space from the perspectives of their molecular scaffolds and structures. The molecular scaffold analysis was intended for determining whether there is a significant change in the congregation patterns of the NPLDs in comparison to the previous studies^13^,^33^. The molecular structural analysis was intended for further probing the complex structural features of the NPLD congregation phenomenon and the underlying molecular mechanisms that might contribute to the clustering of NPLDs with particular focus on the possible influence of the binding of NPLDs and their derivatives to the privileged target sites.

## Methods

We collected 442 NPLDs^1^,^3^,^27^,^28^,^34^,^35^ and the information about their NP origin^1^,^28^ from the literature. We also collected 169,037 NPs from the ZINC^24^, TCM-ID^29^, TCM@Taiwan^25^, and other literatures^3^. For database entries with multiple non-linked components, only the largest component was selected. Hydrogens were added and small fragments (counter ions, solvent molecules, etc.) were removed by using Corina, The number of NPs were reduced to 137,836 after removing the duplicate entries, small NPs with molecular weight <50 Daltons (drug leads are >100 Daltons^30^) and the NPs whose molecular fingerprints could not be computed by using available software tools such as PaDEL^36^. Duplicates were identified and removed by structural comparison based on a set of 98 molecular descriptors we have used for classifying bioactive molecules^37^ and implemented in the online server MODEL^38^, open-source software PaDEL^36^, and our own software, which can distinguish different molecules non-distinguishable by the 881-bit Pubchem molecular fingerprints.

In deriving the molecular scaffold trees of the 442 NPLDs and 137,836 NPs, Scaffold Hunter v2.3.0^21^ was used to select the NPLDs and NPs with ring structures and to subsequently cluster them into molecular scaffold trees by using default rule set in the Scaffold Tree Generation window. The molecular fingerprint based hierarchical clustering tree of the 442 NPLDs and 137,836 NPs was generated by using the Matlab statistics toolbox with the structures of the NPs represented by 2D molecular fingerprints^23^ (specifically, the 881-bit PubChem substructure fingerprints computed by using PaDEL^36^) and with their similarity levels measured by the Tanimoto coefficient *Tc*^22^,^23^ and the complete linkage. *Tc* was used because it is the most popular similarity metric for molecular fingerprint based measurement of compound similarity^23^. Complete linkage was used because of its relatively good performance in clustering bioactive compounds in a recent comparative study^39^. The hierarchical tree graphs were generated by using EMBL automatic tree generator in iTOL version-1.8.1^40^ with the distance of the NPs measured by the Tanimoto distance *Td* = 1-*Tc*. In analyzing the physicochemical landscapes of the NPs in specific regions of the chemical space, we used MODEL^38^ and Discovery studio 3.1.1 software to compute eight molecular descriptors frequently used for analyzing drug-like^14^,^41^,^42^,^43^ and lead-like^43^,^44^ features. These are molecular weight (MW), lipophilicity AlogP and logD, polarizability (PZ), and the number of O+N (ON), hydrogen bond donor (HD), hydrogen bond acceptor (HA), rotatable bond (RB), and rings (RI).

To determine whether the clustering of the NPLDs in specific sub-regions of the chemical space are statistically more significant than chance, our derived distribution patterns of the NPLDs with respect to those of the randomly shuffled NP communities were analyzed by the method used for determining the statistical significance of the phylogenetic clustering of traditional medicinal plants^45^. In this method, the mean Tanimoto distance *MTd* of the NPLDs in every NPLD-clustered sub-region was compared to the *MTd* values of these NPLDs in 60,000 randomly generated NP sub-regions. By using the algorithms implemented in the Phylocom: software^46^, a one-tailed P-value and a net relatedness index (*NRI*) were calculated for each sub-region. The P-value is the number of randomly selected NPs that are more clustered than the NPLDs in each sub-region divided by the number of runs (60,000 in this study). The desired significance level α of the P-value was further adjusted by Bonferroni correction to α′ = α/N (N is the number of independent statistical significance tests, which is 60 in this study)^47^. The *NRI* is a standardized effect size measure of the community structure in each sub-regions, which is the difference in average *Td* between the NPLDs and the 60,000 randomly generated NP sub-regions, and standardized by the standard deviation of the *Td* values in 60,000 randomly shuffled sets of NP communities. The sign of *NRI* informs whether the NPLDs are more clustered (*NRI* > 0) or more dispersed (*NRI* < 0) than the NPs in each sub-region. These quantities were calculated by using Phylocom v4.1^46^ with the *Td* values of the NPLDs and NPs as input data.

## Results and Discussion

### Distribution profiles of NPLDs in the chemical space from the perspectives of molecular scaffolds and molecular structures

**Figure 1:**
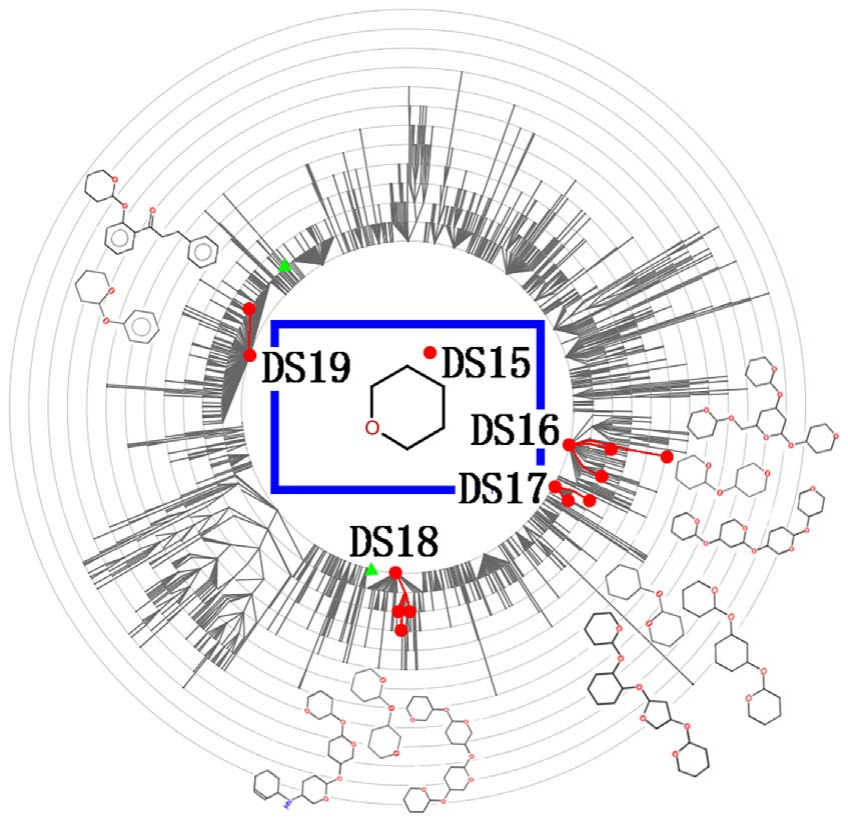
Distribution of the natural product leads of approved and clinical trial drugs in branch 5 of the Scaffold-Hunter derived molecular scaffold trees of the 134,097 natural products and 411 natural product leads. The drug-productive scaffolds or scaffold parent-child sub-branches (DSs) are indicated by red dots or red dots connected by red lines, which marked by the respective label DS15-DS19. The green triangles indicate the natural product leads outside the DSs. Some of the representative scaffolds in these DSs are shown in the Figure. The more complete sets of the representative scaffolds are shown in the Supplementary Figure S2.

There are 411 NPLDs and 134,097 NPs with ring structures. These were grouped by Scaffold hunter^21^ into molecular scaffold trees of 39,051 scaffolds (114 are drug-productive). The distribution of the NPLD scaffolds in this large-scale analysis is similar to the previous findings^13^,^33^, the majority (62.7%) of the NPLDs of the approved drugs and a substantial percentage (37.4%) of the NPLDs of the clinical trial drugs congregate in 62 drug-productive scaffolds or scaffold parent-child sub-branches (DSs) labeled as DS1 to DS62 (Figure 1, Supplementary Table S4 and Figures S1–S5). A DS is defined as a scaffold with ≥2 NPLDs that have yielded ≥1 approved drug or a scaffold parent-child sub-branch with ≥2 NPLD-producing scaffolds that have yielded ≥1 approved drug. These DSs have collectively yielded 69.6% approved and 44.4% clinical trial drugs. The congregation of NPLDs in the DSs coupled with the earlier finding that the GPCR, kinase and protease targeting agents each are clustered together in the chemical space^1^ indicates that NPLDs of the same and different scaffolds against the same classes of targets may on a broader scale be clustered together in the chemical space. To facilitate the visualization of our generated scaffold trees by using Scaffold Hunter, the resulting scaffold database was exported as a SQL file that can be downloaded at http://bidd.nus.edu.sg/group/NPLD_Distribution/NP_ScaffoldHunter.zip.

**Figure 2:**
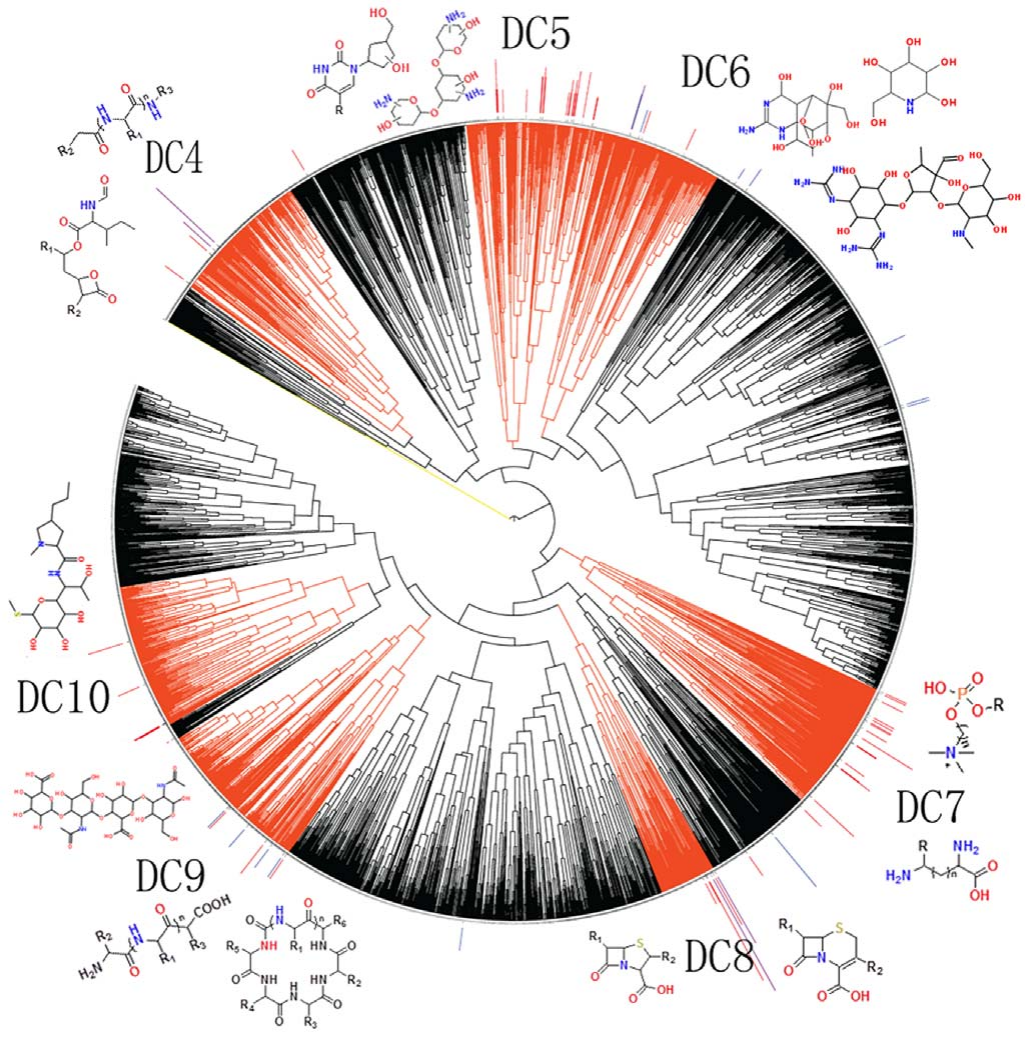
Distribution of the natural product leads of approved and clinical trial drugs in branch 3 of the substructure-fingerprint clustering tree of the 137,836 natural products and 442 natural product leads. The drug-lead productive clusters are red-orange colored and marked by the respective cluster label DC4-DC10. The red, purple and blue lines on top of the clustering tree indicate the locations of the approved, approved + clinical trial, and clinical trial drug-leads with the height correlating with the number of approved + clinical trial drugs.

To probe the larger-scale distribution patterns of NPLDs in the chemical space from the perspective of molecular structures, we generated a molecular fingerprint based hierarchical clustering tree of the 442 NPLDs and 137,836 NPs. The derived tree is composed of 33 main branches (Supplementary Figure S6 and Table S5). Most (87.9%) branches are drug-productive, reflecting the fact that NPs primarily co-evolve and interact with proteins^6^ and a variety of chemical classes^3^,^4^ and target families^48^,^49^ have been therapeutically explored. Nonetheless, NPLDs within each branch are mostly clustered together, with 341 (77.2%) NPLDs (82.5% approved, 3.0% clinical trial) clustered in 60 drug-lead productive clusters (DCs) labeled as DC1 to DC60 (Figure 2, Supplementary S7–S10 and Table S6). A DC is defined as a relatively small region of the molecular fingerprint characterized chemical space with moderate to high concentration of NPLDs yielding ≥1 approved drug.

To facilitate the analysis of the clustered distribution of the NPLDs, we generated the heat map of the proximity matrix of 442 NPLDs against 137,836 non-redundant NPs. The proximity matrix was calculated by using molecular fingerprint Tanimoto distance *Td* between NPLDs and NPs with the row and column positions representing the NPLDs and NPs in the same order as their respective positions in the hierarchical clustering tree of the NPLDs and NPs. The heat map was created by using the heatmap.2 function of the gplots package in R with the red to yellow colors indicating the stronger to weaker structural similarity between the NPLDs and NPs. The heat map for branch 4 and 9 are shown in Supplementary Figures S11–S12 and those of the other branches can be downloaded from http://bidd.nus.edu.sg/group/NPLD_Distribution/NP_heatmaps.zip.

We found that 11.7% of the NPLDs in the DCs have remote-similarity relationships (0.57 ≤ *Tc* < 0.7) with the nearest NPLD in their own DC, and another 24.9% of the NPLDs in these DCs have intermediate-similarity relationship (0.7 ≤ *Tc* < 0.85) with their nearest NPLD in their own DC. Remote-similarity relationships have been reported in compounds with cross-pharmacology relationships^50^ and between a bioactive compound and its scaffold hopping parent bioactive compound^51^. Therefore, the DCs broadly cover the high-similarity to remote-similarity relationships for capturing similar activities, and cross-pharmacology and scaffold hopping types of relationships.

These DCs have collectively yielded 87.9% approved and 68.8% clinical trial drugs. In particular, 56.0% approved and 67.4% clinical trial NPLDs are clustered in 22 NPLD-prolific DCs (Table 1) that have collectively yielded 68.4% approved and 39.2% clinical trial drugs, which is consistent with the report that half of the drugs are made of dozens of molecular frameworks^13^. The NPLD-prolific DCs were ranked based on the ratio of the approved NPLDs to the NPs in each DC. Partly because of the inadequate exploration and partly because of the limited availability of the relevant information, these ratios may not fully reflect the reality but nonetheless provide useful indications. We found that 60% of the top-10 NPLD-prolific DCs with >100 searchable NPs in Table 1 are among top-ranked DCs with higher approved NPLD to NP ratios. Thus, drug productivity of these top-ranked DCs seems to arise from higher NPLD yields instead of the higher number of NPs explored. The top-ranked DC38 and DC8 in Table 1 were excluded because they have <100 searchable NPs. If counted, they are among the DCs with highest approved NPLD to NP ratios.

### Statistical significance of the clustering of NPLDs in the DCs

The statistical significance of the clustering of the NPLDs in every DC was evaluated by using the Phylocom software^46^ to calculate the P-value and *NRI* of the NPLDs against the chance clustering of the NPs in the DC from 60,000 sets of randomly selected NPs, as outlined in the Method section. We found that there are statistically more NPLDs in most of the DCs than expected by chance, with 78.3% of the DCs having P ≤ 0.0095 and additional 10% of the DCs having 0.011 ≤ P ≤ 0.0362 respectively (Table 2), which correspond to very strong (P ≤ 0.01) and strong (0.01 < P ≤ 0.05) presumption against null hypothesis respectively^52^. The P-value of the remaining 4 (6.7%) and 3 (5%) DCs are in the range of 0.0525 ≤ P ≤ 0.0736 and 0.1233 ≤ P ≤ 0.1857 respectively, which correspond to low (0.05 < P ≤ 0.1) and no (P > 0.1) presumption against null hypothesis respectively^52^. It is noted that each of these seven DCs has only 2 NPLDs and there is a possibility that the low statistical significance of these DCs are partly due to the few discovered NPLDs in these DCs.

To further provide a more conservative evaluation of the statistical significance of the clustering of NPLDs in the DCs, Bonferroni correction for study-wide hypothesis testing was performed. We found that, under the Bonferroni correction with α′ = 0.05/60, 48.3% DCs still have statistically strong or very strong significance against null hypothesis (Table 2). On the other hand, 16.7% DCs showed weak and 35% DCs showed no significance. It is noted that the majority (67.7%) of these weak or no significance DCs have 2–3 NPLDs in their respective DCs. The low number of NPLDs in each of these DCs likely leads to a higher tendency of forming a distribution pattern with weaker statistical significance that can be exposed by stricter statistic tests.

### Molecular mechanisms that contribute to the clustering of NPLDs

To determine what molecular mechanisms might contribute to the clustering of NPLDs within individual DCs with particular focus on the possible influence of the targets of their derived drugs, we evaluated the 203 targets of the 822 approved and clinical trial drugs of the 331 NPLDs in the 55 DCs with their target information available in the therapeutic target database^53^. We found that the targets of each individual DC are primarily from one to a few target classes (e.g. amine receptors) with their substrates/ligands from one to a few chemical classes (e.g. amines). This finding is based on the limited target information for 74.9% NPLDs and without considering the additional targets of the non-NPLDs in each DC. While the limited target information may not enable a full investigation of the influence of drug target-sites, it nonetheless provides useful hints about the key factors that promote the clustering of NPLDs. The 203 targets can be classified based on their target-sites into 45 target-site classes (TCs) labeled as TC1 to TC45, which collectively belong to 20 target-site super-classes (TSs) labeled as TS1 to TS20 (Supplementary Table S7). A TS is defined as a group of target-sites bound by substrates/ligands of a specific chemical class (e.g. amine binding sites) irrespective of their targets. A TC represents a sub-group of target-sites of a specific target class (e.g. amine transporters) bound by substrates/ligands of a specific chemical class.

**Figure 3:**
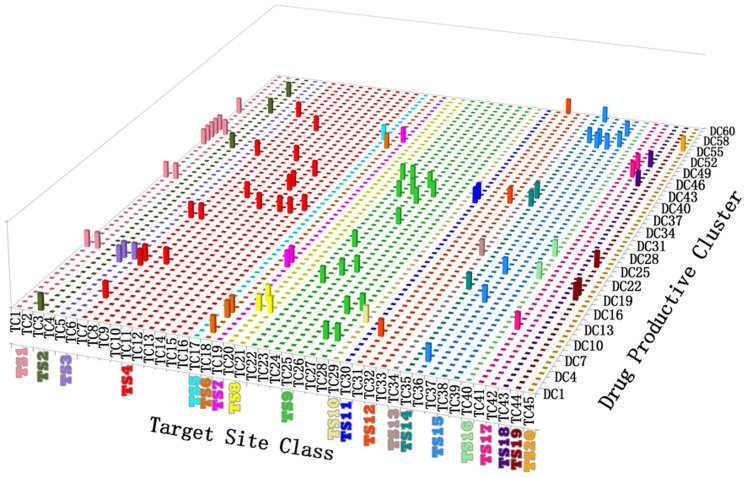
Distribution of the approved NP-related drugs, grouped into 45 target-site classes (TCs) of 20 target-site super-classes (TSs), in the drug-productive clusters DC1 to DC60. TSs are colored as: TC1, TC2 of TS1 amine sites (LightCoral), TC3, TC4 of TS2 nucleobase sites (OliverGreen), TC5, TC6 of TS3 nucleoside sites (PalePurple), TC7-TC16 of TS4 nucleoside phosphate sites (Red), TC17 of TS5 cyclic nucleotide sites (Cyan), TC18 of TS6 aminoacyl-tRNA sites (Chocolate), TC19 of TS7 amino acid phosphate sites (Magenta), TC20, TC21 of TS8 amino acid sites (Yellow), TC22-TC28 of TS9 oligopeptide sites (Green), TC29 of TS10 peptidoglycan sites (PaleYellow), TC30 of TS11 peptidoglycan sites (Blue), TC31-TC33 of TS12 saccharide sites (OrangeRed), TC34 of TS13 cyclic oligosaccharide drug delivery systems (PaleBrown), TC35 of TS14 lipopolysaccharide sites (DarkCyan), TC36-TC39 of TS15 fatty acid, cannabinoid, eicosanoid, retinoid sites (PaleBlue), TC40 of TS16 coenzyme A & analog sites (PaleGreen), TC41, TC42 of TS17 microtubule sites (DeepPink), TC43 of TS18 opiate sites (Purple), TC44 of TS19 steroid sites (Brown), and TC45 of TS20 naphthoquinone sites (Orange).

The targets in 53 (96.4%) DCs are from 1–3 TCs (27, 19, 7 DCs from 1, 2, 3 TCs) with the majority (65.5%) from either 1 TC (27 DCs) or 2–3 TCs of 1 TS (9 DCs), and the remaining 2 DCs are from 4 TCs (Figure 3). This indicates that the similar target-site structural constraints are likely the key factors in promoting the clustering of NPLDs in individual DCs. The targets of approved and clinical trial drugs are highly selective in their numbers, druggability features, and systems profiles^48^,^53^,^54^,^55^, and the druggability features have been characterized by the affiliation of the family members of the studied target to the known drug targets^56^ and by the existence of a privileged binding site with unique physicochemical properties^57^ for enabling favorable binding by drug-like molecules^58^.

Our revealed links between the clustering of NPLDs in individual DCs and the grouping of their targets in selected TCs are consistent with these findings. NPLDs in these DCs possess structural, physicochemical and/or pharmacophore features complementary to a privileged target-site, are at or near activity peaks against the target, and have good or amendable safety and pharmacokinetic properties. They either have or may be further optimized to gain such additional features as adequate metabolic stability^59^, metabolite safety^60^, absorption^61^ and physical forms^62^ to reach the drug sweet spots^63^ in the chemical space. Therefore, our revealed clustered patterns of NPLDs and their links to the selected TCs provide useful information and enable further study of the distribution profiles of the NPLDs in the chemical space particularly with respect to the relevant target-site classes.

Consistent with the reported clustering of GPCR, kinase and protease targeting agents in the chemical space^1^, the GPCR, kinase and protease TCs are primarily targeted by the selected chemical classes of NPLDs in specific DCs. For GPCRs, amine receptors (TC1) are primarily targeted by amines (DC31, DC44), ergoline alkaloids (DC40, DC41), and indole (DC14, DC42) and tropane (DC43) alkaloids, amino acid receptors (TC20) by amino acids (DC7) and oligopeptides (DC9), cannabinoid receptors (TC39) by cannabinoids (DC56) and cannabidiols (DC53), purine nucleoside receptors (TC5) by purines (DC12, DC13), opiate receptors (TC43) by opiate alkaloids (DC49), and monosaccharide receptors (TC32) by phenylpropanoids (DC60). Kinases (TC11) are primarily targeted by staurosporines (DC39). For the proteases, serine endopeptidases (TC28) are primarily targeted by glycosaminoglycans (DC10) and linear amino acid derivatives (DC4), proteasome (TC27) by oligopeptides (DC9), and exopeptidases (TC23) by phenethylamines (DC34), sesquiterpenes (DC22), larger indole alkaloids (DC14), and linear and cyclic peptides (DC38).

The other drug-prolific DCs are also closely linked to specific TCs (Table 1), with DC19 (steroids) linked to the nuclear receptor ligand binding sites (TC44), DC5 (aminoglycosides) to the DNA metabolism enzyme nucleoside phosphate (TC7) and ribosome 30 s subunit aminoacyl-tRNA (TC18) binding sites, DC17 (acarviosins) to the phosphatase substrate (TC19), ribosome 23S rRNA peptidyl transferase (TC25) and outer membrane lipopolysaccharide (TC35) sites, DC21 (fatty acids, prostanoids) to the retinoid receptor ligand (TC37) and coenzyme A analog metabolism enzyme substrate (TC40) binding sites, DC28 (cardiac glycosides) to the nucleoside phosphate metabolism enzymes substrate binding sites (TC10), and DC8 (β-lactams) to the β-lactam binding protein peptidoglycan binding sites (TC29).

### Detailed analysis of the physicochemical landscape of the NPLD distribution profile reveals clues for searching the sweet spots in the DCs

While the knowledge of the clustered distribution patterns of the NPLDs in the DCs and the correlation to the TCs is useful for revealing the NPLD-like structural frameworks for targeting specific target classes, more detailed analysis is needed for identifying the NPLDs within each DC. On the other hand, drug-like^14^,^41^,^42^,^43^ and lead-like^43^,^44^ rules have been derived and extensively used for identifying drug leads on the basis of whether their specific physicochemical properties fall into certain drug-likeness or lead-likeness ranges. Optimal hydrophobic and hydrogen bond interactions, and thus the AlogP/logD and ON/HD/HA values, are important for optimizing NPLDs into drugs^64^. Therefore, additional clues for searching the new NPLDs may be obtained by studying the physicochemical landscapes of the known NPLDs and the NPs in the DCs.

We evaluated the physicochemical landscape of the NPLDs and NPs in branch 9 characterized by the eight physicochemical properties MW, AlogP, logD, ON, HD, HA, RB, RI, and PZ frequently used for analyzing drug-like^14^,^41^,^42^,^43^ and lead-like^43^,^44^ features (Supplementary Figure S13). This branch contains four DCs (DC16, DC17, DC18, and DC19). While, the NPs inside and outside these DCs have mixed MW values, there is a significantly higher concentration of NPs with either higher AlogP/logD values or higher ON/HD/HA values. In particular, regardless of their MW values, the NPLDs tend to be located at the peak of either AlogP/logD or ON/HD/HA. For instance, the NPLDs in DC17 (which include macrolides, polyenes, spinosyns and acarviosins) have peak MW values likely due to the added or enlarged hydrophobic groups (peak AlogP/logD values) to optimally interact with, e.g., the outer membrane lipopolysaccharide sites of TC35, or the added hydrogen bonding components (peak ON/HD/HA values) to optimally interact with, e.g., the phosphatase substrate sites of TC19. The NPLDs in DC19 (composed of steroids and derivatives) tend to have peak AlogP or logD values without significantly enlarged MW values over other NPs in the DC, possibly due to enhanced hydrophobic components within the steroid structural framework for achieving optimal hydrophobic interactions with the nuclear receptor ligand sites of TC44. Therefore, the tendency of the NPLDs to be located at either the AlogP/logD or the ON/HD/HA peaks in the DCs may be potentially used as an indicator for searching new NPLDs.

### The distribution profiles of NPLDs with respect to bioactive NPs

The more clustered distribution of NPLDs (Figure 2 and Supplementary S7–10) are in contrast to the much less clustered distribution of the 48,216 bioactive NPs from the TCM@Taiwan database^25^ and the literatures^3^ (Supplementary Figures S14–17). Although the number and diversity of our collected bioactive NPs are limited in representing bioactive NPs, useful indications may be revealed. These bioactive NPs are more diversely distributed in 32 of the 33 branches with 78.8% of the bioactive NPs located outside the DCs. Therefore, NPLDs are distinguished from bioactive NPs in their tendency to more closely cluster together in the chemical space, which is consistent with the distribution pattern of drug-productive species families in the phylogenetic tree (drug-productive species families are more closely clustered than the species families of bioactive NPs)^3^. To investigate whether the more clustered distribution of NPLDs in the DCs is due to the more extensive exploration efforts towards these DCs, the exploration times of the 442 NPLDs, crudely estimated by the time since the first literature report, were compared to those of the 11,816 bioactive NPs inside and outside the DCs, which are largely comparable to each other (Supplementary Figures S18–21). Hence, there is no clear indication to link drug-productivity of the DCs to the biased exploration efforts.

### The distribution profiles of NPLDs with respect to time and disease classes

Since 1988, the number of DCs has been gradually increased at an average rate of 3.2 new DCs per 5 years, and the majority (60.0%–69.0%) of the 15–32 new NPLDs approved in every five-year period from 1560 to 2012 are from preexisting DCs (Table 3). Drug discovery focus has been shifting in terms of targets, chemotypes, diseases and therapeutic strategies^48^,^65^. To study if novel drugs derived from shifted focuses are outside pre-existing DCs, we analyzed 27 new NPLDs approved in 1990–2012 each targeting a novel target previously unaddressed by an approved drug (Supplementary Table S8) and thus are novel NPLDs of the time. At the time of their first drug approval, 18 (66.7%) of these novel NPLDs were from preexisting DCs, suggesting that existing DCs remain good sources of novel NPLDs and drugs.

The approved drugs from individual DCs largely target one to a few disease classes (Supplementary Figure S22). Specifically, 61.7% of DCs target one (DC1, DC2, DC11, DC18, DC23, DC26, DC39, DC46, DC51 and DC60), two (DC3, DC6, DC16, DC20, DC22, DC25, DC30, DC34, DC35, DC37, DC50, DC54, DC55, DC56, DC57 and DC59) or three (DC4, DC9, DC15, DC28, DC31, DC32, DC41, DC47, DC48, DC52 and DC58) disease classes. While the remaining DCs target multiple disease classes, most drugs from these DCs target a few disease classes. Anti-infectious and anti-parasite drugs are mostly from DC8 (87 drugs), DC5 (22 drugs), DC17 (19 drugs), DC45 (10 drugs), DC4 (9 drugs) and DC13 (8 drugs). Anticancer drugs are primarily from DC19 (31 drugs), DC13 (13 drugs), DC50 (9 drugs), DC38 (8 drugs), DC5 (7 drugs), DC14 (5 drugs) and DC46 (5 drugs). Circulatory system drugs are largely from DC38 (17 drugs), DC10 (12 drugs), DC28 (11 drugs), DC13 (9 drugs), DC44 (9 drugs) and DC21 (6 drugs). Nervous system drugs are mostly from DC49 (13 drugs), DC44 (7 drugs) and DC7 (5 drugs). Drugs for endocrine, nutritional and metabolic diseases are primarily from DC19 (13 drugs), DC26 (7 drugs), DC38 (6 drugs) and DC25 (5 drugs). Genitourinary system drugs are mostly from DC19 (15 drugs) and DC8 (5 drugs).

### The usefulness of the knowledge of NPLD distribution profile for facilitating new NPLD discovery

The tendencies of NPLDs to cluster together in the chemical space and to preferentially bind to the privileged target-sites in the target-space may be explored for assessing the development potential of new NP leads. Based on the insights derived from our analysis, one can postulate that, apart from the ability of an NPLD or its derivatives to modulate a validated target, an NPLD may have a higher probability to be developed into a drug if it is inside a DC, near a DC (to form an expanded DC) or near an NPLD outside existing DCs (to form a new DC) in the chemical space, and if its target belongs to an existing TC or a new TC in an existing TS. This postulation was tested by the retrospective analysis of the new NPLDs of FDA approved drugs in 2013–June 2014^66^,^67^ that were not used in the derivation of the NPLD distribution patterns and the target-site linkages. Our literature search led to the finding of 4 new small molecule NPLDs, 3 of which were retrospectively recognized as developable based on our postulation (Table 4). Specifically, the NPLD uridine monophosphate of sofosbuvir is inside DC5 and target TC7, the NPLD phlorizin of canagliflozin is near DC57 (*Tc* = 0.91 to the nearest NPLD) and target monosacharide transporter substrate sites as a new TC in TS12 (saccharide binding sites), and the imidazole-based NPLD (e.g. mizoribine) of luliconazole is inside DC13 and target a steroid metabolism enzyme substrate site in TS19 (steroid binding sites). Therefore, the insights derived from this and other studies of NPLD distribution profiles may be explored for facilitating the assessment of the development potential of NP leads.

## Concluding Remarks

This study systematically exposed the clustered distribution profiles of NPLDs and revealed useful insights into the mechanisms that partly contribute to the formation of these profiles, i.e., the tendency of NPLDs to preferentially bind to the privileged target-sites. The insights from this and other studies of NPLD distribution profiles provide useful clues to and enable further studies of the lead sweet spots in the chemical space with respect to the corresponding target-sites. The distribution of NPLDs and the lead sweet spots in the chemical space is collectively influenced by potent binding to the target-sites and such additional factors as the optimization potential to reach the drug sweet spots in the chemical space^63^ with more adequate metabolic stability^59^, metabolite safety^60^, absorption^61^ and physical forms^62^). Further studies are needed for a deeper understanding of the collective influence of these multiple factors on the distribution of NPLDs in the chemical space. These advances coupled with expanded knowledge of lead-like and drug-like structures and physicochemical properties^13^ may enable more prioritized and rational exploration of the NP-subspaces for drug discovery.

## Supplementary Material

Supplementary Information Supplementary Information

## Figures and Tables

**Table 1:** Top-ranked drug lead productive clusters with higher number of approved leads in the natural product chemical space represented by 137,836 natural products and 442 natural product leads. The corresponding target site class(es) and superclass(es) are also listed

Drug Lead Cluster (Branch)	Drug Lead Molecular Scaffold Groups	No of NPs	No of Leads, Drugs (Approved/Trial)	Target Site Super Class	Target Site Class
DC19 (14)	Steroids & derivatives	546	32/5, 85/8	steroid sites	Nuclear receptor ligand sites
DC5 (3)	Pyrimidine nucleosides, Aminoglycosides	225	19/0, 32/0	nucleoside phosphate sites, aminoacyl-tRNA sites	DNA metab enzymes nucleoside phosphate, ribosome 30 s aminoacyl-tRNA sites
DC13 (4)	Purine nucleosides, Imidazole analogs & their oligopeptide hybrids	264	17/2, 39/22	Nucleoside sites, nucleoside phosphate sites	nucleoside receptor ligand & metabolism enzyme substrate sites, Nucleoside phosphate receptor ligand, DNA metab enzymes nucleoside phosphate sites
DC7 (3)	Amino acids with acyclic hydroxyl side chain & derivatives	217	15/0, 23/0	amino acid sites	amino acid receptor ligand & metab enzyme substrate sites
DC17 (14)	Macrolides, Polyenes, Spinosyns, Acarviosins	117	15/4, 29/14	amino acid phosphate sites, oligopeptide sites, lipopolysaccharide sites	phosphatase substrate, ribosome 23S peptidyl transferase, outer membrane lipopolysaccharide sites
DC21 (14)	Fatty acids & derivatives, Prostanoids	423	8/1, 26/1	fatty acid, cannabinoid, eicosanoid, retinoid, coenzyme A sites	retinoid receptor ligand, CoA metab enzyme substrate sites
DC28 (17)	Cardiac glycosides	176	8/1, 12/1	nucleoside phosphate sites	nucleoside phosphate metab enzyme substrate sites
DC38 (21)	Intermediate-sized linear and cyclic peptides	33	7/1, 32/2	Oligopeptide sites, lipopolysaccharide sites	exopeptidase substrate, Neuropeptide receptor ligand, outer membrane lipopolysaccharide sites
DC8 (3)	Beta-lactams	92	6/2, 90/6	peptidoglycan sites	ß-lactam binding protein peptidoglycan sites
DC29 (20)	Saponins, Triterpenoid glycosides, Macrocyclic lactones	1696	6/1, 7/2	nucleoside phosphate sites	steroid metab enzyme nucleoside phosphate, calcium channel DHP, chloride channel CBS sites
DC45 (30)	Tetracyclines, Capsaicinoids, Disulfide bromotyrosine derivatives	447	6/3, 13/7	aminoacyl-tRNA sites	ribosome 30 s aminoacyl-tRNA sites
DC49 (32)	Opiate alkaloids, Phenanthrene alkaloids	457	6/1, 18/1	amine sites, opiate sites	amine receptor ligand, opiate receptor ligand sites
DC10 (3)	Glycosaminoglycans, glucosamines, Lincosamides & derivatives	237	5/0, 17/0	oligopeptide sites	serine endopeptidase substrate sites
DC12 (4)	Purine base analogs, modified purine base analogs	180	5/0, 9/0	nucleoside sites, nucleoside phosphate sites	nucleoside receptor ligand, DNA metab enzymes nucleoside phosphate sites
DC14 (7)	Larger indole alkaloids	4104	5/1, 15/16	amine sites, oligopeptide sites	amine receptor ligand & transporter substrate, exopeptidase substrate sites
DC53 (10)	Cannabinoids, Diarylheptanoids, Dihydrostilbenoids, Small phenolic molecules with a long tail	2531	5/4, 9/8	fatty acid, cannabinoid, eicosanoid, retinoid sites	fatty acid metab enzyme substrate, retinoid receptor ligand, cannabinoid receptor ligand sites
DC24 (16)	Oligo-, Poly-, Cyclic- saccharides	259	5/0, 5/0	cyclic oligosaccharide drug delivery systems	cyclodextrin drug delivery systems
DC36 (20)	Large cyclic peptides	265	5/6, 6/7	nucleoside phosphate sites, sites within peptidoglycans, saccharide sites, lipopolysaccharide sites	calcium channel DHP, cell wall peptidoglycan, polysaccharide metab enzyme substrates, outer membrane lipopolysaccharide sites
DC40 (24)	Porphyrins, Prodiginines, Ergoline-, Ellipticine-, Epibatidine- alkaloids	519	5/3, 8/4	amine sites, nucleobase sites	amine receptors ligand, DNA intercalation sites
DC42 (26)	Indole-containing amino acid tryptophan analogs, Monoterpenoid indole alkaloids, Yohimbine alkaloids	512	5/2, 6/4	amine sites	amine receptor ligand sites
DC43 (28)	Tropane alkaloids	29	5/0, 10/0	amine sites	amine receptor ligand & transporter substrate sites
DC44 (28)	Catecholamines, Small alkaloids with an amine group	358	5/0, 21/0	amine, opiate sites	amine receptor, opiate receptor ligand sites

**Table 2:** The statistical significance of the clustering of the NPLDs in every DC. MTd is the mean Tanimoto distance of the NPLDs in each DC, MTd.rnd is the mean Tanimoto distance in randomization, NRI is a standardized effect size measure of the community structure, and P-value is the number of randomly selected NPs that are more clustered than the NPLDs in each DC divided by the number of runs (60,000 in this study). P values in bold are the ones which remain significant after Bonferroni correction with conservative α′ = 0.05/60 = 0.000833

DC	Branch	No of NPLD	MTD	MTD.rnd	NRI	P-value
DC1	1	2	0.2642	1.8368	6.2835	0.00238
DC2	1	2	0.1224	1.8373	6.9228	0.00117
DC3	1	2	0.6296	1.8331	4.7303	0.00847
DC4	3	4	1.0322	1.6705	6.1005	**0.00047**
DC5	3	19	1.2111	1.6704	16.3408	**0.00000**
DC6	3	5	1.2328	1.6704	5.2077	0.00142
DC7	3	15	1.4909	1.6706	5.2466	**0.00043**
DC8	3	6	1.0778	1.6706	8.2775	**0.00003**
DC9	3	7	1.1073	1.6711	9.1502	**0.00002**
DC10	3	5	0.8436	1.6704	9.8633	**0.00000**
DC11	4	4	0.8826	1.5121	4.7221	0.00277
DC12	4	5	0.6238	1.5122	8.4309	**0.00000**
DC13	4	18	1.0751	1.5107	12.7659	**0.00000**
DC14	7	5	0.8233	1.2803	4.4320	0.00177
DC15	8	3	0.8090	1.5069	4.7523	0.00175
DC16	9	3	0.4985	1.4882	5.2888	0.00145
DC17	9	16	0.8576	1.4892	10.2529	**0.00000**
DC18	9	2	1.0548	1.4913	1.5675	0.06920
DC19	9	37	1.0487	1.4896	11.3267	**0.00000**
DC20	10	2	0.1740	1.4029	5.8662	0.00135
DC21	10	9	0.9145	1.4017	6.8067	**0.00000**
DC22	10	3	0.7339	1.4007	4.5322	**0.00078**
DC23	10	2	1.2542	1.3999	0.6864	0.15480
DC24	11	5	0.8121	1.3910	5.5223	0.00100
DC25	12	2	0.2934	1.2207	3.7910	0.00947
DC26	12	2	0	1.2202	4.9798	**0.00078**
DC27	12	8	0.9926	1.2208	2.9058	0.01428
DC28	12	8	0.2007	1.2200	12.9182	**0.00000**
DC29	12	7	0.7426	1.2203	5.5655	**0.00043**
DC30	13	2	0.8474	1.3667	1.9145	0.06510
DC31	14	5	1.0297	1.5449	4.2627	0.00100
DC32	14	5	0.9406	1.5439	4.9565	**0.00007**
DC33	14	4	1.1776	1.5454	2.5820	0.02413
DC34	14	3	0.8209	1.5460	4.0057	0.00120
DC35	14	6	0.6607	1.5452	8.3548	**0.00000**
DC36	15	10	0.6860	1.3351	7.9798	**0.00000**
DC37	16	3	0.6935	1.1435	3.4132	0.01103
DC38	16	7	0.5400	1.1431	10.9642	**0.00000**
DC39	17	3	0.4787	1.1665	5.0540	0.00093
DC40	17	7	0.8464	1.1671	4.7313	0.00235
DC41	17	2	0.6630	1.1649	2.3529	0.03623
DC42	17	7	0.7352	1.1663	6.3612	**0.00025**
DC43	20	5	0.4329	1.3241	17.2567	**0.00000**
DC44	20	5	0.6912	1.3241	12.1159	**0.00003**
DC45	20	7	0.9507	1.3238	10.0919	**0.00002**
DC46	21	3	0.1372	0.7713	5.0125	**0.00010**
DC47	21	4	0.5792	0.7708	1.8932	0.02985
DC48	24	4	0.7416	1.0739	5.7931	0.00110
DC49	24	6	0.5427	1.0740	14.5890	**0.00000**
DC50	26	5	0.4218	0.7916	6.3884	**0.00012**
DC51	26	2	0.6052	0.7909	1.1859	0.07355
DC52	28	3	0.9049	1.1883	2.4123	0.03025
DC53	28	9	0.9577	1.1880	5.9241	**0.00070**
DC54	30	2	0.7262	0.8764	0.7311	0.18568
DC55	32	3	0.3393	0.8538	4.4925	0.00152
DC56	32	2	0.6884	0.8548	0.9532	0.12325
DC57	32	2	0.2012	0.8550	3.7418	0.00427
DC58	32	2	0.4916	0.8533	2.0560	0.05248
DC59	32	3	0.2995	0.8535	4.8258	**0.00077**
DC60	33	2	0	0.9059	5.0937	**0.00040**

**Table 3:** chronological data of the natural product leads with the first approved drugs and the drug lead productive clusters during every five-year period from 1963 to 2012. The six drug lead clusters with only one approved drug plus one or more clinical trial drugs were not included here

	Number of natural product leads with the first approved drug in period		Number of drug lead clusters in period	
Period	Inside preexisting DCs	Outside preexisting DCs	Number of preexisting DCs	Number of new DCs
Pre–1963	56	NA	8	NA
1963–1967	7	20	8	7
1968–1972	4	9	15	3
1973–1977	9	8	18	2
1978–1982	17	56	20	13
1983–1987	20	20	33	5
1988–1992	20	12	38	6
1993–1997	20	9	44	2
1998–2002	19	10	46	3
2003–2007	9	6	49	1
2008–2012	11	6	50	4

**Table 4:** The list of natural product derived drugs approved by FDA from 2013 to 2014 June. The natural product leads, targets and affiliations to drug productive clusters and target-site classes are provided

Drug	Drug Type	NP Lead	NP Lead Type	Affiliation to drug lead productive cluster	target	Affiliation to target-site class
Canagliflozin	Small Molecule	Phlorizin	N	Near DC57 (tanimoto similarity coefficient 0.91 to the nearest NPLD)	SGLT2	Monosacharide transporter substrate sites as a new TC in TS12 (saccharide binding sites)
Luliconazole	Small Molecule	Imidazole-based NP such as mizoribine	N	DC13	Lanosterol demethylase	Steroid metabolism enzyme substrate sites in TS19 (steroid binding sites)
Sofosbuvir	Small Molecule	Uridine monophosphate	N	DC5	HCV NS5B polymerase	TC7
Vorapaxar	Small molecule	Himbacine	N	-	Protease-activated receptor-1	TC22
Simeprevir	Oligopeptide	HCV NS3/4A protease product oligopeptide	B (oligopeptide)	DC9	HCV NS3/4A protease	TC28
Mipomerse	Antisense	Section of mRNA of apolipoprotein B-100	B (oligonucleotide)	-	Apolipoprotein B-100	Lipid-binding sites in TS15
Dalvance	Semisynthetic lipoglycopeptide	Lipoglycopeptide	B (lipoglycopeptide)	-	Cell wall	TC30
Tanzeum	Peptide	Glucagon-like peptide-1	B (peptide)	-	Glucagon-like peptide-1 receptor	TC22
