# Supplementary material for: Clustered Distribution of Natural Product Leads of Drugs in the Chemical Space as Influenced by the Privileged Target-Sites
Source: Sci Rep. 2015 Mar 20;5:9325. doi: 10.1038/srep09325 (PMC5380136; doi:10.1038/srep09325)

Supplementary Tables and Figures for

Clustered Distribution of Natural Product Leads of Drugs in the Chemical Space as Influenced by the Privileged Target-Sites

Lin Tao[1,2,3], Feng Zhu[1,2,4], Chu Qin[2,3], Cheng Zhang[2], Shangying Chen[2], Peng Zhang[2], Cunlong Zhang[1], Chunyan Tan[1], Chunmei Gao[1], Zhe Chen[5], Yuyang Jiang[1]*, and Yu Zong Chen[1,2,3]*

[1]*Department of Pharmacology and Pharmaceutical Sciences, School of Medicine, Tsinghua University, Beijing, P. R. China, and the Ministry-Province Jointly Constructed Base for State Key Lab-Shenzhen Key Laboratory of Chemical Biology, the Graduate School at Shenzhen, Tsinghua University, Shenzhen, P. R. China*

[2]*Bioinformatics and Drug Design Group, Department of Pharmacy, and Center for Computational Science and Engineering, National University of Singapore, Singapore 117543*

[3]*NUS Graduate School for Integrative Sciences and Engineering, Singapore 117456*

[4]*Innovative Drug Research Centre and College of Chemistry and Chemical Engineering, Chongqing University, Chongqing, P. R. China*

[5]*Zhejiang Key Laboratory of Gastro-intestinal Pathophysiology, Zhejiang Hospital of Traditional Chinese Medicine, Zhejiang Chinese Medical University, Hangzhou, P. R. China*

Corresponding Authors: Y.Z. Chen. Tel.: 65-6874-6877. Fax: 65-6774-6756. E-mail: phacyz@nus.edu.sg; Y.Y. Jiang. Tel.: 86- 0755-26036017. Fax: 86- 0755-26036017. E-mail: Jiangyy@sz.tsinghua.edu.cn

Supplementary Table S1 List of FDA approved drugs in 2008-2012. The natural product-related drugs are marked based on their types as follows: N natural product, ND derived from a natural product and is usually a semisynthetic modification, NM natural product mimic, B non human biologics include peptides, nucleic acids, proteins, and antibodies, B* human biologics include peptides, nucleic acids, proteins, and antibodies, S* made by total synthesis based on the pharmacophore from a natural product.

Drug Generic Name	Natural Product Related Drug Type	Lead of Natural Product Related Drug	Targeted Disease	Year of Approval
Aclidinium bromide			Bronchospasms associated with COPD	2012
Apixaban			Stroke and systemic embolism in patients with non-valvular atrial fibrillation	2012
Avanafil			Erectile dysfunction	2012
Axitinib			Advanced renal cell carcinoma	2012
Bedaquiline fumarate			Pulmonary multidrug-resistant tuberculosis	2012
Bosutinib monohydrate			Chronic, accelerated or blast phase Ph+ chronic myelogenous leukaemia	2012
Cabozantinib s-malate			Progressive, metastatic medullary thyroid cancer	2012
Carfilzomib	ND	Epoxomicin	Multiple myeloma	2012
Citric acid; Magnesium oxide; Sodium picosulfate			Cleansing of the colon before colonoscopy	2012
Cobicistat; Elvitegravir; Emtricitabine; Tenofovir disoproxil fumarate			HIV-1 infection	2012
Crofelemer	N	Crofelemer	HIV-associated diarrhoea	2012
Enzalutamide			Metastatic castration-resistant prostate cancer	2012
Glucarpidase	B*	Carboxypeptidase G2	Toxic plasma methotrexate concentrations in patients with delayed methotrexate clearance	2012
Ingenol mebutate	N	Ingenol mebutate	Actinic keratosis	2012
Linaclotide	B*	Linaclotide	Irritable bowel syndrome with constipation; chronic idiopathic constipation	2012
Lorcaserin hydrochloride			Obesity	2012
Lucinactant	ND	Lucinactant	Prevention of respiratory distress syndrome	2012
Mirabegron			Overactive bladder	2012
Ocriplasmin	B*	Ocriplasmin	Symptomatic vitreomacular adhesion	2012
Omacetaxine mepesuccinate	N	Homoharringtonine	Chronic or accelerated-phase chronic myeloid leukaemia	2012
Pasireotide diaspartate	ND	Somatostatin	Cushing's disease	2012
Peginesatide acetate	NM	Peginesatide	Anaemia due to chronic kidney disease	2012
Pertuzumab	B*	Pertuzumab	HER2-positive metastatic breast cancer	2012
Raxibacumab	B*	IgG1λ	Anthrax	2012
Regorafenib			Metastatic colorectal cancer	2012
Tafluprost	ND	Prostaglandin E1	Elevated intraocular pressure in patients with open-angle glaucoma or ocular hypertension	2012
Taliglucerase alfa	B*	Taliglucerase alfa	Gaucher's disease	2012

Tbo-filgrastim	B*	Granulocyte colony-stimulating factor	Severe neutropaenia in patients with non-myeloid malignancies	2012
Teduglutide	B*	Glucagon-like peptide-2	Short bowel syndrome	2012
Teriflunomide			Relapsing forms of multiple sclerosis	2012
Tofacitinib citrate			Severely active rheumatoid arthritis	2012
Vismodegib	S*	Cyclopamine	Metastatic or locally advanced basal cell carcinoma	2012
Ziv-aflibercept	B*	VEGFR1, VEGFR2, IgG	Metastatic colorectal cancer	2012
Ivacaftor			Cystic fibrosis in patients with the G551D mutation in the CFTR gene	2012
Lomitapide mesylate			Homozygous familial hypercholesterolaemia	2012
Perampanel			Partial-onset seizures in patients with epilepsy	2012
Ponatinib hydrochloride			Chronic, accelerated or blast-phase chronic myeloid leukaemia	2012
Abiraterone	ND	Pregnenolone	Metastatic castration-resistant prostate cancer	2011
Aflibercept	B*	Fusion protein (chimeric protein)	Neovascular (wet) age-related macular degeneration	2011
Asparaginase erwinia chrysanthemi	B*	Asparaginase	Acute lymphoblastic leukaemia	2011
Azilsartan kamedoxomil			Hypertension	2011
Belatacept	B*	A fusion protein of the Fc fragment of a human IgG1 immunoglobulin linked to the extracellular domain of CTLA-4	Organ rejection in kidney transplant	2011
Belimumab	B*	Belimumab	Active, autoantibody-positive, systemic lupus erythematosus	2011
Boceprevir			HCV genotype 1	2011
Crizotinib			ALK-positive advanced or metastatic NSCLC	2011
Ezogabine			Partial-onset seizures	2011
Fidaxomicin	N	Fidaxomicin	Clostridium difficile-associated diarrhoea	2011
Icatibant	NM	Bradykinin	Hereditary angioedema	2011
Indacaterol maleate			COPD	2011
Ipilimumab	B*	Ipilimumab	Unresectable or metastatic melanoma	2011
Linagliptin	ND	Xanthine	Type 2 diabetes	2011
Rilpivirine	NM	Pyrimidine	HIV-1 infection	2011
Roflumilast			COPD exacerbations	2011
Ruxolitinib phosphate			Intermediate or high-risk myelofibrosis	2011
Spinosad	N	Spinosyn D	Head lice	2011
Telaprevir	NM	NS5A-5B substrate decamer peptide	HCV genotype 1	2011
Ticagrelor	ND	Adenosine triphosphate	Thrombotic cardiovascular events in patients with acute coronary syndrome	2011
Vilazodone hydrochloride			Major depressive disorder	2011
Brentuximab vedotin			Hodgkin's lymphoma; systemic anaplastic large cell lymphoma	2011
Clobazam			Seizures associated with Lennox–Gastaut syndrome	2011
Deferiprone			Transfusional iron overload due to thalassaemia syndromes	2011
Gabapentin enacarbil			Moderate-to-severe restless legs syndrome	2011

Rivaroxaban			Prophylaxis of deep vein thrombosis in hip and knee replacement surgery	2011
Vandetanib			Unresectable or metastatic medullary thyroid cancer	2011
Vemurafenib			BRAF-positive unresectable or metastatic melanoma	2011
Alcaftadine			Allergic conjunctivitis	2010
Alpha1-proteinase inhibitor	B*	Alpha1-proteinase inhibitor	Emphysema	2010
Cabazitaxel; Xrp-6258	ND	Taxol	Prostate cancer	2010
Carglumic acid	ND	A man-made form of an enzyme that occurs naturally in the liver	Acute hyperammonaemia	2010
Ceftaroline fosamil	ND	Cephalosporin	Skin and skin-structure infections; community acquired pneumonia	2010
Collagenase clostridium histolyticum	B	Collagenase clostridium histolyticum	Dupuytren's contracture	2010
Dabigatran; Bibr 953	S*/NM	Thrombin-interacting part of fibrinogen	Stroke prevention in atrial fibrillation	2010
Dalfampridine	ND	Pyridine	Improving walking in patients with multiple sclerosis	2010
Denosumab	B*	Denosumab	Postmenopausal osteoporosis?	2010
Dienogest; Estradiol valerate			Contraception	2010
Eribulin; E7389; Nsc-707389; Eribulin mesylate	ND	Halichondrin B	Breast cancer	2010
Fingolimod; Fty720	ND	Myriocin	Multiple sclerosis	2010
Immune globulin subcutaneous, 20% liquid	B*	Immunoglobulin G	Primary immunodeficiencies	2010
Incobotulinumtoxina	B	Incobotulinumtoxina	Cervical dystonia and blepharospasm	2010
Liraglutide	ND	Peptidomimetic of GLP-1 peptide	Type 2 diabetes	2010
Lurasidone hydrochloride			Schizophrenia	2010
Pegloticase	B*	Urate oxidase	Gout	2010
Tesamorelin	B*	Growth-hormone-releas ing hormone	HIV lipodystrophy	2010
Tocilizumab	B*	Tocilizumab	Rheumatoid arthritis	2010
Ulipristal	ND	Progesterone	Contraception	2010
Velaglucerase alfa	B*	β-glucocerebrosidase	Gaucher's disease	2010
Polidocanol			Uncomplicated spider veins and uncomplicated reticular veins	2010
Abobotulinum-toxin a	B	Abobotulinum-toxin a	Cervical dystonia and glabellar lines	2009
Artemether; lumefantrine	ND	Artemisinin	Malaria	2009
Asenapine maleate			Schizophrenia and bipolar disorder	2009
Benzyl alcohol			Head lice	2009
Bepotastine besilate			Allergic conjunctivitis	2009
Besifloxacin hydrochloride			Bacterial conjunctivitis	2009
Canakinumab	B*	Canakinumab	Cryopyrin-associated periodic syndromes	2009
Dronedarone hcl	ND	Khellin	Atrial fibrillation and atrial flutter	2009
Ecallantide	B	Aprotinin	Hereditary angioedema	2009
Everolimus; Rad-001; Sdz rad	ND	Sirolimus	Renal cell carcinoma	2009
Febuxostat			Hyperuricaemia in patients with gout	2009
Golimumab	B*	Golimumab	Rheumatoid arthritis, psoriatic arthritis and ankylosing	2009

			spondylitis	
Iloperidone			Schizophrenia	2009
Milnacipran hydrochloride			Fibromyalgia	2009
Ofatumumab	B*	Ofatumumab	Chronic lymphocytic leukaemia	2009
Pazopanib; Gw-786034	S/NM	Zeatin	Renal cell carcinoma	2009
Pitavastatin	S*/NM	Mevastatin	Hyperlipidaemia and mixed dyslipidaemia	2009
Pralatrexate	ND	Folic acid	Peripheral T cell lymphoma	2009
Romidepsin	N	Romidepsin	Cutaneous T cell lymphoma	2009
Saxagliptin hydrochloride			Type 2 diabetes	2009
Telavancin	ND	Vancomycin	Complicated skin and skin structure infections	2009
Ustekinumab	B*	Ustekinumab	Moderate-to-severe plaque psoriasis	2009
Vigabatrin	ND	Gamma-aminobutyric acid (GABA)	Infantile spasms in children aged 1 month to 2 years and complex partial seizures in adults	2009
Prasugrel hydrochloride			Reduction of thrombotic cardiovascular events in patients with acute coronary syndrome undergoing percutaneous coronary intervention	2009
Tolvaptan			Hypervolaemic and euvolaemic hyponatraemia, including patients with heart failure, cirrhosis and syndrome of inappropriate antidiuretic hormone	2009
Bendamustine hydrochloride			Chronic lymphocytic leukaemia	2008
Certolizumab pegol	B*	Certolizumab pegol	Crohn's disease	2008
Degarelix	NM	Gonadotropin-releasing hormone (GnRH decapeptide)	Advanced prostate cancer	2008
Desvenlafaxine	ND	Benzylamine	Major depressive disorder	2008
Difluprednate	ND	Cortisol	Inflammation and pain associated with ocular surgery	2008
Etravirine			HIV-1	2008
Fesoterodine fumarate			Overactive bladder disorder	2008
Fospropofol disodium			Monitored anaesthesia care sedation	2008
Lacosamide			Partial-onset seizures in epilepsy	2008
Methylnaltrexone bromide	ND	Morphine	Opioid-induced constipation	2008
Rilonacept	B*	Fc portion of human IgG1 combined with extracellular portions of IL-1R1 and IL-1RAcP	Cryopyrin-associated periodic syndromes including familial cold autoinflammatory syndrome and Muckle–Wells syndrome	2008
Romiplostim	B*	Thrombopoietin	Thrombocytopenia in patients with chronic immune (idiopathic) thrombocytopenic purpura	2008
Silodosin			Benign prostatic hyperplasia	2008
Tapentadol hydrochloride			Moderate to severe acute pain	2008
Alvimopan			To accelerate gastrointestinal recovery following bowel resection surgery	2008
Clevidipine			Peri-operative hypertension when oral therapy is not feasible or not desirable	2008
Eltrombopag olamine			Thrombocytopenia in patients with chronic immune (idiopathic) thrombocytopenic purpura	2008
Plerixafor			Autologous transplantation in patients with non-Hodgkin's lymphoma and multiple myeloma	2008
Rufinamide			Seizures associated with Lennox–Gastaut syndrome	2008
Tetrabenazine			Chorea associated with Huntington's disease	2008

Supplementary Table S2 List of the approved nature-related small molecule drugs, their natural product leads and the molecular scaffolds of the leads. Nature-related small molecule drugs include natural products and natural product semi-synthetic derivatives, mimetics, and pharmacophore-guided synthetic molecules.

Drug Name	Drug Lead	Lead Scaffold	Drug Type	Targeted Disease	Year of Approval
Vismodegib	Cyclopamine	Steroidal jerveratrum alkaloids	S*	Metastatic or locally advanced basal cell carcinoma	2012
Tafluprost	Prostaglandin E1	Prostaglandins	ND	Elevated intraocular pressure in patients with open-angle glaucoma or ocular hypertension	2012
Pasireotide diaspartate	Somatostatin	Somatostatin peptides ; GPCR targeting peptide hormones & analogues (cyclohexapeptides)	ND	Cushing's disease	2012
Carfilzomib	Epoxomicin	Epoxyketone containing oligopeptides	ND	Multiple myeloma	2012
Omacetaxine mepesuccinate	Homoharringtonine	Cephalotaxine alkaloids	N	Chronic or accelerated-phase chronic myeloid leukaemia	2012
Ingenol mebutate	Ingenol mebutate	Diterpenes	N	Actinic keratosis	2012
Crofelemer	Crofelemer	Proanthocyanidins	N	HIV-associated diarrhoea	2012
Telaprevir	NS5A-5B substrate decamer peptide	Decamer peptides	NM	HCV genotype 1	2011
Icatibant	Bradykinin	Bradykinin peptides (nonapeptides)	NM	Hereditary angioedema	2011
Rilpivirine	Pyrimidine	Pyrimidine analogues	NM	HIV-1 infection	2011
Abiraterone	Pregnenolone	Progestogens	ND	Metastatic castration-resistant prostate cancer	2011
Linagliptin	Xanthine	Purine analogues xanthine-type	ND	Type 2 diabetes	2011
Ticagrelor	Adenosine triphosphate	Purine nucleoside analogues	ND	Thrombotic cardiovascular events in patients with acute coronary syndrome	2011
Spinosad	Spinosyn A	Spinosyns	N	Head lice	2011
Spinosad	Spinosyn D	Spinosyns	N	Head lice	2011
Ceftaroline fosamil	Cephalosporin	Glycopeptides; Cephalosporins	ND	Skin and skin-structure infections; community acquired pneumonia	2010
Mifamurtide	Muramyl dipeptide	Peptidoglycan	ND	anticancer	2010
Cabazitaxel; Xrp-6258	Taxol	Taxanes	ND	Prostate cancer	2010
Vinflunine	Vincristine	Vinca alkaloids	ND	anticancer	2010
Laninamivir octanoate	N-Acetylneuraminic acid	Sialic acids	ND	antiviral	2010
Fingolimod; Fty720	Myriocin	Amino fatty acids	ND	Multiple sclerosis	2010
Ulipristal	Progesterone	Progestogens	ND	Contraception	2010
Zucapsacin	Capsaicin	Capsaicinoids	ND	osteoarthritis	2010
Dalfampridine	Pyridine	Pyridines	ND	Improving walking in patients with multiple sclerosis	2010
3,4-diaminopyridine	Pyridine	Pyridines	ND	Lambert-Eaton Myasthenic Syndr	2010

phosphate					
Pazopanib; Gw-786034	Zeatin	Purine analogues adenine-type	S/NM	Renal cell carcinoma	2009
Pitavastatin	Mevastatin	Statins	S*/NM	Hyperlipidaemia and mixed dyslipidaemia	2009
Vigabatrin	Gamma-aminobutyric acid (GABA)	Amino acids with acyclic hydroxyl side chain & derivatives	ND	Infantile spasms in children aged 1 month to 2 years and complex partial seizures in adults	2009
Nalfurafine hcl	Morphine	Opiate alkaloids	ND	uremic pruritis	2009
Dronedarone hcl	Khellin	Furanochromones	ND	Atrial fibrillation and atrial flutter	2009
Artemether; lumefantrine	Artemisinin	Trioxanes	ND	Malaria	2009
Everolimus; Rad-001; Sdz rad	Sirolimus	Macrolides	ND	Renal cell carcinoma	2009
Pralatrexate	Folic acid	Folate analogues	ND	Peripheral T cell lymphoma	2009
Romidepsin	Romidepsin	Depsipeptide cyclic structure	N	Cutaneous T cell lymphoma	2009
Ceftobiprole medocaril	Cephalosporin	Glycopeptides; Cephalosporins	ND	Antibacterial	2008
Methylnaltrexone bromide	Morphine	Opiate alkaloids	ND	Opioid-induced constipation	2008
Desvenlafaxine	Benzylamine	Benzylamines	ND	Major depressive disorder	2008
Difluprednate	Cortisol	Glucocorticoids	ND	Inflammation and pain associated with ocular surgery	2008
Dienogest	Progesterone	Progestogens	ND	endometriosis	2008
Artesunate; Amodiaquine; Asaq	Artemisinin	Trioxanes	ND	Antiparasitic	2008
Artesunate; Amodiaquine; Asaq	Quinine	5-aminoquinolines	ND	Antiparasitic	2008
Sugammadex sodium	γ-cyclodextrin	Cyclodextrins	ND	antidote	2008
Biolimus a9	Rapamycin	Macrolides	ND	restenosis	2008
Lapatinib; Lapatanib; Gw2016; Gsk572016	Zeatin	Purine analogues adenine-type	S/NM	Advanced or metastatic breast cancer	2007
Temsirolimus; Cci-779	Sirolimus	Macrolides	ND	Advanced renal cell carcinoma	2007
Nilotinib; Amn-107	Staurosporine	Staurosporine alkaloids	ND	Chronic myelogenous leukaemia	2007
Sphingosomal topotecan; Inx-0076	Camptothecin	Camptothecin analogues	ND	Oncological disease	2007
Ixabepilone; Bms-247550	Epothilone B	Polyketide macrolactones	ND	Advanced or metastatic breast cancer	2007
Doripenem; S-4661	Thienamycin	Carbapenam	ND	Urinary tract infections	2007
Anidulafungin; Ver-002; V-echinocandin; Ly-303366	Echinocandin B	Echinocandin-like lipopeptides || Echinocandins	ND	fungal infections	2007
Retapamulin; Sb-275833	Pleuromutilin	Pleuromutilin scaffold	ND	Impetigo	2007
Fluticasone furoate	Cortisol	Glucocorticoids	ND	antiallergic	2007
Trabectedin; Ecteinascidin; Ecteinascidin-743; Et-743	Trabectedin	Tetrahydroisoquinoline alkaloids	N	Oncological disease	2007
Lisdexamfetamine dimesylate	Lisdexamfetamine dimesylate	Phenethylamine class	ND	ADHD	2007
Sunitinib malate; Su11248	Zeatin	Purine analogues adenine-type	S/NM	Imatinib-resistant gastrointestinal stromal tumour and advanced renal cell carcinoma	2006
Dasatinib	Staurosporine	Staurosporine alkaloids	S/NM	Chronic myeloid leukaemia	2006
Rotigotine	Dopamine	Catecholamines	S/NM	Early stage idiopathic Parkinson's disease	2006
Darunavir; Tmc114	Pepstatin	Hexa-peptide with	S*/NM	HIV	2006

		unusual amino acid			
Decitabine	Deoxycytidine	Cytidine analogues	S*	Myelodysplastic syndrome	2006
Suberoylanilide hydroxamic acid (saha)	Trichostatin	Aniline dicarboxylic acids	ND	Cutaneous T-cell lymphoma	2006
Anecortave acetate	Cortisone	Steroid derivatives (Corticosteroids)	ND	Macular degeneration	2006
Lubiprostone	Alprostadil	Prostaglandins	ND	Constipation	2006
Polyphenon e	Polyphenon e	Catechins	N	External genital and perianal warts	2006
Sorafenib	Zeatin	Purine analogues adenine-type	S/NM	Advanced renal cell carcinoma	2005
Clofarabine	Adenosine	Purine nucleoside analogues	S*	Paediatric leukaemia	2005
Entecavir	Deoxyguanosine	Purine nucleoside analogues	S*	Chronic hepatitis B virus infection	2005
Nelarabine	Guanosine	Purine nucleoside analogues	S*	T-cell acute lymphoblastic leukaemia and T-cell lymphoblastic lymphoma	2005
Tigecycline; Gar-936	Tetracycline	Tetracyclines	ND	Complicated skin and skin structure infections and complicated intra-abdominal infections	2005
Pralmorelin	Growth hormone-releasing peptide	Growth hormone-releasing hormone families	ND	Treatment of GH deficiency	2005
Conjugated estrogens b	Estrogen	Anabolic-androgenic steroids	ND	Hormone replacement therapy	2005
L-alanyl-l-glutamine	Glutamine	Amino acids with acyclic hydroxyl side chain & derivatives	ND	Mucositis	2005
Ciclesonide	Glucocorticoid	Glucocorticoids	ND	Allergic rhinitis	2005
Thc-cbd; Dronabinol; Cannabidiol; Gw-1000-02	Tetrahydrocannabinol	Cannabinols	N	Neurological disease	2005
Omega-conotoxin mviia; Snx-iii; Snx-111; C1002; Ziconotide	Omega-conotoxin MVIIA	Conotoxins	N	Severe chronic pain	2005
Fumagillin; Sr-90144	Fumagillin	Bisabolane sesquiterpenes	N	Antiparasitic	2005
Paclitaxel-albumin; Abi-007; Paclitaxel nanoparticles	Paclitaxel	Taxanes	N	Oncological disease	2005
Erlotinib hydrochloride	Zeatin	Purine analogues adenine-type	S/NM	Non-small-cell lung cancer	2004
Abarelix	Gonadotropin releasing hormone (GnRH)	Gonadotropin-releasing hormone family peptides	S*/NM	Advanced prostate cancer	2004
Azacytidine	Cytidine	Cytidine analogues	S*	Myelodysplastic syndrome	2004
Pemetrexed disodium	Folic acid	Folate analogues	NM	Malignant pleural mesothelioma	2004
Pregabalin	Gamma-aminobutyric acid (GABA)	Amino acids with acyclic hydroxyl side chain & derivatives	ND	Diabetic peripheral neuropathy and post-herpetic neuralgia pain	2004
Talaporfin sodium	Chlorin	Chlorins	ND	anticancer	2004
Telithromycin	Erythromycin	Macrolides	ND	Respiratory infections	2004
Hexyl aminolevulinate	Hexyl aminolevulinate	Amino acids with acyclic hydroxyl side chain & derivatives	ND	Anticancer	2004
Vapreotide acetate	Somatostatin	Somatostatin peptides ; GPCR targeting peptide hormones & analogues	ND	Anticancer	2004

		(cyclohexapeptides)			
Estradiol; Levonorgestrel	Cholesterol	Sterols	ND	Hormone replacement therapy	2004
Estradiol; Levonorgestrel	Estradiol	Anabolic-androgenic steroids	ND	Hormone replacement therapy	2004
Belotecan hydrochloride; Camptobell; Cdk-602	Camptothecin	Camptothecin analogues	ND	Oncological disease	2004
Apomorphine hydrochloride	Morphine	Opiate alkaloids	N	Parkinson's disease	2004
Atazanavir; Bms-232632	Pepstatin	Hexa-peptide with unusual amino acid	S*/NM	HIV	2003
Fosamprenavir	Pepstatin	Hexa-peptide with unusual amino acid	S*/NM	Antiviral	2003
Vardenafil	Cyclic GMP	Cyclic purine nucleotide	S*/NM	Erectile dysfunction	2003
Rosuvastatin calcium	Mevastatin	Statins	S*/NM	Hypercholesterolaemia	2003
Tadalafil	Cyclic GMP	Cyclic purine nucleotide	S*/NM	Erectile dysfunction	2003
Emtricitabine	Cytidine	Cytidine analogues	S*	HIV	2003
Miglustat; N-butyl-1-deoxynojirimycin	1-deoxynojirimycin	Imino sugars	ND	Gaucher's disease	2003
Co-artemether; Artemether-lumofantrine; Artemether-lumefantrine	Artemisinin	Trioxanes	ND	Antiparasitic	2003
Epipodophyllotoxin	Podophyllotoxin	Podophyllotoxin lignans	ND	Oncological disease	2003
Estradiol; Nomegestrol acetate	Megestrol	Progestogens	ND	Hormone replacement therapy	2003
Isoprenaline	Amphetamine	Phenethylamine class	ND	Cardiovascular disease	2003
Isoprenaline	Ephedrine	Phenethylamine class	ND	Cardiovascular disease	2003
Carbenoxolone; Cbx; Glycyrrhetinic acid hydrogen sucinate	Glycyrrhizinic acid	Triterpenoid saponin glycosides	ND	Immunological, inflammatory and related disease	2003
Drospirenone; Estradiol	Cholesterol	Sterols	ND	Hormone replacement therapy	2003
Drospirenone; Estradiol	Estradiol	Anabolic-androgenic steroids	ND	Hormone replacement therapy	2003
Mycophenolate sodium; Mycophenolic acid	Mycophenolic acid	Mycophenolic acid analogues	N	Immunological, inflammatory and related disease	2003
Daptomycin; A21978c	Daptomycin	13 residue Branched cyclic anionic lipopeptides	N	Antibacterial	2003
Gefitinib	Zeatin	Purine analogues adenine-type	S/NM	Non-small-cell lung cancer	2002
Olmesartan medoxil	Imidazole-5-acetic acid	Imidazole analogues	S/NM	Hypertension	2002
Tiotropium bromide	Atropine	Tropane alkaloids	S*/NM	Chronic obstructive pulmonary disease	2002
Adefovir dipivoxil	Adenosine monophosphate	Purine nucleoside analogues	S*	Chronic hepatitis B	2002
Sodium oxybate	Gamma-Hydroxybutyric acid	Carboxylic acid derivatives	ND	Narcolepsy with episodes of cataplexy	2002
Buprenorphine	Thebaine	Opiate alkaloids	ND	Neurological disease	2002
Treprostinil sodium	Prostacyclin	Prostacyclins	ND	Pulmonary arterial hypertension	2002
Biapenem	Thienamycin	Carbapenam	ND	Antibacterial	2002
Etonogestrel; Ethinylestradiol	Estradiol	Anabolic-androgenic steroids	ND	Contraception	2002
Micafungin sodium; Fk463	FR901379	Echinocandin-like lipopeptides with with	ND	Candida infections in patients undergoing haematopoietic stem cell transplantation	2002

		fatty acid side chain			
Ertapenem sodium	Thienamycin	Carbapenam	ND	Moderate to severe bacterial infection	2002
Eplerenone	Corticosterone	Corticosteroids	ND	Hypertension	2002
Fulvestrant; Ici-182, 780	Estrogen	Anabolic-androgenic steroids	ND	Hormone-receptor-positive metastatic breast cancer	2002
Amrubicin hydrochloride; Sm-5887	Doxorubicin	Anthracyclines	ND	Oncological disease	2002
Artesunate; As	Artemisinin	Trioxanes	ND	Antiparasitic	2002
Fondaparinux sodium	Heparin	Glycosaminoglycans	ND	DVT; anticoagulant	2002
Acetyldigitoxin	Digitoxin	Cardiac glycosides	ND	Cardiovascular disease	2002
Galantamine hydrobromide; Galanthamine	Galantamine	Phenanthrene alkaloids	N	Neurological disease	2002
Morphine sulfate	Morphine	Opiate alkaloids	N	Neurological disease	2002
Tegaserod maleate	Serotonin	Tryptamines	S*/NM	Irritable bowel syndrome	2001
Valganciclovir	Guanosine	Purine nucleoside analogues	S*	Antiviral	2001
Travoprost	Prostaglandin F2alpha	Prostaglandins	ND	Antiglaucoma	2001
Gusperimus trihydrochloride; 15-deoxyspergualin	Spergualin	Peptoid core with guanidylated alkyl group and spermidine-derived polyamine	ND	Immunological, inflammatory and related disease	2001
Methacycline; Metacycline; Methacyclin	Oxytetracycline	Tetracyclines	ND	Antibacterial	2001
Norelgestromin; Ethinylestradiol	Estradiol	Anabolic-androgenic steroids	ND	Contraception	2001
Minocycline hydrochloride; Minocycline	Tetracycline	Tetracyclines	ND	Antibacterial	2001
methyl aminolevulinate	Aminolevulinic acid HCl	Amino acids with acyclic hydroxyl side chain & derivatives	ND	Actinic keratoses	2001
Bimatoprost	Prostaglandin F2alpha	Prostaglandins	ND	Antiglaucoma	2001
Falecalcitrol	Calcitriol	Secosteroids	ND	Sec. hyperthryoidism	2001
Imatinib mesylate; Cgp5714b; Glivec; Sti571	Staurosporine	Staurosporine alkaloids	ND	Anticancer	2001
Pimecrolimus; Sdz-asm-981; Ascomycin	Ascomycin	Macrolide lactones	ND	Immunological, inflammatory and related disease	2001
Dutaseride	Testosterone	Anabolic-androgenic steroids	ND	BPH	2001
Fudosteine	Cysteine	Amino acid with acyclic sulfur-containing side chain	ND	expectorant	2001
Caspofungin acetate	Echinocandin B	Echinocandin-like lipopeptides || Echinocandins	ND	antifungal	2001
Rasburicase	Urate oxidase	EC1.7.3 Oxidoreductases acting on other nitrogenous compounds as donors with oxygen as acceptor	B*	Management of plasma uric acid levels in paediatric patients with leukaemia, lymphoma and solid tumour malignancies	2001
Lopinavir; Abt-378	Pepstatin	Hexa-peptide with unusual amino acid	S*/NM	Antiviral	2000
Verteporfin	Porphyrin	Porphyrins	S*	Photosensitizer	2000

Bulaquine; Chloroquine	Quinine	5-aminoquinolines	S*	Antiparasitic	2000
Drospirenone	Progestin	Progestogens	ND	Contraception	2000
Atosiban	Oxytocin	Vasopressin and oxytocin families	ND	Premature birth	2000
Trimegestone	Promegestone	Steroid derivatives (Norsteroids)	ND	Hormone replacement therapy	2000
Framycetin	Neomycin	Aminoglycosides	ND	Antibacterial	2000
Maxacalcitol	Cholecalciferol	Secosteroids (structurally similar to steroids)	ND	calcium metabolism	2000
Ganirelix acetate	Gonadotropin-Releasing Hormone	Gonadotropin-releasing hormone family peptides	ND	Ovulationi	2000
Gemtuzumab ozogamicin	Calicheamicin	Calicheamicins	ND	Oncological disease	2000
Medroxyprogesterone; Estradiol	17-hydroxyprogesterone	Glucocorticoids	ND	Contraception	2000
Dosmalfate	Diosmin	Flavanone glycosides	ND	Gastroprotectant	2000
Taltirelin	Thyrotropin-Releasing Hormone	Tri-peptide with a imidazole	ND	CNS Stimulant	2000
Egualen sodium	Azulene	Benzenoid polycyclic aromatic hydrocarbons	ND	antiulcer	2000
Arteether; Beta arteether	Artemisinin	Trioxanes	ND	Antiparasitic	2000
Aminolevulinic acid hci	Aminolevulinic acid hci	Amino acids with acyclic hydroxyl side chain & derivatives	N	Actinic keratoses	2000
Digoxin fab	Digoxin	Cardiac glycosides	B	Digoxin toxicity	2000
Telmisartan	Imidazole-5-acetic acid	Imidazole analogues	S/NM	Antihypertensive agent	1999
Levalbuterol hci	Ephedrine	Phenethylamine class	S*/NM	Immunological, inflammatory and related disease - Inflammatory disease	1999
Amprenavir	Pepstatin	Hexa-peptide with unusual amino acid	S*/NM	Antiviral	1999
Abacavir sulfate	Guanosine	Purine nucleoside analogues	S*	Antiviral agent	1999
Nateglinide	D-phenylalanine	α-amino acids	S*	Antidiabetic	1999
Kinetin	Cytokinin	Purine analogues adenine-type	S*	Skin photodamage	1999
Oseltamivir phosphate	N-Acetylneuraminic acid	Sialic acids	NM	Influenza	1999
Zanamivir	N-Acetylneuraminic acid	Sialic acids	NM	antiviral	1999
Valrubicin	Doxorubicin	Anthracyclines	ND	Antineoplastic agent	1999
Dalfopristin; Quinupristin	Pristinamycin IA	Streptogramins	ND	Antibacterial	1999
Dalfopristin; Quinupristin	Pristinamycin IIA	Romidepsin analogues	ND	Antibacterial	1999
Rapacuronium bromide	Dehydroepiandrosterone	Anabolic-androgenic steroids	ND	muscle relaxant	1999
Alitretinoin	Retinoid	Retinoids	ND	Anticancer	1999
Exemestane	Androstenedione	Androgens	ND	Anticancer	1999
Melevodopa; Chf-1301; L-dopa-methyester	L-Dopa	Catecholamines	ND	Neurological disease - AntiParkinsonian	1999
Doxercalciferol	Ergosterol	Sterols	ND	Calcium metabolism	1999
Conjugated estrogens a	Estrogen	Anabolic-androgenic steroids	ND	Hormone replacement therapy	1999

Colforsin daropate hcl	Forskolin	Labdane diterpenes	ND	Cardiovascular and metabolic disease	1999
Sirolimus; Rapamycin	Sirolimus	Macrolides	N	Immunological, inflammatory and related disease	1999
Arglabin	Arglabin	Sesquiterpene lactones	N	Oncological disease	1999
Dermatan sulfate	Dermatan sulfate	Glycosaminoglycans	B*	DVT; anticoagulant	1999
Sildenafil citrate	Cyclic GMP	Cyclic purine nucleotide	S*/NM	Agent for erectile dysfunction	1998
Rizatriptan benzoate	Serotonin	Tryptamines	S*/NM	Agent for migraine Maxalt-MLT	1998
Capecitabine	Uracil	Uracil analogues	S*	Antineoplastic agent	1998
Paricalcitol	Calcitriol	Secosteroids	ND	Agent for secondary hyperparathyroidism	1998
Orlistat; Tetrahydrolipstatin	Lipstatin	Lipstatin analogues	ND	Cardiovascular and metabolic disease	1998
Cefoselis	Cephalosporin	Glycopeptides; Cephalosporins	ND	Antibacterial	1998
Levonorgestrel; Ethinyl estradiol	Cholesterol	Sterols	ND	Contraception	1998
Loteprednol etabonate	Cortisol	Glucocorticoids	ND	Agent for ocular inflammatory conditions	1998
Bemiparin sodium	Heparin	Glycosaminoglycans	ND	DVT; anticoagulant	1998
Miglitol	1-deoxynojirimycin	Imino sugars	ND	Cardiovascular and metabolic disease	1998
Dihydroartemisinin	Artemisinin	Trioxanes	ND	Antiparasitic	1998
Alprostadil	Alprostadil	Prostaglandins	N	Erectile dysfunction	1998
Eprosartan	Imidazole-5-acetic acid	Imidazole analogues	S/NM	Antihypertensive	1997
Irbesartan	Imidazole-5-acetic acid	Imidazole analogues	S/NM	Antihypertensive	1997
Candesartan cilexetil	Imidazole-5-acetic acid	Imidazole analogues	S/NM	Antihypertensive agent	1997
Neflinavir mesylate; Nelfinavir mesylate	Pepstatin	Hexa-peptide with unusual amino acid	S*/NM	Antiviral	1997
Atorvastatin calcium; Atorvastin calcium	Mevastatin	Statins	S*/NM	Cardiovascular disease	1997
Flurithromycin ethylsuccinate	Erythromycin	Macrolides	ND	Antibacterial	1997
Mezlocillin	Penicillin	Penicillins	ND	Antibacterial	1997
Cefcapene pivoxil	Cephalosporin	Glycopeptides; Cephalosporins	ND	Antibacterial	1997
Faropenem sodium; Fropenam	Thienamycin	Carbapenam	ND	Antibacterial	1997
Cefaclor; Cefachlor; Cefaclorum	Cephalosporin	Glycopeptides; Cephalosporins	ND	Antibacterial	1997
Hesperetin	Hesperetin	Flavanones	N	Cardiovascular disease	1997
Ephedrine	Ephedrine	Phenethylamine class	N	Immunological, inflammatory and related disease - Inflammatory disease	1997
Tandospirone	Serotonin	Tryptamines	S/NM	Anxiolytic	1996
Valsartan	Imidazole-5-acetic acid	Imidazole analogues	S/NM	Antihypertensive	1996
Sertindole	Serotonin	Tryptamines	S*/NM	Neuroleptic	1996
Ropinirole hcl	Serotonin	Tryptamines	S*/NM	AntiParkinsonian	1996
Ritonavir	Pepstatin	Hexa-peptide with unusual amino acid	S*/NM	Antiviral	1996
Ramosetron	Serotonin	Tryptamines	S*/NM	Antiemetic	1996
Indinavir sulfate; Idv	Pepstatin	Hexa-peptide with unusual amino acid	S*/NM	Antiviral	1996

Cidofovir	Cytidine monophosphate	Cytidine analogues	S*	Antiviral	1996
Penciclovir	Guanine	Purine analogues guanine-type	S*	Antiviral	1996
Zotarolimus; Abt-578	Sirolimus	Macrolides	ND	Oncological disease	1996
Latanoprost	Prostaglandin F2alpha	Prostaglandins	ND	Antiglaucoma	1996
Oxycodone hcl	Thebaine	Opiate alkaloids	ND	Neurological disease	1996
Oxycodone hcl	Codeine	Opiate alkaloids	ND	Neurological disease	1996
Topotecan hcl	Camptothecin	Camptothecin analogues	ND	Oncological disease	1996
Flutamide	Testosterone	Anabolic-androgenic steroids	ND	Oncological disease	1996
Etoposide phosphate	Podophyllotoxin	Podophyllotoxin lignans	ND	Oncological disease	1996
Zinc hyaluronate	Hyaluronan	Glycosaminoglycans	ND	Vulnerary	1996
Captopril; Sq14225; Capropril	Teprotide	Nonapeptides	ND	Cardiovascular disease	1996
Amodiaquine; Sq	Chloroquine	4-Aminoquinolines	ND	Antiparasitic	1996
Certoparin sodium	Heparin	Glycosaminoglycans	ND	DVT; anticoagulant	1996
Raltitrexed	Folic acid	Folate analogues	ND	cancers	1996
Prezatide copper acetate	Prezatide copper acetate	Tri-peptide with a imidazole	N	Vulnerary	1996
Betain anhydrous	Trimethylglycine	Amino acids with acyclic hydroxyl side chain & derivatives	N	Homocystinuria	1996
Digitoxin	Digitoxin	Cardiac glycosides	N	Cardiovascular disease	1996
Thymalfasin	Thymosin alpha 1	Thymosins (28aa peptides)	B*	Hepatitis	1996
Fosfomycin tromethamine	Fosfomycin tromethamine	Phosphoenolpyruvate analgogues	N	urinary tract infections	1996
Moexipril hci	Teprotide	Nonapeptides	S*/NM	Cardiovascular disease	1995
Spirapril hci	Teprotide	Nonapeptides	S*/NM	Cardiovascular disease	1995
Saquinavir mesylate	Pepstatin	Hexa-peptide with unusual amino acid	S*/NM	Antiviral	1995
Valaciclovir hcl	Guanosine	Purine nucleoside analogues	S*	Antiviral	1995
Gemcitabine hcl	Cytidine	Cytidine analogues	S*	Anticancer	1995
Lamivudine	Zalcitabine	Cytidine analogues	S*	Antiviral	1995
Rimexolone	Cortisol	Glucocorticoids	ND	Inflammatory disease	1995
Docetaxel	Paclitaxel	Taxanes	ND	Oncological disease	1995
Lanreotide	Somatostatin	Somatostatin peptides ; GPCR targeting peptide hormones & analogues (cyclohexapeptides)	ND	Acromegaly	1995
Nalmefene hci	Naltrexone	Opiate alkaloids	ND	Neurological disease	1995
Estrogens; Medroxyproesterone	Estrogen	Anabolic-androgenic steroids	ND	Hormone replacement therapy	1995
Tirilazad mesylate	Cortisol	Glucocorticoids	ND	subarachnoid hemorrhage	1995
Alendronate sodium	2-aminoethylphosphonic acid	Aminodiphosphonates	ND	calcium metabolism	1995
Cefozopran hcl	Cephalosporin	Glycopeptides; Cephalosporins	ND	Antibacterial	1995

Mycophenolate mofetil	Mycophenolic acid	Mycophenolic acid analogues	ND	Immunological, inflammatory and related disease	1995
Carperitide	Carperitide	Natriuretic peptides	N	Congestive heart failure	1995
Tramadol hydrochloride	Tramadol	Small alkaloids with an amine group	N	moderate to moderately severe pain	1995
Losartan potassium	Imidazole-5-acetic acid	Imidazole analogues	S/NM	Antihypertensive	1994
Fluvastatin sodium	Mevastatin	Statins	S*/NM	Cardiovascular disease	1994
Temocapril hydrochloride	Teprotide	Nonapeptides	S*/NM	Cardiovascular disease	1994
Stavudine	Thymidine	Thymidine analogues	S*	Antiviral	1994
Famciclovir	Guanine	Purine analogues guanine-type	S*	Antiviral	1994
Quinagolide hydrochloride	Ergoline	Ergoline alkaloids	NM	antihyperprolactinemia	1994
Rocuronium bromide	Steroid	Steroid derivatives (aminosteroids)	ND	Muscle relaxant	1994
Docarpamine	Dopamine	Catecholamines	ND	Cardiotonic	1994
Zinostatin stimalamer	Neocarzinostatin	Enediynes	ND	Oncological disease	1994
Salmeterol	Adrenaline	Catecholamines	ND	Immunological, inflammatory and related disease - Inflammatory disease	1994
Salmeterol	Ephedrine	Phenethylamine class	ND	Immunological, inflammatory and related disease - Inflammatory disease	1994
Unoprostone isopropyl	Prostaglandin	Prostaglandins	ND	Antiglaucoma	1994
Polaprezinc	Carnosine	Di-peptide with a imidazole	ND	Antiulcer	1994
Trimetrexate glucuronate	Folic acid	Folate analogues	ND	PCP/Toxoplasmosis	1994
Vinorelbine; Vrlb; Navelvine	Vinorelbine	Vinca alkaloids	ND	Oncological disease	1994
Meropenem	Thienamycin	Carbapenam	ND	Antibacterial	1994
Cefditoren pivoxil	Cephalosporin	Glycopeptides; Cephalosporins	ND	Antibacterial	1994
Panipenem; Betamipron	Thienamycin	Carbapenam	ND	Antibacterial	1994
Irinotecan hydrochloride; Cpt-11	Camptothecin	Camptothecin analogues	ND	Oncological disease	1994
Camptothecin; Irinotecan	Camptothecin	Camptothecin analogues	ND	Oncological disease	1994
Betamethasone butyrate propion	Prednisolone	Glucocorticoids	ND	Antiinflammatory	1994
Angiotensin ii	Angiotensin ii	Dipsogen oligopeptides	N	Anticancer	1994
Voglibose; Ao-128	Voglibose	Imino sugars	N	Cardiovascular and metabolic disease	1994
Cysteamine bitartrate	Cysteine	Amino acid with acyclic sulfur-containing side chain	ND	escape the metabolic defect in cystinosis and cystinuria, also used for treatment of radiation sickness	1994
Imidapril hci	Teprotide	Nonapeptides	S*/NM	Cardiovascular disease	1993
Trandolapril	Teprotide	Nonapeptides	S*/NM	Cardiovascular disease	1993
Sorivudine	Thymidine	Thymidine analogues	S*	Antiviral	1993
Gabapentin	GABA	Amino acids with acyclic hydroxyl side chain & derivatives	S*	Antiepileptic	1993
Piperacillin; Tazobactam	Penicillin	Penicillins	ND	Antibacterial	1993
Tretinoin	Vitamin A	Ionone polyisoprene	ND	Antiulcer	1993
Dirithromycin	Erythromycin	Macrolides	ND	Antibacterial	1993

Miltefosine	Phosphatidylcholine	Phospholipids (Phosphatidylcholines)	ND	anticancer	1993
Cefepime	Cephalosporin	Glycopeptides; Cephalosporins	ND	Antibacterial	1993
Cladribine	Purine	Purine	ND	Anticancer	1993
Formestane	Estrogen	Anabolic-androgenic steroids	ND	Anticancer	1993
Ecabet sodium	Abietic acid	Abietane diterpenes	ND	antiulcer	1993
Parnaparin sodium	Heparin	Glycosaminoglycans	ND	DVT; anticoagulant	1993
Cabergoline	Ergot	Ergoline alkaloids	ND	Antihyperprolactinemia	1993
Reviparin sodium	Heparin	Glycosaminoglycans	ND	Anticoagulant	1993
Tacalcitol	Ergosterol	Sterols	ND	Antipsoriatic	1993
Paclitaxel	Paclitaxel	Taxanes	N	Oncological disease	1993
Tacrolimus; Fk-506; Fujimycin	Tacrolimus	Macrolide lactones	N	Immunological, inflammatory and related disease	1993
Calcipotriene	Calcitriol	Secosteroids	ND	psoriasis	1993
Atovaquone	Ubiquinone	Quinone isoprenes	S*	Antiparasitic	1992
Zalcitabine; Ddc; Ddcyd; Dideoxycytidine	Pyrimidine	Pyrimidine analogues	S*	Antiviral	1992
Teniposide; Vm-26	Podophyllotoxin	Podophyllotoxin lignans	ND	Oncological disease	1992
Tazobactam sodium	Penicillin	Penicillins	ND	Antibacterial	1992
Finasteride	Testosterone	Anabolic-androgenic steroids	ND	5 alpha-reductase inhibitor	1992
Danaparoid sodium	Heparin	Glycosaminoglycans	ND	DVT; anticoagulant	1992
Loracarbef	Cephalosporin	Glycopeptides; Cephalosporins	ND	Antibacterial	1992
Iloprost	Prostacyclin PGI2	Prostacyclins	ND	Pulmonary arterial hypertension	1992
Cefetamet pivoxil hcl	Cephalosporin	Glycopeptides; Cephalosporins	ND	Antibacterial	1992
Beraprost sodium	Prostacyclin	Prostacyclins	ND	Platelet aggreg. inhib.	1992
Cefprozil; Cefproxil	Cephalosporin	Glycopeptides; Cephalosporins	ND	Antibacterial	1992
Cefpirome sulfate	Cephalosporin	Glycopeptides; Cephalosporins	ND	Antibacterial	1992
Ceftibuten	Cephalosporin	Glycopeptides; Cephalosporins	ND	Antibacterial	1992
Sapropterin dihydrochloride	Sapropterin	Purine analogues	ND	Hyperphenylalaninaemia	1992
Andrographolide	Andrographolide	Labdane diterpenes	N	Antibacterial - Chemotherapy: Bacillary dysentery	1992
Pentostatin; Deoxycoformycin	Pentostatin	Purine nucleoside analogues	N	Oncological disease	1992
Masoprocol	Masoprocol	Catechols & derivatives	N	Oncological disease	1992
Aldesleukin	IL2	Interleukins	B*	Anticancer	1992
Teceleukin	IL2	Interleukins	B*	Anticancer	1992
Celmoleukin	IL2 + mAb	Interleukins	B*	Anticancer	1992
Fosinopril sodium	Teprotide	Nonapeptides	S*/NM	Cardiovascular disease	1991
Sumatriptan succinate	Serotonin	Tryptamines	S*/NM	Antimigraine	1991
Didanosine; 2',3'-dideoxyinosine; Ddi	Purine	Purine	S*	Antiviral	1991

Fludarabine phosphate	Adenosine monophosphate	Purine nucleoside analogues	S*	Anticancer	1991
Halobetasol propionate	Glucocorticoid	Glucocorticoids	ND	Antiinflammatory	1991
Cefotiam hexetil hydrochloride	Cephalosporin	Glycopeptides; Cephalosporins	ND	Antibacterial	1991
Nitisinone; Ntbc	Leptospermone	β-triketones	ND	Hereditary tyrosinaemia type 1	1991
Cefdinir	Cephalosporin	Glycopeptides; Cephalosporins	ND	Antibacterial agent	1991
Arteflene; Ro 42-1611	Yingzhaosu A	Sesquiterpene Peroxides	ND	Antiparasitic	1991
Calcipotriol	Calcitriol	Secosteroids	ND	Antipsoriatic	1991
Oxacillin sodium	Penicillin	Penicillins	ND	Antibacterial	1991
Argatroban	L-arginine		S*/NM	Antithrombotic	1990
Salmeterol xinafoate	Ephedrine	Phenethylamine class	S*/NM	Immunological, inflammatory and related disease - Inflammatory disease	1990
Remoxipride hydrochloride	Dopamine	Catecholamines	S*/NM	Antipsychotic	1990
Cilazapril	Teprotide	Nonapeptides	S*/NM	Cardiovascular disease	1990
Benazepril hydrochloride	Teprotide	Nonapeptides	S*/NM	Cardiovascular disease	1990
Idarubicin hydrochloride	Daunorubicin	Anthracyclines	ND	Oncological disease	1990
Eflornithine hcl	Ornithine	Amino acids with acyclic hydroxyl side chain & derivatives	ND	antiparasitic	1990
Cefodizime sodium	Cephalosporin	Glycopeptides; Cephalosporins	ND	Antibacterial	1990
Clarithromycin	Erythromycin	Macrolides	ND	Antibacterial	1990
Arbekacin	Kanamycin	Aminoglycosides	ND	Antibacterial	1990
Fluticasone propionate	Fluticasone	Glucocorticoids	ND	Inflammatory disease	1990
Ethyl icosapentate	Eicosapentaenoic acid	Leukotrienes	ND	Antithrombotic	1990
Pumactant	Monocrotaline	Pyrrolizidine alkaloids	ND	respiratory distress syndrome	1990
Capsaicin; Algrx 4975	Capsaicin	Capsaicinoids	N	Neurological disease	1990
Acarbose	Acarbose	Acarviosin maltose	N	Cardiovascular and metabolic disease	1990
Ramipril	Teprotide	Nonapeptides	S*/NM	Cardiovascular disease	1989
Droxidopa	L-serine	Amino acids with acyclic hydroxyl side chain & derivatives	S*/NM	AntiParkinsonian	1989
Dopexamine	Dopamine	Catecholamines	S*/NM	Cardiotonic	1989
Delapril	Teprotide	Nonapeptides	S*/NM	Cardiovascular disease	1989
Quinapril	Teprotide	Nonapeptides	S*/NM	Cardiovascular disease	1989
Ethanolamine oleate	Oleic acid	Monounsaturated omega-9 fatty acids	S*	Sclerosant	1989
Acamprosate calcium	GABA	Amino acids with acyclic hydroxyl side chain & derivatives	S*	Alcohol dependence	1989
Cefmetazole sodium	Cephamycin nucleus	Cephalosporins	ND	Antibacterial	1989
Cefpodoxime proxetil	Cephalosporin	Glycopeptides; Cephalosporins	ND	Antibacterial	1989
Tiopronin	Glycine	Amino acids with acyclic hydroxyl side chain &	ND	Urolithiasis	1989

		derivatives			
Deslorelin	Gonadotropin-releasing hormone (triptorelin)	Gonadotropin-releasing hormone family decapeptides	ND	Oncological disease	1989
Lmw heparin	Heparin	Glycosaminoglycans	ND	Anticoagulant	1989
Ipriflavone	Isoflavone	Isoflavones	ND	Calcium metabolism	1989
Acitretin	Retinoid	Retinoids	ND	Antipsoriatic	1989
Rv-11	Erythromycin	Macrolides	N	Antibacterial	1989
Pravastatin	Mevastatin	Statins	N	Cardiovascular disease	1989
Solamargine	Solamargine	Solasodine glycoalkaloids	N	Oncological disease	1989
Adenosine	Adenosine	Purine nucleoside analogues	N	Antiarrhythmic	1989
Interleukin-2	Interleukin-2	Interleukins	B*	Anticancer	1989
Denopamine	Dopamine	Catecholamines	S*/NM	Cardiotonic	1988
Alacepril	Teprotide	Nonapeptides	S*/NM	Cardiovascular disease	1988
Perindopril	Teprotide	Nonapeptides	S*/NM	Cardiovascular disease	1988
Epervudine	Deoxyuridine	Uridine analogues	S*	Antiviral	1988
Ganciclovir	Guanosine	Purine nucleoside analogues	S*	Antiviral	1988
Azithromycin	Erythromycin	Macrolides	ND	Antibacterial	1988
Octreotide	Somatostatin	Somatostatin peptides ; GPCR targeting peptide hormones & analogues (cyclohexapeptides)	ND	Antisecretory	1988
Hydrocortisone aceponate	Hydrocortisone	Glucocorticoids	ND	Antiinflammatory	1988
Pirarubicin	Doxorubicin	Anthracyclines	ND	Oncological disease	1988
Pergolide mesylate	Ergoline	Ergoline alkaloids	ND	AntiParkinsonian	1988
Secalciferol	Cholecalciferol	Secosteroids (structurally similar to steroids)	ND	calcium metabolism	1988
Limaprost	Alprostadil	Prostaglandins	ND	Antithrombotic	1988
Mifepristone	Glucocorticoid	Glucocorticoids	ND	Abortifacient	1988
Tibolone	Tibolone	Anabolic steroids	ND	Anabolic metabolism	1988
Isepamicin	Gentamicin	Aminoglycosides	ND	Antibacterial	1988
Propentofylline propionate	Xanthine	Purine analogues xanthine-type	ND	Vasodilator; cerebral	1988
Halopredone acetate	Glucocorticoid	Glucocorticoids	ND	Antiarthritic	1988
Flomoxef sodium	Cephalosporin	Glycopeptides; Cephalosporins	ND	Antibacterial	1988
Flutropium bromide	Atropine	Tropane alkaloids	ND	Antitussive	1988
Erythromycin acistrate; Cas 96128-89-1	Erythromycin	Macrolides	ND	Antibacterial	1988
Choline alfoscerate	Choline alfoscerate	Choline alfoscerate	ND	Nootropic	1988
Carumonam	Aztreonam	Penicillins	ND	Antibacterial	1988
Simvastatin; Simvastin	Mevastatin	Statins	N	Cardiovascular disease	1988
Midecamycin	Midecamycin	Macrolides	N	Antibiotic	1988
Fosfomycin trometamol	Fosfomycin	Phosphoenolpyruvate	N	Antibacterial	1988

	trometamol	analgogues			
Josamycin	Josamycin	Macrolides	N	Antibacterial	1988
Gamolenic acid	Gamolenic acid	Leukotrienes	N	Antiallergic	1988
Enalaprilat	Teprotide	Nonapeptides	S*/NM	Cardiovascular disease	1987
Lisinopril	Teprotide	Nonapeptides	S*/NM	Cardiovascular disease	1987
Eptazocine hbr	Morphine	Opiate alkaloids	S*	Analgesic	1987
Zidovudine; Zdv; Azt; Azidothymidine; 3'-azido-3'-deoxythymidine	Thymidine	Thymidine analogues	S*	Antiviral	1987
Doxifluridine	Uridine	Uridine analogues	S*	Anticancer	1987
Goserelin	Gonadotropin-releasing hormone	Gonadotropin-releasing hormone family peptides	ND	Oncological disease	1987
Hidrosmin	Dicoumarol	4-Hydroxycoumarins	ND	Cardiovascular disease	1987
Lenampicillin hci	Penicillin	Penicillins	ND	Antibacterial	1987
Sultamicillin tosilate	Penicillin	Penicillins	ND	Antibacterial	1987
Cefteram pivoxil	Cephalosporin	Glycopeptides; Cephalosporins	ND	Antibacterial	1987
Cefixime	Cephalosporin	Glycopeptides; Cephalosporins	ND	Antibacterial	1987
Roxithromycin	Erythromycin	Macrolides	ND	Antibacterial	1987
Cefminox sodium	Cephalosporin	Glycopeptides; Cephalosporins	ND	Antibacterial	1987
Enoxaparin	Heparin	Glycosaminoglycans	ND	DVT; anticoagulant	1987
Metergolin	Ergoline	Ergoline alkaloids	ND	Antihyperprolactinemia	1987
Gestodene; Ethinylestradiol	Estradiol	Anabolic-androgenic steroids	ND	Contraception	1987
Mometasone furoate	Mometasone	Glucocorticoids	ND	Antiinflammatory	1987
Aspoxicillin	Penicillin	Penicillins	ND	Antibacterial	1987
Cefuzonam sodium	Cephalosporin	Glycopeptides; Cephalosporins	ND	Antibacterial	1987
Cefuroxime axetil	Cephalosporin	Glycopeptides; Cephalosporins	ND	Antibacterial	1987
Artemether	Artemisinin	Trioxanes	ND	Antiparasitic	1987
Ornoprostil	Alprostadil	Prostaglandins	ND	Antiulcer	1987
Cefpimizole	Cephalosporin	Glycopeptides; Cephalosporins	ND	Antibacterial	1987
Ganglioside gm1	Ganglioside gm1	Gangliosides	N	Antithrombotic	1987
Ivermectin	Ivermectin	Avermectins	N	Antiparasitic	1987
Bucillamine	Penicillin	Penicillins	N	Immunomodulator	1987
Lovastatin; Mevinolin	Lovastatin	Statins	N	Cardiovascular disease	1987
Plaunotol	Plaunotol	Acyclic diterpene	N	Antiulcer	1987
Ubenimex	Ubenimex	Phenethylamine class	N	Immunological, inflammatory and related disease - Immunostimulant	1987
Artemisinin; Qinghaosu	Artemisinin	Trioxanes	N	Antiparasitic	1987
Hyaluronate sodium	Hyaluronate sodium	Glycosaminoglycans	B*	Joint lubricant	1987
Ursodiol	Ursodiol	Steroid derivatives (steroid acids)	N	metabolic byproducts of intestinal bacteria	1987
Cefmenoxime hydrochloride	Cephalosporin	β-lactams	ND	antibiotic	1987

Ticarcillin; Clavulanate	Penicillin	Penicillins	ND	Antibacterial	1986
Sulbactam sodium	Penicillin	Penicillins	ND	Beta-lactamase inhibitor	1986
Brovincamine fumarate	Vincamine	Monoterpenoid indole alkaloids	ND	Vasodilator, cerebral	1986
Rokitamycin	Midecamycin	Macrolides	ND	Antibacterial	1986
Levacecarnine hci	Lysine	Amino acids with acyclic hydroxyl side chain & derivatives	ND	Nootropic	1986
Nomegestrol acetate	Megestrol	Progestogens	ND	Progestogen	1986
Triptorelin	Gonadotropin-relea sing hormone	Gonadotropin-releasing hormone family peptides	ND	Oncological disease	1986
Gestrinone	Estrogen	Anabolic-androgenic steroids	ND	Antiprogestogenic	1986
Prednicarbate	Prednisolone	Glucocorticoids	ND	Inflammatory disease	1986
Deflazacort	Glucocorticoid	Glucocorticoids	ND	Antiinflammatory	1986
Gonadoreline-6-d-trp acetate	Gonadotropin-relea sing hormone	Gonadotropin-releasing hormone family peptides	ND	Oncological disease	1986
Norgestimate	Norgestrel	Progestogens	ND	Progestogen	1986
Idebenone	Coenzyme Q10	Quinone isoprenes	ND	nootropic	1986
Schizophyllan	Schizophyllan	β-Glucans	N	Immunological, inflammatory and related disease - Immunostimulant	1986
Thc; Dronabinol; Delta9-tetrahydrocannabinol	Tetrahydrocannabin ol	Cannabinols	N	Neurological disease	1986
Lentinan	Lentinan	β-Glucans	N	Oncological disease	1986
Mupirocin	Mupirocin	Polyketide-derived acids with a long tail	N	Antibacterial	1986
Tranexamic acid	Lysine	Amino acids with acyclic hydroxyl side chain & derivatives	ND	treat or prevent excessive blood loss during surgery and in various other medical conditions	1986
Progabide	Gamma-aminobutyr ic acid (GABA)	Amino acids with acyclic hydroxyl side chain & derivatives	S*	Anticonvulsant	1985
Imipenem; Cilastatin	Thienamycin	Carbapenam	ND	Antibacterial	1985
Alclometasone dipropionate	Methylprednisolone	Glucocorticoids	ND	Inflammatory disease	1985
Cefbuperazone sodium	Cephalosporin	Glycopeptides; Cephalosporins	ND	Antibacterial	1985
Cefpiramide sodium	Cephalosporin	Glycopeptides; Cephalosporins	ND	Antibacterial	1985
Rosaprostol	Prostaglandin E	Prostaglandins	ND	Antiulcer	1985
Nabumetone	Indole-3-acetic acid	Auxins	ND	antiinflammatory	1985
Imipenem	Thienamycin	Carbapenam	ND	Antibacterial	1985
Beta-acetyldigoxin	Digoxin	Cardiac glycosides	ND	Cardiovascular disease	1985
Haem arginate	Heme	Porphyrins	ND	porphyria	1985
Cefoxitin sodium	Cephamycin nucleus	Cephalosporins	ND	Antibacterial	1985
Mefloquine hci	Quinine	5-aminoquinolines	ND	Antiparasitic	1985
Enprostil	Prostaglandin E2	Prostaglandins	ND	Antiulcer	1985
Nadroparin calcium	Heparin	Glycosaminoglycans	ND	Anticoagulant	1985
Dalteparin sodium	Heparin	Glycosaminoglycans	ND	DVT; anticoagulant	1985
Misoprostol	Alprostadil	Prostaglandins	ND	Antiulcer	1985

Doxofylline	Theophylline	Purine analogues xanthine-type	ND	Bronchodilator	1985
Cimetropium bromide	Scopolamine	Tropane alkaloids	ND	Antispasmodic	1985
Miokamycin; Miocamycin; Mom	Midecamycin	Macrolides	N	Antibacterial	1985
Astromycin sulfate	Astromycin sulfate	Aminoglycosides	N	antibacterial	1985
Podophyllotoxin; Podofilox	Podophyllotoxin	Podophyllotoxin lignans	N	Antiviral	1985
Amiodarone hydrochloride	Khellin	Furanochromones	ND	antiarrhythmic agent	1985
Levocarnitine	Lysine	Amino acids with acyclic hydroxyl side chain & derivatives	ND	nutritional supplement	1985
Levocarnitine	Methionine	Amino acid with acyclic sulfur-containing side chain	ND	nutritional supplement	1985
Enalapril maleate	Teprotide	Nonapeptides	S*/NM	Cardiovascular disease	1984
Mitoxantrone hci	Doxorubicin	Anthracyclines	S*	Oncological disease	1984
Fosfosal	Salicylic acid	Monohydroxybenzoic acids	S*	Analgesic	1984
Nafcillin	Penicillin	Penicillins	ND	Antibacterial	1984
Temocillin disodium	Penicillin	Penicillins	ND	Antibacterial	1984
Buserelin acetate	Gonadotropin-relea sing hormone	Gonadotropin-releasing hormone family peptides	ND	Oncological disease	1984
Sofalcone	Sophoradin	Chalconoids	ND	Antiulcer	1984
Nicorandil	Niacinamide	Pyridine alkaloids	ND	Vasodilator; coronary	1984
Teprenone	Coenzyme Q10	Quinone isoprenes	ND	Antiulcer	1984
Naltrexone hci	Thebaine	Opiate alkaloids	ND	Neurological disease	1984
Ceforanide	Cephalosporin	Glycopeptides; Cephalosporins	ND	Antibacterial	1984
Cefonicid sodium; Cefonicide; Cefonicid	Cephalosporin	Glycopeptides; Cephalosporins	ND	Antibacterial	1984
Ibopamine hci	Dopamine	Catecholamines	ND	Cardiotonic	1984
Epirubicin hci	Doxorubicin	Anthracyclines	ND	Oncological disease	1984
Cefotetan	Cephamycin nucleus	Cephalosporins	ND	Antibacterial	1984
Leuprolide acetate	Gonadotropin-relea sing hormone	Gonadotropin-releasing hormone family peptides	ND	Oncological disease	1984
Amoxicillin; Clavulanic acid	Penicillin	Penicillins	ND	Antibacterial	1984
Melinamide	Linoleic acid	Unsaturated long chain fatty acid analogues	ND	hypocholesterolemic	1984
Nicotine	Nicotine	Nicotine alkaloids	N	Insecticide	1984
Papaverine	Papaverine	Benzylisoquinoline alkaloids	N	Neurological disease	1984
Mizoribine	Mizoribine	Imidazole nucleosides	N	Immunological, inflammatory and related disease	1984
Pentoxifylline	Xanthine	Purine analogues xanthine-type	ND	intermittent claudication resulting from obstructed arteries in the limbs, and vascular dementia	1984
Betaxolol hci	Isoprenaline	Catecholamines	S*/NM	Cardiovascular disease	1983
Enocitabine	Cytidine	Cytidine analogues	S*	Anticancer	1983
Celiprolol hcl	Epinephrine	Catecholamines	S*	Antihypertensive	1983

Befunolol hci	Epinephrine	Catecholamines	S*	Antiglaucoma	1983
Promegestone	Progestin	Progestogens	ND	Progestogen	1983
Halometasone	Betamethasone	Glucocorticoids	ND	Inflammatory disease	1983
Hydrocortisone butyrate	Hydrocortisone	Glucocorticoids	ND	Inflammatory disease	1983
Oxitropium bromide	Scopolamine	Tropane alkaloids	ND	Bronchodilator	1983
Epoprostenol sodium	Epoprostenol sodium	Prostacyclins	ND	Platelet aggreg. inhib.	1983
Gemeprost	Alprostadil	Prostaglandins	ND	Abortifacient	1983
Ceftazidime pentahydrate	Cephalosporin	Glycopeptides; Cephalosporins	ND	Antibacterial	1983
Elliptinium acetate	Ellipticine	Ellipticine alkaloids	ND	Oncological disease	1983
Sodium cellulose po4	Cellulose	Polysaccharides	ND	hypocalciuric	1983
Ciclosporin; Cyclosporine; Cyclosporin; Ciclosporin a; Cyclosporine a	Cyclosporine	Nonribosomal peptide cyclic structure	N	Immunological, inflammatory and related disease	1983
(+)-catechin	(+)-catechin	Catechins	N	Haemostatic	1983
Restasis	Ciclosporin	Nonribosomal peptide cyclic structure	N	Keratoconjunctivitis sicca (chronic dry eye disease)	1983
Chenodiol	Chenodiol	Steroid derivatives (steroid acids)	N	Anticholelithogenic	1983
Moxalactam disodium; Latamoxef	Cephamycin nucleus	Cephalosporins	ND	Antibacterial	1982
Ceftriaxone sodium	Cephalosporin	Glycopeptides; Cephalosporins	ND	Antibacterial	1982
Dextromethorphan; Dxm; Dm	Codeine	Opiate alkaloids	ND	Neurological disease - Antitussive	1982
Vecuronium bromide	Steroid	Steroid derivatives (aminosteroids)	ND	Muscle relaxant	1982
Apalcillin sodium	Penicillin	Penicillins	ND	Antibacterial	1982
Ceftizoxime sodium	Cephalosporin	Glycopeptides; Cephalosporins	ND	Antibacterial	1982
Lisuride maleate	Ergot	Ergoline alkaloids	ND	Antihyperprolactinemia	1982
Nabilone	Tetrahydrocannabin ol	Cannabinols	ND	Neurological disease	1982
Micronomicin sulfate	Micronomicin sulfate	Aminoglycosides	N	Antibacterial	1982
Extract of serenoa repens	β-Sitosterol	Sterols	N	Benign prostatic hypertrophy	1982
Chloramphenicol	Chloramphenicol	Chloramphenicol alkaloids	N	Antibacterial	1982
Isotretinoin; Isotrentoin	Vitamin A	Ionone polyisoprene	N	Antiacne	1982
Indoramin hcl	Tryptophol	Tryptamines	S*	Antihypertensive	1981
Carmofur	Uracil	Uracil analogues	S*	Anticancer	1981
Inosine pranobex	Inosine	Purine nucleoside analogues	S*	Antiviral	1981
Salbutamol; Albuterol	Adrenaline	Catecholamines	ND	Immunological, inflammatory and related disease - Inflammatory disease	1981
Salbutamol; Albuterol	Ephedrine	Phenethylamine class	ND	Immunological, inflammatory and related disease - Inflammatory disease	1981
Atenolol	Isoprenaline	Catecholamines	ND	Cardiovascular disease	1981
Budesonide	Glucocorticoid	Glucocorticoids	ND	Antiasthmatic	1981
Cefotiam hcl	Cephalosporin	Glycopeptides; Cephalosporins	ND	Antibacterial	1981

Sulprostone	Dinoprostone	Prostaglandins	ND	Abortifacient	1981
Desogestrel; Ethinylestradiol	Estradiol	Anabolic-androgenic steroids	ND	Contraception	1981
Cefamandole nafate; Cephamandole	Cephalosporin	Glycopeptides; Cephalosporins	ND	Antibacterial	1981
Ceftiofur	Cephalosporin	Glycopeptides; Cephalosporins	ND	Antibacterial	1981
Cefotaxime sodium	Cephalosporin	Glycopeptides; Cephalosporins	ND	Antibacterial	1981
Cefoperazone sodium	Cephalosporin	Glycopeptides; Cephalosporins	ND	Antibacterial	1981
Amikacin	Kanamycin	Aminoglycosides	ND	Antibacterial	1981
Peplomycin	Bleomycin	Peptide-polyketides	N	Oncological disease	1981
Paromomycin sulfate	Paromomycin sulfate	Aminoglycosides	N	Antibacterial	1981
Aclarubicin	Aclarubicin	Anthracyclines	N	Oncological disease	1981
Toposar; Vp-16	Podophyllotoxin	Podophyllotoxin lignans	ND	Oncological disease	1980
Ribavirin	Showdomycin	Imidazole ribosides	ND	Antiviral	1980
Cefsulodin	Cephalosporin	Glycopeptides; Cephalosporins	ND	Antibiotic	1980
Terbutyline	Epinephrine	Catecholamines	ND	Antihypertensive	1980
Estramustine	Cholesterol	Sterols	ND	Antineoplastic	1980
Estramustine	Estradiol	Anabolic-androgenic steroids	ND	Antineoplastic	1980
Cefadroxil; Cefadroxil hemihydrate	Cephalosporin	Glycopeptides; Cephalosporins	ND	Antibacterial	1980
Alpha-acetyldigoxin	Digoxin	Cardiac glycosides	ND	Cardiovascular disease	1980
Yohimbine	Yohimbine	Yohimbine alkaloids	N	Aphrodisiac	1980
Xanthotoxin; Ammoidin; 8-methoxypsoralen	Methoxsalen	Furanocoumarins	N	Leukoderma, Vitiligo	1980
Vasicine; Peganine	Vasicine	Quinazoline alkaloids	N	Oxytocic	1980
Theobromine	Theobromine	Purine analogues	N	Vasodilator, Diuretic	1980
Stevioside	Stevioside	Steviol glycosides	N	Sweetener	1980
Silymarin	Silymarin	Flavanones	N	Antihepatotoxic	1980
Santonin	Santonin	Sesquiterpene lactones	N	Anthelmintic - Ascaricide	1980
Sanguinarine	Sanguinarine	Benzylisoquinoline alkaloids	N	Dental plaque inhibitor	1980
Huperzine; Huperzine a	Huperzine a	Sesquiterpene alkaloids	N	Neurological disease	1980
Nalbuphine	Thebaine	Opiate alkaloids	ND	Neurological disease	1979
Cyclacillin	Penicillanic acid	Penicillins	ND	Antibiotic	1979
Vindesine; Vds	Vinblastine	Vinca alkaloids	ND	Oncological disease	1979
Scopolamine	Scopolamine	Tropane alkaloids	N	Immunological, inflammatory and related disease - Sedative	1979
Gentamicin	Gentamicin	Aminoglycosides	N	Antibacterial	1979
Metoprolol tartrate	Isoprenaline	Catecholamines	ND	Cardiovascular disease	1978
Natamycin; Pimaricin	Natamycin	Macrolides	N	Antifungal	1978
Rutin	Rutin	Flavonol rutinose	N	Capillary fragility	1978
Rotenone	Rotenone	Rotenoids	N	Piscicide	1978

Rorifone	Rorifone	Rorifone analogues	N	Antitussive	1978
Picrotoxin	Picrotoxin	Morphinans	N	Analeptic	1978
Palmatine; Filiraurine	Palmatine	Protoberberine alkaloids	N	Antipyretic, Detoxicant	1978
Pachycarpine; (+)-sparteine	Pachycarpine	Lupin alkaloid containing a tetracyclic bis-quinolizidine ring system	N	Oxytocic	1978
Nordihydroguaiaretic acid	Nordihydroguaiaretic acid	Catechols & derivatives	N	Antioxidant	1978
Menthol	Menthol	Monocyclic monoterpenes	N	Rubefacient	1978
Khellin	Khellin	Furanochromones	N	Bronchodilator	1978
Hyoscyamine	Hyoscyamine	Tropane alkaloids	N	Anticholinergic	1978
Hydrastine	Hydrastine	Phthalideisoquinoline alkaloids	N	Haemostatic, Astringent	1978
Hesperidin	Hesperidin	Flavanone glycosides	N	Capillary fragility	1978
Glycyrrhizin; Glycyrrhetic acid	Glycyrrhizinic acid	Triterpenoid saponin glycosides	N	Sweetener, Addison's disease	1978
Glaziovine; Glasiovine	Glaziovine	Proaporphine alkaloids	N	Antidepressant	1978
Glaucine	Glaucine	Isoquinoline alkaloids	N	Antitussive	1978
Danthron; 1,8-dihydroxy-anthraquinone	Danthron	Anthraquinones	N	Laxative	1978
Cynarin	Cynarin	Hydroxycinnamates	N	Choleretic	1978
Camphor	Camphor	Cyclic ketones of the hydroaromatic terpene group	N	Rubefacient	1978
Asiaticoside	Asiaticoside	Triterpenoid glycosides	N	Vulnerary	1978
Arecoline	Arecoline	Arecoline alkaloids	N	Anthelmintic - Chemotherapy	1978
Anisodine	Anisodine	Tropane alkaloids	N	Anticholinergic	1978
Anisodamine	Anisodamine	Tropane alkaloids	N	Anticholinergic	1978
Allyl isothiocyanate	Allyl isothiocyanate	Isothiocyanates	N	Rubefacient	1978
Allantoin	Allantoin	Diazole urea	N	Vulnerary	1978
Ajmalicine	Ajmalicine	Yohimbine alkaloids	N	Circulatory disorders	1978
Agrimophol	Agrimophol	Phloroglucinol derivatives	N	Anthelmintic - Chemotherapy	1978
Vincamine	Vincamine	Monoterpenoid indole alkaloids	N	Cerebral stimulant	1978
Strychnine	Strychnine	Strychnine alkaloids	N	Neurological disease	1978
Thymol	Thymol	Monoterpene phenols	N	Antifungal - Chemotherapy: Antifungal	1978
Neoandrographolide	Neoandrographolide	Diterpene lactones	N	Antibacterial - Chemotherapy: Bacillary dysentery	1978
Berberine	Berberine	Protoberberine alkaloids	N	Antibacterial - Chemotherapy: Bacillary dysentery	1978
Salicin	Salicin	Benzyl alcohol β-glucosides	N	Immunological, inflammatory and related disease - Analgesic	1978
Aescin	Aescin	Saponins	N	Immunological, inflammatory and related disease - Inflammatory disease	1978
Rescinnamine	Rescinnamine	Rauwolfia alkaloids	N	Cardiovascular disease	1978
Kawain	Kawain	Chalconoids	N	Cardiovascular disease	1978
Lanatoside c	Lanatoside c	Cardiac glycosides	N	Cardiovascular disease	1978

Lanatoside b	Lanatoside b	Cardiac glycosides	N	Cardiovascular disease	1978
Lanatoside a	Lanatoside a	Cardiac glycosides	N	Cardiovascular disease	1978
Digitalin	Digitalin	Cardiac glycosides	N	Cardiovascular disease	1978
Deslanoside	Deslanoside	Cardiac glycosides	N	Cardiovascular disease	1978
Convallatoxin	Convallatoxin	Cardiac glycosides	N	Cardiovascular disease	1978
Ouabain; G-strophanthin	Ouabain	Cardiac glycosides	N	Cardiovascular disease	1978
Sennoside b	Sennoside b	Anthraquinones	N	Cardiovascular disease	1978
Sennoside a	Sennoside a	Anthraquinones	N	Cardiovascular disease	1978
Demecolcine	Demecolcine	Colchicine alkaloids	N	Oncological disease - Chemotherapy: Antitumor agent	1978
Colchiceine amide	Colchiceine amide	Colchicine alkaloids	N	Oncological disease - Chemotherapy: Antitumor agent	1978
Monocrotaline	Monocrotaline	Pyrrolizidine alkaloids	N	Oncological disease - Antitumor agent	1978
(+-)-tetrahydropalmatine; Rotundine	Rotundine	Isoquinoline alkaloids	N	Immunological, inflammatory and related disease - Analgesic; Sedative; Tranquillizer	1978
Calcitriol	Calcitriol	Secosteroids	N	increasing the uptake of calcium from the gut into the blood, and (2) possibly increasing the release of calcium into the blood from bone	1978
Bromocriptine mesylate	Ergoline	Ergoline alkaloids	ND	pituitary tumors, Parkinson's disease (PD), hyperprolactinaemia, neuroleptic malignant syndrome, and type 2 diabetes	1978
Azlocillin	Penicillin	Penicillins	ND	Antibacterial	1977
Ipratropium; Ipratropium bromide	Tiotropium	Tiotropium alkaloids	ND	Anticholinergic	1977
Dinoprostone	Prostaglandin E2	Prostaglandins	N	vaginal suppository	1977
Dextroamphetamine sulfate	Amphetamine	Phenethylamine class	ND	ADHD; narcolepsy	1976
Cefalexin; Cephalexin	Cephalosporin	Glycopeptides; Cephalosporins	ND	Antibacterial	1976
Theophylline	Theophylline	Purine analogues xanthine-type	N	respiratory diseases	1976
Candicidin	Candicidin	Heptaenic macrolides	N	Antifungal	1976
Neocarzinostatin	Neocarzinostatin	Enediynes	N	Oncological disease	1976
VIDARABINE	Spongouridine	Uridine analogues	S*	Antiviral	1976
VIDARABINE	Spongothymidine	Cytosine analog (with an arabinose sugar)	S*	Antiviral	1976
Bacampicillin	Penicillin	Penicillins	ND	Antibacterial	1975
Tobramycin sulfate	Tobramycin sulfate	Aminoglycosides	N	Antibacterial	1975
Betamethasone dipropionate	Betamethasone dipropionate	Glucocorticoids	N	Anti-inflammatory	1975
Pseudoephedrine	Pseudoephedrine	Phenethylamine class	N	Immunological, inflammatory and related disease - Sympathomimetic	1975
Triptolide	Triptolide	Diterpenoid epoxide	N	Immunological, inflammatory and related disease	1975
Eupatilin	Eupatilin	O-methylated flavones	N	Immunological, inflammatory and related disease	1975
Digoxin; Digitalis	Digoxin	Cardiac glycosides	N	Cardiovascular disease	1975
Cefradine; Cephradine	Cephalosporin	Glycopeptides; Cephalosporins	ND	Antibacterial	1974
Cloxacillin; Cloxacillin sodium	Penicillin	Penicillins	ND	Antibacterial	1974

Cefapirin; Cephapirin	Cephalosporin	Glycopeptides; Cephalosporins	ND	Antibacterial	1974
Methyltestosterone	Testosterone	Anabolic-androgenic steroids	ND	Oncological disease	1974
Amoxicillin; Amoxycillin	Penicillin	Penicillins	ND	Antibacterial	1974
Betamethasone sodium phosphate	Betamethasone sodium phosphate	Glucocorticoids	N	Anti-inflammatory	1974
Dopamine hydrochloride	Dopamine hydrochloride	Catecholamines	N	Neurological disease	1974
L-dopa	L-Dopa	Catecholamines	N	Neurological disease - AntiParkinsonism	1974
Calusterone	Methyltestosterone	Anabolic-androgenic steroids	ND	Oncological disease	1973
Acetylcholine chloride	Acetylcholine chloride	Choline acetic acid	N	Neurological disease	1973
Kanamycin	Kanamycin	Aminoglycosides	N	Antibacterial	1973
Atropine	Atropine	Tropane alkaloids	N	Anticholinergic - For the treatment of poisoning by susceptible organophosphorous nerve agents having cholinesterase activity as well as organophosphorous or carbamate insecticides.	1973
Fosfestrol	Estrogen	Anabolic-androgenic steroids	ND	Anticancer	1972
Carbenicillin	Penicillin	Penicillins	ND	Antibacterial	1972
Carindacillin; Carbenicillin indanyl	Penicillin	Penicillins	ND	Antibacterial	1972
Pristinamycin; Pristinamycine	Pristinamycin	Streptogramins	N	Antibacterial	1972
Heparin sodium	Heparin	Glycosaminoglycans	N	Anticoagulant	1972
Floxuridine	Arabinosyl nucleosides	Uracil analogues	S*	Oncological disease	1971
Megesterol acetate	Progresterone	Progestogens	ND	Anticancer	1971
Naloxone; Naltrexate	Thebaine	Opiate alkaloids	ND	Neurological disease	1971
Megestrol acetate	Megestrol	Progestogens	ND	Oncological disease	1971
Dicloxacillin; Dicoxacillin	Penicillin	Penicillins	ND	Antibacterial	1971
Cefazolin; Cefazoline; Cephazolin	Cephalosporin	Glycopeptides; Cephalosporins	ND	Antibacterial	1971
Capreomycin	Capreomycin	Tuberactinomycin family	N	Antibacterial	1971
Amphetamine	Amphetamine	Phenethylamine class	N	Attention-deficit disorder	1971
Amphetamine	Ephedrine	Phenethylamine class	N	Attention-deficit disorder	1971
Testolactone	Progesterone	Progestogens	ND	Oncological disease	1970
Prednisolone	Cortisol	Glucocorticoids	ND	Oncological disease	1970
Penicillamine	Penicillin	Penicillins	ND	Antibiotic	1970
Colistimethate sodium	Colistin	Polymyxins	ND	Pulmonary infections	1970
Norethindrone acetate	Norethindrone	Progestogens	ND	Oncological disease	1970
Cefaloglycin; Cephaloglycin	Cephalosporin	Glycopeptides; Cephalosporins	ND	Antibacterial	1970
Flucloxacillin; Floxacillin	Penicillin	Penicillins	ND	Antibacterial	1970
Mecillinam; Amdinocillin; Pivmecillinam; Amdinocillin pivoxil	Penicillin	Penicillins	ND	Antibacterial	1970

Clindamycin	Lincomycin	Lincosamides	ND	Antibacterial	1970
Streptozocin; Streptozotocin; Stz	Streptozocin	Glucosamine	N	Oncological disease	1970
Levodopa	L-Dopa	Catecholamines	N	Dietary supplement	1970
Plicamycin	Plicamycin	Aureolic acid family	N	Anticancer	1970
Cytosine arabinoside; Ara-c	Spongothymidine	Cytosine analog (with an arabinose sugar)	S*	Oncological disease	1969
Troleandomycin; Oleandocetine	Erythromycin	Macrolides	ND	Antibacterial	1969
Cytarabine	Cytosine	Cytosine analogues	S*	cancers of white blood cells	1969
Viomycin sulfate	Viomycin sulfate	Tuberactinomycin family	N	Antibacterial	1968
Physostigmine; Eserine	Physostigmine	Pyrroloindole alkaloids	N	Neurological disease	1968
Deferoxamine mesylate	Deferoxamine	Hydroxamate siderophores	N	acute iron poisoning	1968
Doxycycline	Oxytetracycline	Tetracyclines	ND	Antibacterial	1967
Propranolol	Isoprenaline	Catecholamines	ND	Cardiovascular disease	1967
Epicillin	Penicillin	Penicillins	ND	Antibacterial	1967
Pentazocine	Morphine	Opiate alkaloids	N	Neurological disease	1967
Daunorubicin; Daunomycin; Rubomycin c	Daunorubicin	Anthracyclines	N	Oncological disease	1967
Epinephrine bitartrate	Adrenaline	Catecholamines	N	Immunological, inflammatory and related disease - Inflammatory disease	1967
Thioguanine	Purine	Purine	S*	Oncological disease	1966
Doxorubicin; Doxorubicin hydrochloride; Hydroxydaunorubicin	Doxorubicin	Anthracyclines	N	Oncological disease	1966
Bleomycin; Bleomycin a2; Bleomycin b2)	Bleomycin	Peptide-polyketides	N	Oncological disease	1966
Amphotericin	Amphotericin	Polyenes	N	Antibacterial	1966
Clomocycline	Tetracycline	Tetracyclines	ND	Antibacterial	1965
Prednisone	Cortisone	Steroid derivatives (Corticosteroids)	ND	Oncological disease	1965
Hydroxyprogesterone	Progesterone	Progestogens	ND	Oncological disease	1965
Fluoxymesterone	Cortisol	Glucocorticoids	ND	Oncological disease	1965
Ethinyl estradiol	Cholesterol	Sterols	ND	Antineoplastic	1965
Ethinyl estradiol	Estradiol	Anabolic-androgenic steroids	ND	Antineoplastic	1965
Vinblastine; Vlb; Vinca leukoblastine sulfate; Vbl	Vinblastine	Vinca alkaloids	N	Oncological disease	1965
Oxytetracycline	Oxytetracycline	Tetracyclines	N	Antibacterial	1965
Testosterone	Testosterone	Anabolic-androgenic steroids	N	Anticancer	1965
Cefroxadine	Cephalosporin	Glycopeptides; Cephalosporins	ND	Antibacterial	1964
Dromostanolone	Anabolic androgenic steroid similar to testosterone	Anabolic-androgenic steroids	ND	Oncological disease	1964
Cefalotin sodium; Cephalothin	Cephalosporin	Glycopeptides; Cephalosporins	ND	Antibacterial	1964
Cefacetrile; Cephacetrile	Cephalosporin	Glycopeptides; Cephalosporins	ND	Antibacterial	1964

Ceftezole; Ceftezol	Cephalosporin	Glycopeptides; Cephalosporins	ND	Antibacterial	1964
Cycloserine	Cycloserine	Isoxazolidinones (cyclic derivative of serine)	N	Antibacterial	1964
Nystatin	Nystatin	Polyenes	N	Antifungal	1964
Neomycin	Neomycin	Aminoglycosides	N	Antibacterial	1964
Lincomycin	Lincomycin	Lincosamides	N	Antibacterial	1964
Dactinomycin; Actinomycin d	Actinomycin d	Actinomycine peptide lactones	N	Oncological disease	1964
Polymyxin b sulfate	Polymyxin b sulfate	Polymyxins	N	Antibacterial	1964
Erythromycin	Erythromycin	Macrolides	N	Antibacterial	1964
Aminocaproic acid	Lysine	Amino acids with acyclic hydroxyl side chain & derivatives	ND	bleeding disorders	1964
Trioxsalen	Trioxsalen	Furanocoumarins	N	phototherapy treatment of vitiligo and hand eczema	1964
Lymecycline	Tetracycline	Tetracyclines	ND	Antibacterial	1963
Tetracycline	Tetracycline	Tetracyclines	N	Antibacterial	1963
Vincristine; Vcr; Leurocristine	Vincristine	Vinca alkaloids	N	Oncological disease	1963
Fusafungine	Fusafungine	Hexa-peptide with unusual amino acid	N	Antibiotic	1963
Fluocinolone acetonide	Cortisol	Glucocorticoids	ND	reduce skin inflammation and relieve itching	1963
Idoxuridine	Deoxyuridine	Uridine analogues	ND	anti-herpesvirus antiviral drug	1963
Fluorouracil	Arabinosyl nucleosides	Pyrimidine analogues	S*	Oncological disease	1962
Griseofulvin; Grisovin	Griseofulvin	Benzofuran cycloalkene	N	Antifungal	1962
Fusidic acid	Fusidic acid	Steroid derivatives (fusidane steroids)	N	Antibacterial	1962
Stanozolol	Dihydrotestosterone	Androgens	ND	anaemia and hereditary angioedema	1962
Ampicillin; Pivampicillin	Penicillin	Penicillins	ND	Antibacterial	1961
Tranylcypromine sulfate	Amphetamine	Phenethylamine class	ND	Major depressive episode without melancholia	1961
Spectinomycin	Spectinomycin	Aminocyclitols	N	Antibiotic	1961
Mithramycin	Mithramycin	Aureolic acid family	N	Anticancer	1961
Deserpidine	Deserpidine	Rauwolfia alkaloids	N	Cardiovascular disease	1961
Colchicine	Colchicine	Colchicine alkaloids	N	Oncological disease	1961
Chromomycin a3	Chromomycin a3	Anthraquinones	N	Oncological disease	1961
Meticillin; Methicillin	Penicillin	Penicillins	ND	Antibacterial	1960
Ergotamine	Ergotamine	Ergopeptides	N	Neurological disease	1960
Demeclocycline	Demeclocycline	Tetracyclines	N	Antibacterial	1960
Oxymorphone	Thebaine	Opiate alkaloids	ND	Neurological disease	1959
Oxymorphone	Codeine	Opiate alkaloids	ND	Neurological disease	1959
Virginiamycin	Pristinamycin IIA	Romidepsin analogues	ND	Antibiotic	1959
Nadrolone phenylpropionate	Nadrolone phenylpropionate	Anabolic-androgenic steroids	ND	Anticancer	1959
Nandrolone phenpropionate	Nandrolone	Anabolic-androgenic steroids	ND	Oncological disease	1959
Triamcinolone	Cortisol	Glucocorticoids	ND	Oncological disease	1958

Medroxyprogesterone acetate	17-hydroxyprogeste rone	Glucocorticoids	ND	Oncological disease	1958
Dexamethasone	Cortisol	Glucocorticoids	ND	Oncological disease	1958
Colistin	Colistin	Polymyxins	N	Antibiotic	1958
Mitomycin c; Mitomycin	Mitomycin	Mitomycins	N	Oncological disease	1956
Thiamphenicol	Chloramphenicol	Chloramphenicol alkaloids	ND	Antibiotic	1955
Methylprednisolone	Prednisolone	Glucocorticoids	ND	Oncological disease	1955
Reserpine	Reserpine	Rauwolfia alkaloids	N	Cardiovascular disease	1955
Novobiocin	Novobiocin	Aminocoumarins	N	Antibacterial	1955
Spiramycin	Spiramycin	Macrolides	N	Antibiotic	1955
Pinitol	Pinitol	Cyclitols	N	Expectorant	1955
Sarkomycin	Sarkomycin	Sarkomycins	N	Anticancer	1954
Warfarin	Dicoumarol	4-Hydroxycoumarins	N	Cardiovascular disease	1954
Methylmorphine	Methylmorphine	Opiate alkaloids	N	Neurological disease	1954
Carzinophilin	Carzinophilin	Azinomycins	N	Oncological disease	1954
Methoxsalen	Methoxsalen	Furanocoumarins	N	psoriasis, eczema, vitiligo, and some cutaneous lymphomas	1954
Mercaptopurine	Purine	Purine	S*	Oncological disease	1953
Vitamin b3	Vitamin b3	Nicotinic acids	N	Lipid disorders, cardiovascular disease	1953
Methotrexate sodium	Folic acid	Folate analogues	S*	antimetabolite, antifolate	1953
Vitamin a	Vitamin a	Ionone polyisoprene	N	Night blindness	1953
Vitamin c	Vitamin c	Sugar acids	N	Vitamin C deficiency	1953
Vitamin b1	Vitamin b1	Aminopyrimidine thiazoles	N	Vitamin B1 Deficiency	1953
Vitamin b6	Vitamin b6		N	Vitamin B6 deficiency	1953
Leucovorin calcium	Leucovorin calcium	Folinic acid analogues	N	cancer chemotherapy	1952
Quinidine	Quinidine	5-aminoquinolines	N	Antiarrhythmic	1950
Chlortetracycline; Chlorotetracycline	Chlortetracycline	Tetracyclines	N	Antibacterial	1950
Norepinephrine bitartrate	Tyrosine	Aromatic amino acids	ND	attention-deficit/hyperactivity disorder, depression, and hypotension	1950
Cortisone acetate	Cortisone acetate	Corticosteroids	N	Antiinflammatory	1950
Bacitracin	Bacitracin	Cyclic polypeptides	N	Antibacterial	1948
Caffeine; Ergotamine tartrate	Caffeine	Xanthine analogues	N	vascular headaches	1948
Caffeine; Ergotamine tartrate	Ergotamine tartrate	Ergopeptides	N	vascular headaches	1948
Phenoxymethylpenicillin; Penicillin v	Penicillin	Penicillins	ND	Antibacterial	1947
Streptomycin sulfate	Streptomycin sulfate	Aminoglycosides	N	Antibacterial	1946
Folic acid	Folic acid	Folate analogues	N	Folate deficiency	1946
Methamphetamine	Amphetamine	Phenethylamine class	ND	Attention Deficit Disorder with Hyperactivity	1943
Methamphetamine	Ephedrine	Phenethylamine class	ND	Attention Deficit Disorder with Hyperactivity	1943
Gramicidin s	Gramicidin	Linear pentadecapeptides	ND	Antibiotic	1942
Curare; Delacurarine; Jexin; Tubarine	Tubocurarine	Bisbenzylisoquinolines	ND	Skeletal muscle relaxant	1942

Benzylpenicillin; Penicillin g	Penicillin	Penicillins	N	Antibacterial	1942
Estrogens, conjugated	Estrogens, conjugated	Oestrogens	N	hot flashes, and burning, itching, and dryness of the vagina and surrounding areas	1942
Chloroquine	Quinine	5-aminoquinolines	ND	Antiparasitic	1940
Quinine	Quinine	5-aminoquinolines	N	Antiparasitic	1940
Pilocarpine	Pilocarpine	Diazole dihydrofuran	N	Glaucoma. For the treatment of radiation-induced dry mouth	1900
Aspirin; Acetylsalicylic acid; Asa	Salicylic acid	Monohydroxybenzoic acids	ND	Immunological, inflammatory and related disease - Inflammatory disease	1898
Heroin; Diacetylmorphine	Morphine	Opiate alkaloids	N	Neurological disease	1898
Noscapine (narcotine)	Noscapine (narcotine)	Phthalideisoquinoline alkaloids	N	Antitussive	1895
Cocaine; Benzoylmethyl ecgonine	Cocaine	Tropane alkaloids	N	Neurological disease	1884
Cotinine	Cotinine	Tobacco alkaloids	N	Insecticide	1560

Supplementary Table S3 List of the nature-related small molecule drugs in clinical trials and their natural product leads. Nature-related small molecule drugs include natural products and natural product semi-synthetic derivatives, mimetics, and pharmacophore-guided synthetic molecules.

Drug Name	Drug Lead	Lead Scaffold	Drug Type	Targeted Disease	Clinical Phase
1,5-DCQA (1,5-di-O-caffeoylquinic acid)	1,5-DCQA (1,5-di-O-caffeoylquinic acid)	Phenylpropanoids	N	Antiviral	Clinical Trial
10-Hydroxycamptothecin	Camptothecin	Camptothecin analogues	ND	Oncological disease	Phase II
1-deoxynojirimycin (AT2200, moranoline)	Nojirimycin	Imino sugars	ND	Cardiovascular and metabolic disease	Phase I
4-Methylumbelliferone	Umbelliferone	Coumarins	ND	Antiviral	Phase II
9-AC (IDEC-132)	Camptothecin	Camptothecin analogues	ND	Oncological disease	Phase II
A 366833	Epibatidine	Epibatidine alkaloids	ND	Analgesic	Phase I
ABJ879	Patupilone (epothilone B)	Polyketide macrolactones	ND	Oncological disease	Phase I
ABT-594	Epibatidine	Epibatidine alkaloids	ND	Neurological disease	Phase II
ABT-869	Zeatin (Cytokinins)	Purine analogues adenine-type	ND	Anticancer	Phase I
AE-941	Squalamine	Aminosterols	ND	Oncological disease	Phase III
AEE-788	Zeatin (Cytokinins)	Purine analogues adenine-type	ND	Oncological disease	Phase II
AG3340	AG3340	Hydroxamic acid derivatives	S*/NM	Cancer	Phase III
AG7088	AG7088	Peptide aldehydes	S*/NM	Common cold	Phase II
Allyl isothiocynate	Allyl isothiocynate	Isothiocyanates	N		Clinical Trial
Alvespimycin (17-DMAG, KOS-1022, NSC-707545)	Geldanamycin	Ansamycins	ND	Oncological disease	Phase II
AMG706	Zeatin (Cytokinins)	Purine analogues adenine-type	ND	Anticancer	Phase II
Aminocandin (NXL-201, IP960, HMR-3270)	Deoxymulundocandin	Echinocandin type peptides	ND	Antifungal	Phase I
Arenastatin A (cryptophycin-24)	Arenastatin A (cryptophycin-24)	Cyclic depsipeptides	N	Oncological disease	Phase I
Artemisone (BAY 44-9585)	Artemisinin	Trioxanes	ND	Antiparasitic	Phase II
Arterolane (RBx11160, trioxolane, OZ-277)	Artemisinin	Trioxanes	ND	Antiparasitic	Phase II
AVE-8062 (AC-7700, AVE8062A)	Combretastatin A-4	Dihydrostilbenoids	ND	Oncological disease	Phase III
AVE-8063	Combretastatin A-4	Dihydrostilbenoids	ND	Oncological disease	Phase I
AVE-8064	Combretastatin A-4	Dihydrostilbenoids	ND	Oncological disease	Phase I
AVE9633 (anti CD33-DM4)	Ansamitocin P-3	Ansamycins	ND	Oncological disease	Phase I
Axitinib (AG013736)	Zeatin (Cytokinins)	Purine analogues adenine-type	ND	Anticancer	Phase II
Azamulin	Pleuromutilin	Pleuromutilin scaffold	ND	Antibiotic	Phase I
AZD-0530 (AZM-475271)	Zeatin (Cytokinins)	Purine analogues adenine-type	ND	Anticancer	Phase I
BAL-19403	Erythromycin	Macrolides	ND	Antibacterial	Clinical Trial
BAY-57-9352	Zeatin (Cytokinins)	Purine analogues adenine-type	ND	Anticancer	Phase I
BC-3205	Pleuromutilin	Pleuromutilin scaffold	ND	Antibacterial	Phase I

BC-3205	Pleuromutilin	Pleuromutilin scaffold	ND	Antibacterial	Phase II
BC-3781.	Pleuromutilin	Pleuromutilin scaffold	ND	Antibiotic	Phase II
BC-7013	Pleuromutilin	Pleuromutilin scaffold	ND	Antibacterial	Phase I
Becatecarin (XL-119, NSC 655649, BMY-27557)	Rebeccamycin (staurosporine)	Staurosporine alkaloids	ND	Oncological disease	Phase II
Bengamide A	Bengamide	Bengamides	ND	Oncological disease	Phase I
Berubicin (RTA744, WP744)	Doxorubicin	Anthracyclines	ND	Oncological disease	Phase II
Beta-lapachone (ARQ-501, ARQ 501)	Beta-lapachone (lapachol)	Quinones	ND	Oncological disease	Phase II
Betulinic acid (ALS-357)	Betulinic acid (ALS-357)	Steroid derivatives (steroid acids)	N	Oncological disease	Phase I
Bevirimat (PA-457, DSB, 3',3'-Dimethylsuccinyl-betulinic acid, dimethyl succinyl betulinic acid)	Triterpenoid betulinic acid	Steroid derivatives (steroid acids)	ND	Antiviral	Phase II
Bizelesin	CC-1065	Duocarmycins	ND	Oncological disease	Phase I
BMS-188797	Paclitaxel	Taxanes	ND	Oncological disease	Phase II
BMS-247550	Epothilone B	Polyketide macrolactones	ND	Anticancer	Phase III
BMS-250749	Camptothecin	Camptothecin analogues	ND	Oncological disease	Phase I
BMS-275291	BMS-275291	Hydroxamic acids & derivatives	S*/NM	Cancer	Phase III
BMS-310705 (21-aminoepothilone B)	Patupilone (epothilone B)	Polyketide macrolactones	ND	Oncological disease	Phase I
BMS-582664	Zeatin (Cytokinins)	Purine analogues adenine-type	ND	Anticancer	Phase I
BMS-599626	Zeatin (Cytokinins)	Purine analogues adenine-type	ND	Anticancer	Phase I
BNP-1350	Camptothecin	Camptothecin analogues	ND	Oncological disease	Phase III
Bosutinib (SKI-606)	Zeatin (Cytokinins)	Purine analogues adenine-type	ND	Anticancer	Phase I
Bruceantin (NSC-165, 563)	Bruceantin (NSC-165, 563)	Quassinoid diterpenes	N	Oncological disease	Phase II
Bryostatin 1	Bryostatin 1	Tetracyclic heptadecane skeletons	ND	Oncological disease	Phase II
Bryostatin 1/AraC	Bryostatin 1/AraC	Tetracyclic heptadecane skeletons	N	Oncological disease	Phase I
Bryostatin 1/Fara	Bryostatin 1/Fara	Tetracyclic heptadecane skeletons	N	Oncological disease	Phase I
Bryostatin 1/paclitaxel	Bryostatin 1/paclitaxel	Tetracyclic heptadecane skeletons	N	Oncological disease	Phase II
Bryostatin 1/vincristine	Bryostatin 1/vincristine	Tetracyclic heptadecane skeletons	N	Oncological disease	Phase I
Cabazitaxel (XRP-6258, TXD-258, RPR-116258A)	Paclitaxel	Taxanes	ND	Oncological disease	Phase III
Canertinib (CI-1033, PD183805)	Zeatin (Cytokinins)	Purine analogues adenine-type	ND	Anticancer	Phase II
Cannabidiol	Cannabidiol	Cannabinoids	N	Anxiety and psychosis	Phase II
Capsaicin (ALGRX 4975)	Capsaicin (ALGRX 4975)	Capsaicinoids	N	Neurological disease	Phase III
Carfilzomib (CFZ)	Epoxomycin (peptide epoxiketone)	Epoxyketone containing oligopeptides	S/NM	Multiple myeloma	Phase III
Castor oil	Castor oil	Fatty acids	N	Constipation	Clinical Trial
Cediranib (AZD-2171)	Zeatin (Cytokinins)	Purine analogues adenine-type	ND	Anticancer	Phase II
Ceftaroline acetate (PPI-0903, TAK-599)	Cephalosporin nucleus,	Glycopeptides; Cephalosporins	ND	Antibacterial	Phase II

	7-aminocephalosporanic acid (7-ACA), derived from cephalosporin C				
Ceftizoxime alapivoxil (AS-924)	Cephalosporin nucleus, 7-aminocephalosporanic acid (7-ACA), derived from cephalosporin C	Glycopeptides; Cephalosporins	ND	Antibacterial	Clinical Trial
Ceftobiprole (ceftobripole medocaril, BAL-5788, Ro-65-5788)	Cephalosporin nucleus, 7-aminocephalosporanic acid (7-ACA), derived from cephalosporin C	Glycopeptides; Cephalosporins	ND	Antibacterial	Phase III
Cematodin	Dolastatin 15	Depsipeptides	ND	Oncological disease	Phase II
CEP-1347 (KT-8138, KT7515)	K-252a (Staurosporine)	Staurosporine alkaloids	ND	Neurological disease	Phase III
CEP-7055	Staurosporine	Staurosporine alkaloids	ND	Oncological disease	Phase I
Cethromycin (ABT-773)	Erythromycin	Macrolides	ND	Antibacterial	Phase III
CGS-27023A	CGS-27023A	Hydroxamic acid derivatives	S*/NM	Cancer	Phase II
Civamide (cis-capsaicin, zucapsaicin, WL-1001)	Civamide (cis-capsaicin, zucapsaicin, WL-1001)	Capsaicinoids	ND	Neurological disease	Phase III
CMB 401 (hCTMO1-calicheamicin, CDP671)	Calicheamicin	Calicheamicins	ND	Oncological disease	Phase II
COL-3	Tetracycline	Tetracyclines	S*/NM	Cancer	Phase II
Combretastatin A-4 phosphate (CA4P, CA4PO4, combretastin A4 phosphate, combrestatin A4)	Combretastatin A-4	Dihydrostilbenoids	ND	Oncological disease	Phase III
Conantokin-G (CGX-1007, conantokin G)	Conantokin-G (CGX-1007, conantokin G)	Conantokins	N	Neurological disease	Phase II
Contulakin-G (CGX-1160, contulakin G)	Contulakin-G (CGX-1160, contulakin G)	Large O-glycosylated linear peptides	N	Neurological disease	Phase II
CP 7075 (IP 751, ajulemic acid, CT-3)	CP 7075 (IP 751, ajulemic acid, CT-3)	Cannabinoids	ND	Neurological disease	Phase II
CP-724714	Zeatin (Cytokinins)	Purine analogues adenine-type	ND	Anticancer	Phase I
CP-751 (CP-751,871)	Staurosporine	Staurosporine alkaloids	ND	Oncological disease	Phase III
Cryptophycin-52 (LY355703)	Taxol	Taxanes	ND	Anticancer	Phase II
Curcumin	Curcumin	Diarylheptanoids	ND	Oncological disease	Phase II
DA-5018	DA-5018	Vanilloids	ND	Neurological disease	Phase II
DA-6034	Eupatilin	O-methylated flavones	ND	Immunological, inflammatory and related disease	Phase II
Daidzein (4',7-dihydroxyisoflavone)	Daidzein (4',7-dihydroxyisoflavone)	Isoflavones	N	Oncological disease	Phase I
Dapagliflozin (BMS-512148)	Phlorizin	Dihydrochalcones	ND	Neurological disease	Phase III
Datelliptium acetate (NSC 311152)	Ellipticine	Ellipticine alkaloids	ND	Oncological disease	Phase II

Debio 9902 (ZT-1)	Huperzine	Sesquiterpene alkaloids	ND	Neurological disease	Phase II
Debio-025 (UNIL025, MeAla3EtVal4-cyclosporin)	Ciclosporin	Nonribosomal peptide cyclic structure	ND	Antiviral	Phase II
Deforolimus (MK-8669, AP-23573)	Sirolimus (rapamycin)	Macrolides	ND	Oncological disease	Phase III
Dehydelone (KOS-1584, 9,10-didehydroepothilone D)	Epothilone D	Polyketide macrolactones	ND	Oncological disease	Phase I
Dehydrodidemnin B (plitidepsin, aplidin)	Dehydrodidemnin B (plitidepsin, aplidin)	Cyclic depsipeptides	N	Oncological disease	Phase II
Depsipeptide (NSC 630176)	Romidepsin	Depsipeptide cyclic structure	N	Oncological disease	Phase II
DHA-paclitaxel	Paclitaxel	Taxanes	ND	Oncological disease	Phase III
Diflomotecan (BN-80915)	Camptothecin	Camptothecin analogues	ND	Oncological disease	Phase II
DMBX (GTS-21, DMBX-anabaseine, DMBX-A)	Anabaseine (worm toxin)	Nicotine alkaloids	ND	Neurological disease	Phase II
DRF-1042	Camptothecin	Camptothecin analogues	ND	Oncological disease	Phase II
E7974	Hemiasterlin	Tryptophan derivatives	ND	Oncological disease	Phase I
E7974	Hemiasterlin peptide	Tryptophan derivatives	ND	Oncological disease	Phase I
EC145	Vinblastine	Vinca alkaloids	ND	Oncological disease	Phase I
ECO-4601 (diazepinomicin)	ECO-4601 (diazepinomicin)	Dibenzodiazepine alkaloids	ND	Oncological disease	Phase II
Edotecarin (J-107088)	Rebeccamycin (staurosporine)	Staurosporine alkaloids	ND	Oncological disease	Phase III
Elomotecan (BN-80927, LBQ707, R-1559)	Camptothecin	Camptothecin analogues	ND	Oncological disease	Phase I
Elsamitrucin (elsamicin A)	Elsamicin A (chartreusin)	Coumarin-related chartarin disaccharide complex	ND	Oncological disease	Phase II
EndoTAG-1	Paclitaxel	Taxanes	ND	Oncological disease	Phase II
Enzastaurin (LY317615)	Staurosporine	Staurosporine alkaloids	ND	Oncological disease	Phase III
EP-420 (EP-013420)	Erythromycin	Macrolides	ND	Antibacterial	Phase II
Epigallocatechin gallate	Epigallocatechin gallate	Catechins	N	Oncological disease	Phase II
Epothilone D (KOS-862, Kosan-862, deoxyepothilone B)	Patupilone (epothilone B)	Polyketide macrolactones	ND	Oncological disease	Phase II
Everolimus (RAD-001)	Sirolimus (rapamycin)	Macrolides	ND	Oncological disease	Phase III
Exatecan (DX-8951f, exetecan, exatecan mesilate, DE-310)	Camptothecin	Camptothecin analogues	ND	Oncological disease	Phase III
Faropenem daloxate (SUN-208, BAY-56-6824)	Thienamycin	Carbapenam	ND	Antibacterial	Phase III
Flavopiridol (alvocidib, HMR 1275)	Rohitukine	Chromane alkaloids	ND	Oncological disease	Phase III
Fostriecin	Fostriecin	Phosphate esters with a long tail	N	Oncological disease	Phase I
Friulimicin B	Friulimicin B	Lipo(depsi)peptides	N	Antibacterial	Phase I
Genistein	Genistein (isoflavone)	Isoflavones	ND	Oncological disease	Phase II
Gimatecan (ST-1481)	Camptothecin	Camptothecin analogues	ND	Oncological disease	Phase II
GL-331	Podophyllotoxin	Podophyllotoxin lignans	ND	Oncological disease	Phase II
GSK-189075/KGT-1681	Phlorizin	Dihydrochalcones	ND	Neurological disease	Phase II
GW640385	GW640385	Tyrosyl-based arylsulfonamides	S/NM	Antiviral	Phase II
GW823093C (823093,	GW823093C	Fluorinated phenyl alanyl	S*/NM	Diabetes mellitus	Phase II

GW823093)	(823093, GW823093)	derivatives			
HKI-272	Zeatin (Cytokinins)	Purine analogues adenine-type	ND	Anticancer	Phase II
Homocamptothecin	Camptothecin	Camptothecin analogues	ND	Oncological disease	Phase I
Homoharringtonine (omacetaxine mepesuccinate, harringtonine, homoharringtone)	Homoharringtonine (homoharrington)	Cephalotaxine alkaloids	ND	Oncological disease	Phase III
HTI-286 (SPA-110)	Hemiasterlin	Tryptophan derivatives	ND	Oncological disease	Phase II
IdB 1016 (silipide, silybin and phosphatidylcholine complex)	Silybin	Flavanones	ND	Oncological disease	Phase II
IDN-6556	IDN-6556	Oxamyl dipeptides & analogues	S*/NM	Hepatitis C	Phase II
Ilepatril (AVE7688)	Teprotide	Nonapeptides	ND	Cardiovascular and metabolic disease	Phase III
IM862	IM862	Tryptophan derivatives	N	Anticancer	Phase III
IMGN-242; HuC242-DM4; cantuzumab mertansine	Ansamitocin P-3	Ansamycins	ND	Oncological disease	Phase II
IMGN-901 (HuN901-DM1) (a conjugate of maytansinoid DM1 and huN901)	Ansamitocin P-3	Ansamycins	ND	Oncological disease	Phase II
Indirubin	Indirubin	Bisindole alkaloids	N	Oncological disease	Phase II
Indole-3-carbinol (I3C, I-3-C)	Indole-3-carbinol (IC3, I-3-C)	Indole derivatives	N	Oncological disease	Phase I
Ingenol 3-angelate (PEP005, Ingenol 3-O-angelate)	Ingenol	Diterpenes	ND	Oncological disease	Phase II
Inotuzumab ozogamicin; CMC-544	Calicheamicin	Calicheamicins	ND	Oncological disease	Phase III
IPL512602	Contignasterol	Oxygenated steroids with unnatural 14.beta configuration	ND	Asthma	Phase II
IPL-576,092 (HMR-4011A, IPL576,092)	Contignasterol	Oxygenated steroids with unnatural 14.beta configuration	ND	Inflammatory disease	Phase II
Irofulven (MGI-114, HMAF, 6-hydroxymethylacylfulvene)	Illudin S	Illudins sesquiterpenes	ND	Oncological disease	Phase III
ISA247	Cyclosporine	Nonribosomal peptide cyclic structure	ND	Immunological, inflammatory and related disease	Phase II
Isofagomine (AT2101)	1-deoxynojirimycin	Imino sugars	ND	Cardiovascular and metabolic disease	Phase II
JC-9 (ASC-9)	Curcumin	Diarylheptanoids	ND	Anticancer	Phase II
Kahalalide F	Kahalalide F	Depsipeptides	ND	Oncological disease	Phase II
KRN-5500	Spicamycin	Purine nucleoside analogues	ND	Neurological disease	Phase II
KRN633	Zeatin (Cytokinins)	Purine analogues adenine-type	ND	Anticancer	Phase I
KRX-0601 (UCN-01, KW-2401, 7-hydroxystaurosporine)	Staurosporine	Staurosporine alkaloids	ND	Oncological disease	Phase II
KW-2189	Duocarmycin B2	Duocarmycins	ND	Oncological disease	Phase II
LAF-389 (NVP-LAF-389)	Bengamide B	Bengamides	ND	Oncological disease	Phase I
L-annamycin	Doxorubicin	Anthracyclines	ND	Oncological disease	Phase II
Larotaxel dehydrate (XRP-9881, RPR-109881A)	Paclitaxel (10-deacetyl baccatin III)	Taxanes	ND	Oncological disease	Phase III
LBM415 (NVP-PDF-713)	Actinonin	Hydroxamic acids & derivatives	ND	Antibacterial	Phase III
Lestaurtinib (CEP-701, KT-5555)	K252a (staurosporine)	Staurosporine alkaloids	ND	Oncological disease	Phase III

Lobeline (alpha-lobeline)	Lobeline (alpha-lobeline)	Piperidine alkaloids	N	Neurological disease	Phase II
Lurtotecan (NX211, GI-147211)	Camptothecin	Camptothecin analogues	ND	Oncological disease	Phase II
MAG-CPT (PNU166148)	Camptothecin	Camptothecin analogues	ND	Oncological disease	Phase I
Manoalide	Manoalide	Nonsteroidal sesterterpenoid	N	Inflammatory disease	Phase II
Maytansine	Maytansine	Ansamycins	N	Oncological disease	Phase II
MBI-3253 (celgosivir, 6-O-butanoylcastanospermine, MX-3253)	Castanospermine	Indolizine alkaloids	ND	Antiviral	Phase II
MC-1	Vitamin B6	-	ND	Cardiovascular and metabolic disease	Phase III
ME-1036 (CP5609)	Carbapenem	β-lactams	ND	Antibacterial	Phase I
Metastat (COL-3)	Tetracycline	Tetracyclines	S/NM	Anticancer	Phase II
Methylnaltrexone (MOA-728)	Naltrexone	Opiate alkaloids	ND	Neurological disease	Phase III
Midostaurin (PKC-412, CGP 41251, 4'-N-Benzoyl-staurosporine, CGP41251, PKC412)	Staurosporine	Staurosporine alkaloids	ND	Oncological disease	Phase II
Migalastat (AT1001, 1-deoxygalactonojirimycin, 1-deoxygalactostatin)	Galactonojirimycin (galactostatin)	Imino sugars	ND	Cardiovascular and metabolic disease	Phase II
Milataxel (MAC-321, TL-00139)	Paclitaxel	Taxanes	ND	Oncological disease	Phase II
Mitemcinal (mitemcinal fumarate, GM-611)	Erythromycin	Macrolides	ND	Cardiovascular and metabolic disease	Phase II
MK-0431	MK-0431	-	S*/NM	Diabetes mellitus	Phase II
MK-944a (L-756,423)	Pepstatin	Hexa-peptide with unusual amino acid	ND	Anti-HIV	Phase I
MLN2704 (a conjugate of DM1 and the antibody T-MAV)	Ansamitocin P-3	Ansamycins	ND	Oncological disease	Phase II
Moli1901 (duramycin, 2262U90)	Moli1901 (duramycin, 2262U90)	Large polycyclic peptides	N	Antibacterial	Phase II
Morphine-6-glucuronide (M6G)	Morphine	Opiate alkaloids	ND	Neurological disease	Phase III
NB-506	BE-13793C	Indolocarbazoles	ND	Oncological disease	Phase II
Nemorubicin (MMDX, PNU-152243A)	Doxorubicin	Anthracyclines	ND	Oncological disease	Phase II
NIM 811 (SDZ NIM 811, cyclosporin 29, Melle-cyclosporin)	NIM 811 (SDZ NIM 811, cyclosporin 29, Melle-cyclosporin)	Four-substituted cyclosporine analogues	ND	Antiviral	Phase I
NK-105	Paclitaxel	Taxanes	ND	Oncological disease	Phase II
NK-611	Podophyllotoxin	Podophyllotoxin lignans	ND	Oncological disease	Phase II
Noscapine (CB3304)	Noscapine	Phthalideisoquinoline alkaloids	ND	Oncological disease	Phase I
NPI-0052 (salinosporamide A)	Salinosporamide A	Salinosporamides	ND	Oncological disease	Phase I
NPI-2358	halimide	Dioxopiperazines	ND	Oncological disease	Phase I
NV-52	Isoflavone	Isoflavones	N	Immunological, inflammatory and related disease	Phase II
NVP-AUY922 (VER-52296)	Geldanamycin	Ansamycins	ND	Anticancer	Phase I
NVP-DPP728	NVP-DPP728	Cyanopyrrolidides	S*/NM	Diabetes mellitus	Phase II
NVP-LAF237	NVP-LAF237	Thienopyrimidines containing a substituted alkyl group	S*/NM	Diabetes mellitus	Phase II

NVP-LAQ824	Trapoxin	Cyclotetrapeptide	ND	Oncological disease	Phase I
NVP-LAQ824	Trichostatin	Aniline dicarboxylic acids	ND	Oncological disease	Phase I
NVP-LAQ824	Psammaplin	Disulfide bromotyrosine derivatives	ND	Oncological disease	Phase I
Obatoclax (GX15-070)	Streptorubin B (prodigiosin)	Prodiginines	ND	Oncological disease	Phase II
Omacetaxine mepesuccinate	Homoharringtonine	Cephalotaxine alkaloids	ND	Anticancer	Phase III
Omapatrilat	Teprotide	Nonapeptides	S*/NM	Hypertension	Phase II
Omega-conotoxin CVID (AM336)	Omega-conotoxin CVID (AM336)	Cyclic cysteine knot peptides	N	Neurological disease	Phase II
Ortataxel (IDN-5109, BAY-59-8862)	Paclitaxel	Taxanes	ND	Oncological disease	Phase II
OSI-930	Zeatin (Cytokinins)	Purine analogues adenine-type	ND	Anticancer	Phase I
OXi4503 (combretastatin A-1 diphosphate)	Combretastatin A-1	Dihydrostilbenoids	ND	Oncological disease	Phase I
Oxocalanolide	Calanolide	Dipyranocoumarins	ND	Antiviral	Phase I
PA-1050040 (PA-040)	Betulinic acid	Steroid derivatives (steroid acids)	ND	Anti-HIV	Phase I
Paclitaxel poliglumex (a conjugate of paclitaxel with a biodegradable polyglutamic acid)	Paclitaxel	Taxanes	ND	Oncological disease	Phase III
Panobinostat (LBH-589)	Psammaplin A	Disulfide bromotyrosine derivatives	ND	Oncological disease	Phase III
Patupilone (epothilone B, EPO-906)	Epothilone B	Polyketide macrolactones	ND	Oncological disease	Phase III
PD-332991	Zeatin (Cytokinins)	Purine analogues adenine-type	ND	Anticancer	Phase I
PDX101	Trichostatin	Aniline dicarboxylic acids	ND	Oncological disease	Phase I
PEG-CPT (PEG–camptothecin, Prothecan)	Camptothecin	Camptothecin analogues	ND	Oncological disease	Phase II
Perillyl alcohol (POH)	Perillyl alcohol (POH)	Monoterpenes	N	Oncological disease	Phase II
PF-3709270	Carbapenem	β-lactams	N	Antibacterial	Phase I
PF4548043 (KOS-2187)	Erythromycin	Macrolides	ND	Cardiovascular and metabolic disease	Phase I
Phenethyl isothiocyanate (PEITC)	Phenethyl isothiocyanate (PEITC)	Isothiocyanates	N	Oncological disease	Phase I
Phenoxodiol	Daidzein (isoflavone)	Isoflavones	ND	Oncological disease	Phase III
Plitidepsin	Plitidepsin (aplidin)	Cyclic depsipeptides	ND	Oncological disease	Phase II
PM00104/50	Jorumycin	Macrolides	ND	Oncological disease	Phase I
PM02734	Kahalalide F	Depsipeptides	ND	Oncological disease	Phase I
Podophyllotoxin	Podophyllotoxin	Podophyllotoxin lignans	N	Anticancer	Phase I
PPL-100	PPL-100	-	S/NM	Antiviral	Phase I
Prostratin	Prostratin	Tigliane diterpenes	N	Antiviral	Phase I
Protoveratine A	Protoveratine A	Veratrum alkaloids	N	Hypertension	Clinical Trial
Protoveratine B	Protoveratine B	Veratrum alkaloids	N	Hypertension	Clinical Trial
PS-519	Lactacystin	γ-lactams	ND	acute stroke	Phase I
PTK-0796 (MK-2764)	Tetracycline	Tetracyclines	ND	Antibacterial	Phase II
Pyridoxamine (K-163)	Vitamin B6	-	ND	Cardiovascular and	Phase II

				metabolic disease	
PZ-601 (SMP-601, SM-216601)	Carbapenem	β-lactams	N	Antibacterial	Phase II
QS-21A	QS-21A	Saponins	N	Immunological disease	Phase II
QS-21B	QS-21B	Saponins	N	Immunological disease	Phase III
Ramoplanin (ramoplanin factor A2)	Ramoplanin	Glycolipodepsipeptides	ND	Antibacterial	Phase III
Retaspimycin (IPI-504, 17-AAG hydroquinone salt)	Geldanamycin	Ansamycins	ND	Oncological disease	Phase II
Romidepsin (depsipeptide, FR-901228, FK-228)	Romidepsin	Depsipeptide cyclic structure	ND	Oncological disease	Phase III
Rostafuroxin (PST 2238)	Ouabain	Cardiac glycosides	ND	Cardiovascular and metabolic disease	Phase II
Rubitecan (9-nitrocamptothecin, 9-NC, RFS2000, 9-Nitro-CPT)	Camptothecin	Camptothecin analogues	ND	Anticancer	Phase III
Ruboxistaurin (LY333531)	Staurosporine	Staurosporine alkaloids	ND	Cardiovascular and metabolic disease	Phase III
RWJ-442831	Cephalosporin nucleus, 7-aminocephalosporanic acid (7-ACA), derived from cephalosporin C	Glycopeptides; Cephalosporins	ND	Antibacterial	Phase I
S23906-1	Acronycine	Acridone alkaloids	ND	Oncological disease	Phase I
Sabarubicin (MEN-10755)	Doxorubicin	Anthracyclines	ND	Oncological disease	Phase II
Sagopilone (ZK-EPO, ZK-219477)	Epothilone B	Polyketide macrolactones	ND	Oncological disease	Phase II
SCH 530348	Himbacine	Tetracyclic piperidine alkaloids	ND	Cardiovascular and metabolic disease	Phase III
Scillaren A	Scillaren A	Scilla glycosides	N	Cancer	Clinical Trial
Seliciclib (Rosco, R-roscovitine, CYC202)	Zeatin (Cytokinins)	Purine analogues adenine-type	ND	Oncological disease	Phase II
Silatecan (DB-67, 7-silylcamptothecan, AR-67)	Camptothecin	Camptothecin analogues	ND	Oncological disease	Phase I
SN2310	Camptothecin	Camptothecin analogues	ND	Oncological disease	Phase I
Soblidotin (YHI-501, TZT-1027, auristatin PE)	Dolastatin 10	Peptide	ND	Oncological disease	Phase II
SPP100	SPP100	Azaindole scaffolds	S*/NM	Hypertension	Phase III
Squalamine	Squalamine	Aminosterols	ND	Oncological disease	Phase III
SU-14813	Zeatin (Cytokinins)	Purine analogues adenine-type	ND	Anticancer	Phase II
SU-6668	Zeatin (Cytokinins)	Purine analogues adenine-type	ND	Anticancer	Phase II
Sulopenem (CP-70429)	Carbapenem	β-lactams	N	Antibacterial	Phase I
TAFA-93	Sirolimus (rapamycin)	Macrolides	ND	Immunological, inflammatory and related disease	Phase I
Tafluposide	Epipodophyllotoxin	Podophyllotoxin lignans	ND	Oncological disease	Phase I
Tanespimycin (17-AAG, KOS-953, NSC-330507, 17-allylaminogeldanamycin)	Geldanamycin	Ansamycins	ND	Oncological disease	Phase III
Taribavirin (ribamidine)	Showdomycin	Imidazole ribosides	ND	Antiviral	Phase II
Taribavirin (ribamidine)	Pyrazomycin	C-nucleosides	ND	Antiviral	Phase II
Tasidotin (synthadotin, ILX-651)	Dolastatin 15	Depsipeptides	ND	Oncological disease	Phase II
TD-1792	Cephalosporin in	Glycopeptides; Cephalosporins	ND	Antibacterial	Phase II

	vancomycin-cephalosporin				
Tebipenem pivoxil (ME-1211, L-084)	Carbapenem	β-lactams	ND	Antibacterial	Phase III
Tesetaxel (DJ-927)	Paclitaxel	Taxanes	ND	Oncological disease	Phase II
Tetrodotoxin (anhydrotetrodotoxin 4-epitetrodotoxin, tetrodonic acid, TTX)	Tetrodotoxin (anhydrotetrodotoxin 4-epitetrodotoxin, tetrodonic acid, TTX)	Guanidinium-pyrimidine-cyclohexane scaffold	N	Neurological disease	Phase III
TMC-114	TMC-114	Tetrahydrofuranyl-urethane analogues	S/NM	Antiviral	Phase III
TNP-470 (takeda neoplastic product 470)	Fumagillin	Bisabolane sesquiterpenes	ND	Oncological disease	Phase III
Tomopenem (CS-023, RO4908463, R1558)	Carbapenem	β-lactams	ND	Antibacterial	Phase II
TPI-287 (NBT-287)	Paclitaxel	Taxanes	ND	Oncological disease	Phase II
Trastuzumab-DM1 (T-MCC-DM1) (a conjugate of a humanized monoclonal antibody trastuzumab with DM1)	Ansamitocin P-3	Ansamycins	ND	Oncological disease	Phase II
Triphendiol (NV-196), analogue of phenoxodiol	Isoflavone	Isoflavones	ND	Oncological disease	Phase I
Trodusquemine (MSI-1436)	Trodusquemine (MSI-1436)	Aminosterols	N	Cardiovascular and metabolic disease	Phase I
TS-033	Phlorizin	Dihydrochalcones	ND	Neurological disease	Phase II
Vandetanib (zactima, ZD-6474)	Zeatin (Cytokinins)	Purine analogues adenine-type	ND	Anticancer	Phase III
Vatalanib (PTK787, ZK222584)	Zeatin (Cytokinins)	Purine analogues adenine-type	ND	Anticancer	Phase III
Vinflunine (vinflunine ditartrate)	Vinblastine	Vinca alkaloids	ND	Oncological disease	Phase III
Voclosporin (ISA-247, R1524)	Cyclosporin A (cyclosporine A)	Nonribosomal peptide cyclic structure	ND	Immunological, inflammatory and related disease	Phase II
Vorinostat	Trichostatin	Aniline dicarboxylic acids	ND	Anticancer	Phase II
VX-950	VX-950	Tri-peptides	S*/NM	Hepatitis C	Phase II
XR-9051	XR-334	Diketopiperazines	ND	Oncological disease	Phase I
YM-543	Phlorizin	Dihydrochalcones	ND	Neurological disease	Phase II
Ziconotide	Ziconotide	Conotoxins	N	Chronic pain	Phase III

Supplementary Table S4 Statistics of the 62 drug lead productive scaffolds in the scaffold hunter derived molecular scaffold trees of 134,097 natural products and 411 natural product leads. These drug lead productive scaffolds are labeled as DS1 to DS62.

Drug Lead Productive Scaffold or Scaffold Parent-Child Sub-branch (Branch)	Drug-Productive Scaffold(s) in DS	No of Scaffolds	No of Drug Leads (Approved/Clinical Trial)	No of Derived Drugs (Approved/Clinical Trial)
DS1 (1)	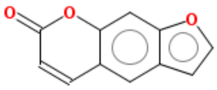	1	2 (2/0)	3 (3/0)
DS2 (2)	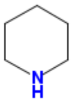	1	2 (1/2)	5 (2/3)
DS3 (2)	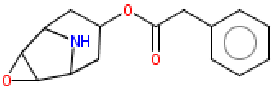 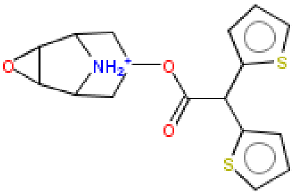	2	3 (3/0)	5 (5/0)
DS4 (2)	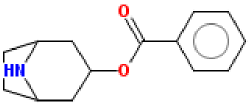 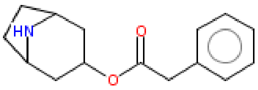	2	3 (3/0)	6 (6/0)
DS5 (2)	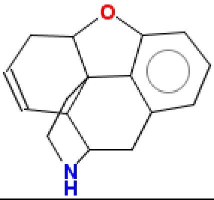 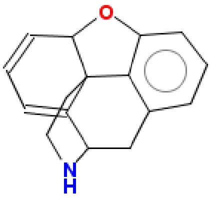 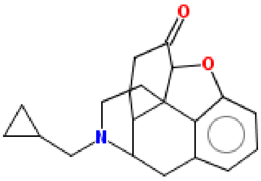	3	4 (4/2)	18 (16/2)

DS6 (3)	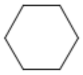	1	3 (3/0)	3 (3/0)
DS7 (3)	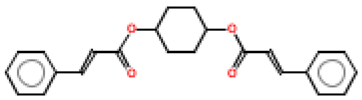 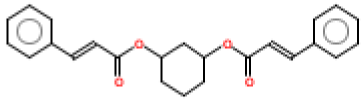	2	2 (1/1)	2 (1/1)
DS8 (3)	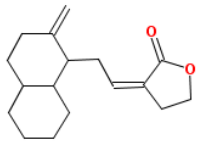 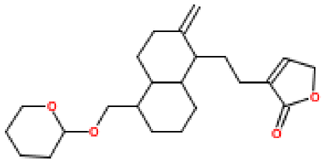	2	2 (2/0)	2 (2/0)
DS9 (3)	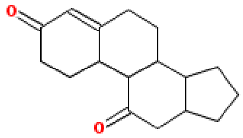	1	2 (2/0)	3 (3/0)
DS10 (3)	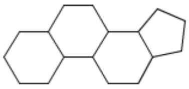 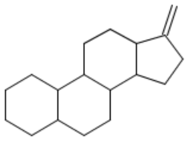 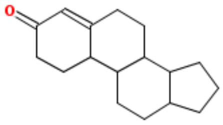 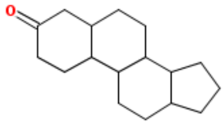 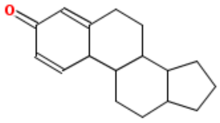 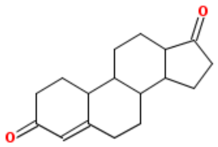 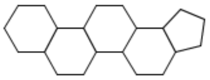 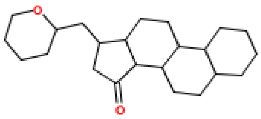 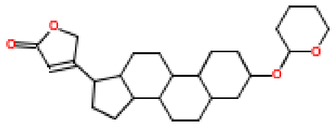	17	39 (32/8)	84 (72/12)

	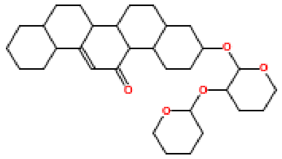 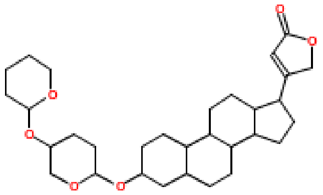 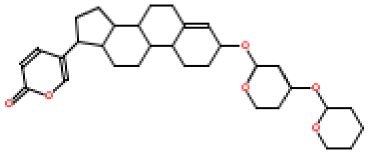 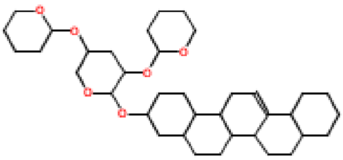 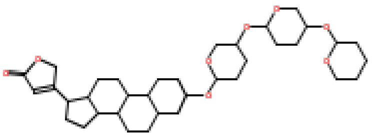 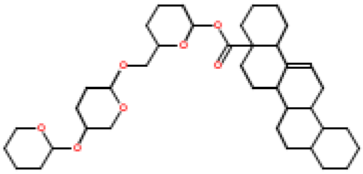 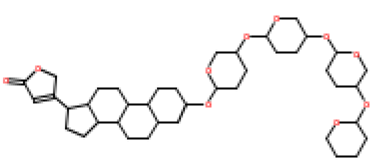 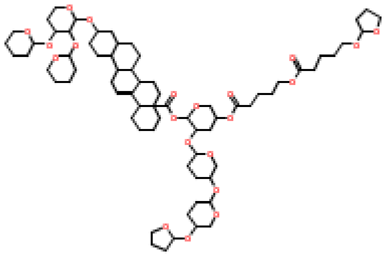			
DS11 (4)	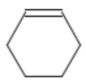	1	2 (1/1)	4 (3/1)
DS12 (4)	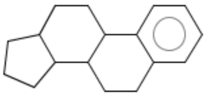 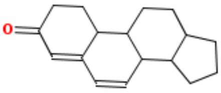 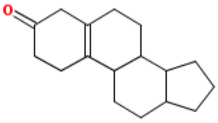	3	3 (3/0)	21 (21/0)
DS13 (4)	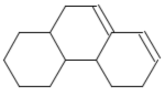 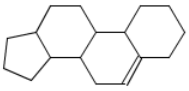 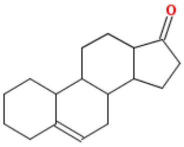	3	5 (5/0)	11 (11/0)

DS14 (4)	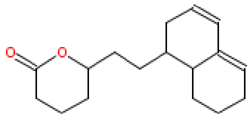	1	2 (2/0)	7 (7/0)
DS15 (5)	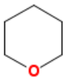	1	3 (3/0)	5 (5/0)
DS16 (5)	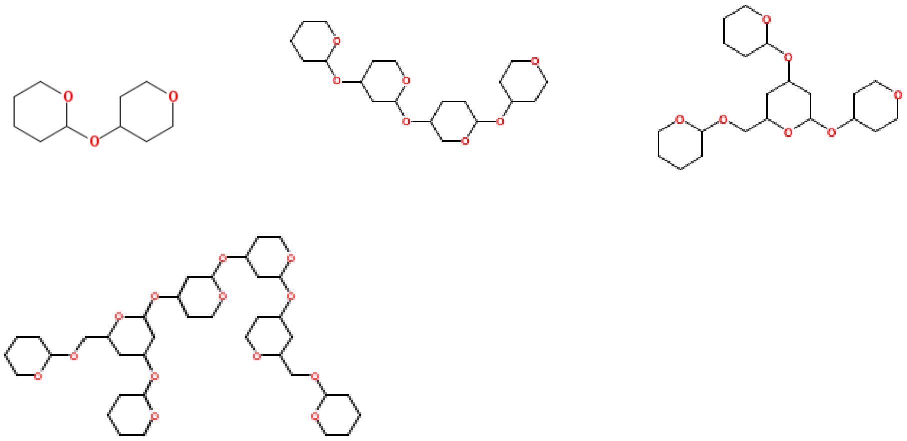	4	4 (4/0)	5 (5/0)
DS17 (5)	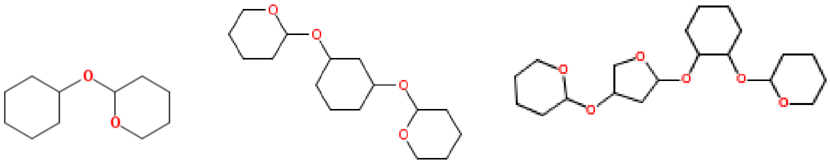	3	7 (7/0)	11 (11/0)
DS18 (5)	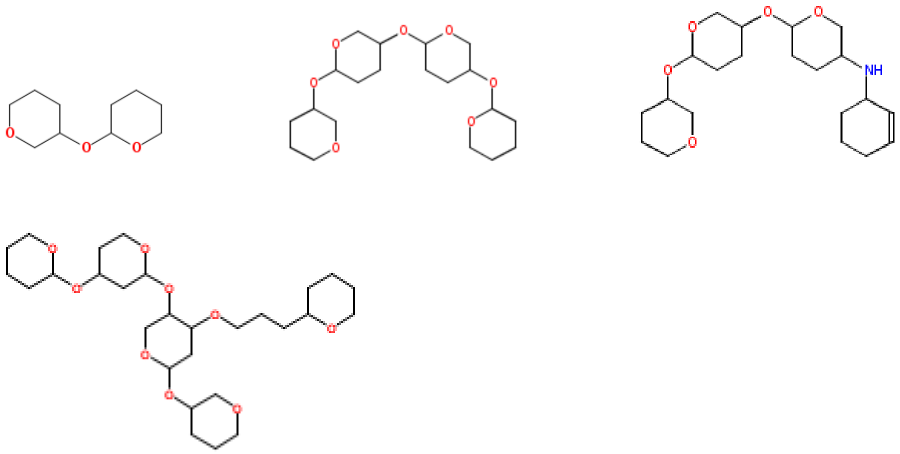	4	4 (4/0)	14 (14/0)

DS19 (5)	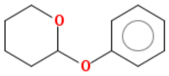 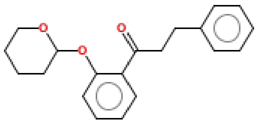	2	2 (1/1)	5 (1/4)
DS20 (6)	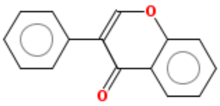	1	2 (1/2)	6 (1/5)
DS21 (7)	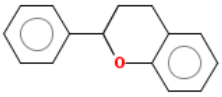 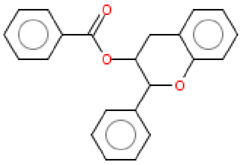	2	2 (2/1)	3 (2/1)
DS22 (8)	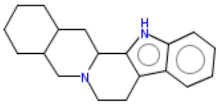 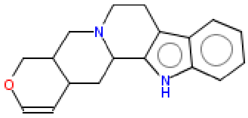 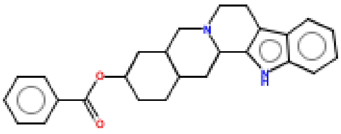 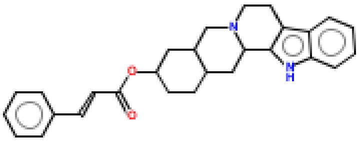	4	5 (5/0)	5 (5/0)
DS23 (9)	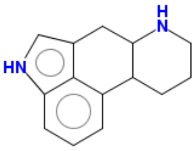 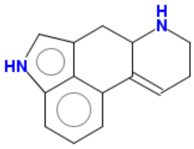 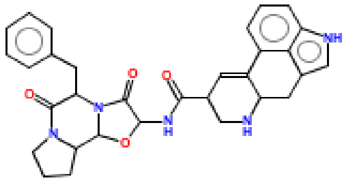	3	3 (3/0)	8 (8/0)
DS24 (9)	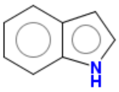	1	7 (4/3)	15 (10/5)

DS25 (9)	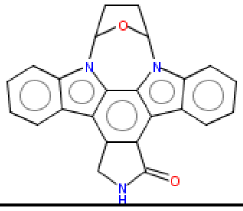 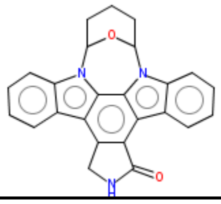	2	2 (1/2)	11 (3/8)
DS26 (10)	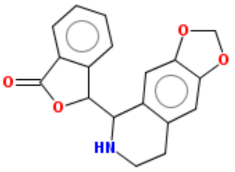	1	2 (2/1)	3 (2/1)
DS27 (11)	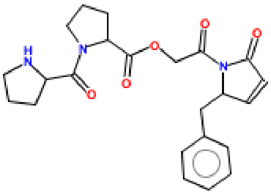 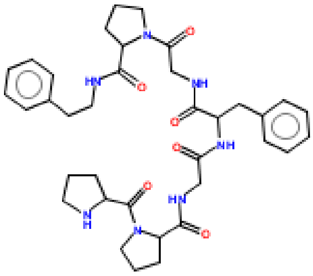	2	2 (1/1)	3 (1/2)
DS28 (12)	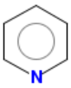	1	4 (4/1)	7 (5/2)
DS29 (12)	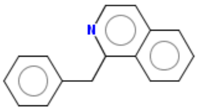 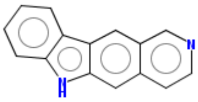	2	2 (2/1)	3 (2/1)
DS30 (13)	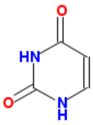	1	2 (2/0)	3 (3/0)
DS31 (13)	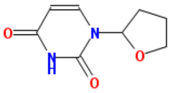 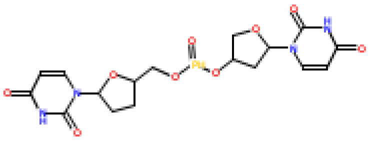	2	5 (5/0)	9 (9/0)
DS32 (13)	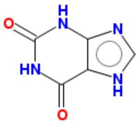	1	4 (4/0)	7 (7/0)

DS33 (14)	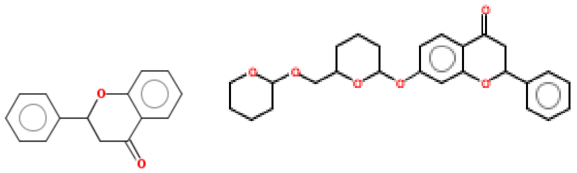	2	2 (2/0)	3 (3/0)
DS34 (15)	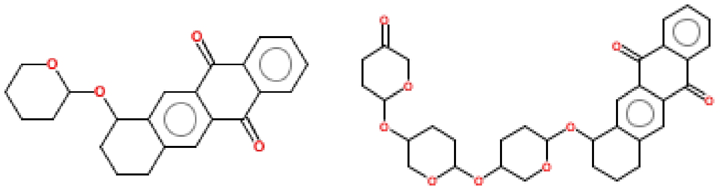	2	3 (3/1)	13 (9/4)
DS35 (16)	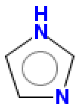	1	3 (3/0)	9 (9/0)
DS36 (16)	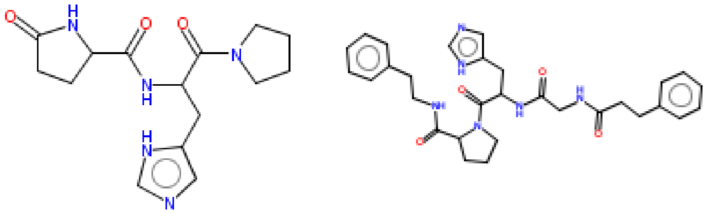	2	2 (2/0)	2 (2/0)
DS37 (17)	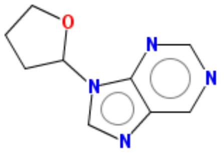	1	3 (3/0)	5 (5/0)
DS38 (17)	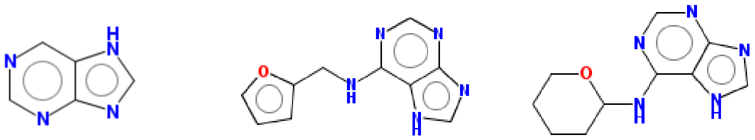	3	4 (3/2)	33 (11/22)
DS39 (18)	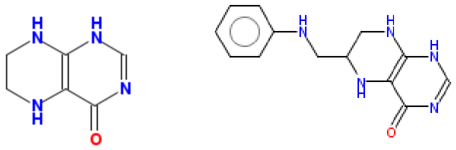	2	2 (2/0)	2 (2/0)

DS40 (19)	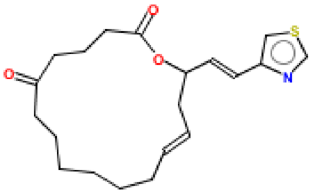 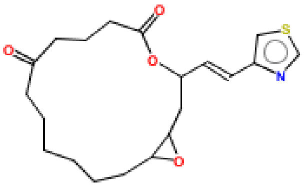	2	2 (1/2)	8 (1/7)
DS41 (20)	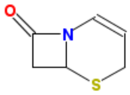	1	2 (2/1)	56 (51/5)
DS42 (20)	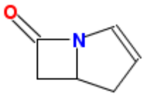	1	2 (1/2)	15 (8/7)
DS43 (20)	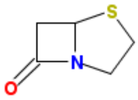	1	3 (3/0)	31 (31/0)
DS44 (21)	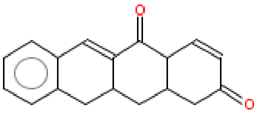	1	4 (4/1)	13 (10/3)
DS45 (21)	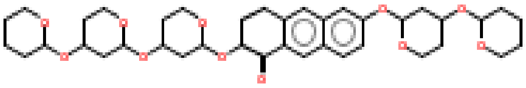	1	2 (2/0)	3 (3/0)
DS46 (22)	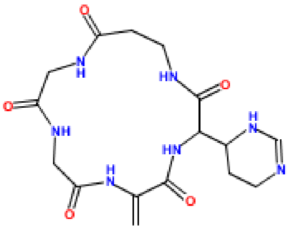	1	2 (2/0)	2 (2/0)

DS47 (23)	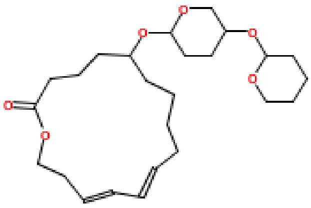	1	2 (2/1)	5 (4/1)
DS48 (24)	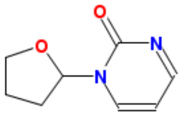	1	4 (4/0)	7 (7/0)
DS49 (25)	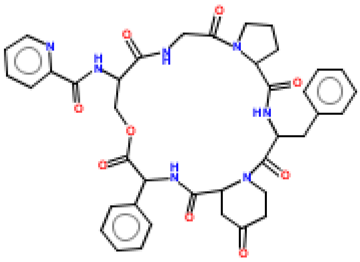	1	2 (2/0)	2 (2/0)
DS50 (26)	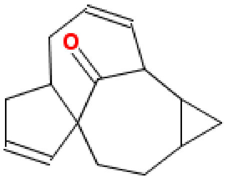	1	2 (1/1)	2 (1/1)
DS51 (27)	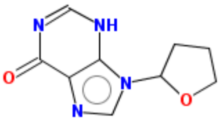	1	3 (3/0)	7 (7/0)
DS52 (28)	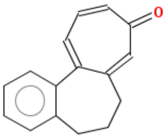	1	2 (2/0)	2 (2/0)
DS53 (29)	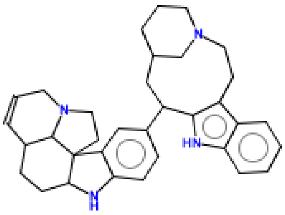	1	2 (2/1)	6 (4/2)

DS54 (30)	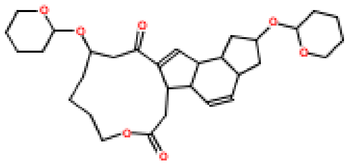	1	2 (2/0)	1 (1/0)
DS55 (31)	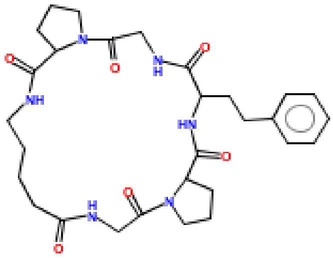	1	3 (2/1)	4 (3/1)
DS56 (32)	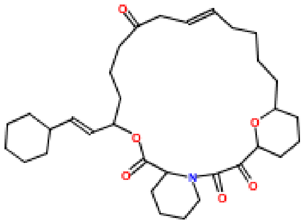	1	2 (2/0)	2 (2/0)
DS57 (33)	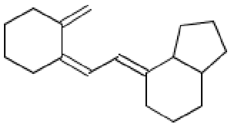	1	2 (2/0)	7 (7/0)
DS58 (34)	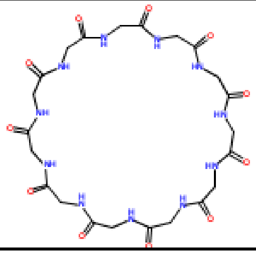	1	2 (1/2)	6 (2/4)
DS59 (35)	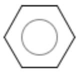	1	21 (18/5)	51 (43/8)
DS60 (36)	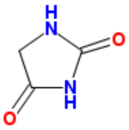	1	2 (1/1)	2 (1/1)
DS61 (37)	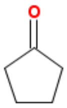	1	2 (2/0)	12 (12/0)

DS62 (38)	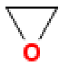	1	2 (2/1)	4 (3/1)

Supplementary Table S5 Statistics of the 33 main branches of the molecular-fingerprint Tanimoto-coefficient similarity clustering tree of the natural product chemical space represented by 137,836 natural products and 442 natural product leads. These branches are labeled as Branch1 to Branch33.

Main Branch	No of Natural Products	No of Drug Leads (Approved/Clinical Trial)	No of Derived Drugs (Approved/Clinical Trial)
Branch1	801	13 (12/2)	17 (15/2)
Branch2	329	1 (1/0)	1 (1/0)
Branch3	3937	72 (59/18)	214 (186/28)
Branch4	848	29 (25/6)	84 (53/31)
Branch5	809	0 (0/0)	0 (0/0)
Branch6	1813	2 (1/1)	3 (2/1)
Branch7	9553	9 (9/1)	36 (20/16)
Branch8	1617	3 (2/1)	5 (4/1)
Branch9	4594	65 (55/17)	161 (124/37)
Branch10	3224	22 (18/5)	43 (38/5)
Branch11	1408	5 (5/0)	5 (5/0)
Branch12	7837	29 (24/7)	51 (39/12)
Branch13	2286	5 (5/1)	14 (12/2)
Branch14	5966	27 (17/11)	42 (28/14)
Branch15	9746	16 (6/11)	19 (7/12)
Branch16	3763	14 (12/4)	48 (42/6)
Branch17	7323	25 (17/10)	50 (30/20)
Branch18	3868	3 (2/1)	3 (2/1)
Branch19	1136	0 (0/0)	0 (0/0)
Branch20	9754	23 (19/7)	72 (50/22)
Branch21	2858	7 (7/1)	11 (9/2)
Branch22	8360	7 (2/5)	11 (2/9)
Branch23	164	0 (0/0)	0 (0/0)
Branch24	6264	16 (14/6)	33 (26/7)
Branch25	8973	1 (0/1)	1 (0/1)
Branch26	1262	8 (7/3)	21 (14/7)
Branch27	2027	3 (2/1)	3 (2/1)
Branch28	6496	12 (8/4)	21 (13/8)
Branch29	643	0 (0/0)	0 (0/0)
Branch30	3457	4 (2/3)	8 (3/5)
Branch31	3879	1 (1/0)	1 (1/0)
Branch32	8696	16 (13/6)	37 (24/13)
Branch33	4145	4 (3/2)	5 (3/2)

Supplementary Table S6 Statistics of the 60 drug lead productive clusters in the molecular-fingerprint Tanimoto-coefficient similarity clustering tree of the natural product chemical space represented by 137,836 natural products and 442 natural product leads. These clusters are labeled as DC1 to DC60.

Drug Lead Productive Cluster (Main Branch)	No of NPs	No of Drug Leads (Approved/Clinical Trial)	No of Derived Drugs (Approved/Clinical Trial)	Drug Lead Molecular Scaffold Groups	Target Site SuperClass (TS)	TS Id	Target Site Class (TC)	TC Id
DC1 (1)	11	2 (2/0)	3 (3/0)	Uracil analogs	nucleobase binding sites	TS2	Pyrimidine metabolism enzymes substrate binding sites	TC3
DC2 (1)	4	2 (2/0)	2 (2/0)	Tuberactinomycin family peptides	aminoacyl-tRNA binding sites	TS6	ribosome 30s subunit aminoacyl-tRNA binding sites	TC18
DC3 (1)	28	2 (2/0)	3 (3/0)	Sulfur-containing amino acids	fatty acid, cannabinoid, eicosanoid, retinoid binding sites	TS15	fatty acid metabolism enzyme substrate sites	TC36
DC4 (3)	225	4 (4/2)	14 (12/2)	linear derivatives of amino acids with very short acyclic hydroxyl side chain	oligopeptide binding sites	TS9	serine endopeptidase substrate binding sites	TC28
							proteasome substrate binding sites	TC27
DC5 (3)	225	19 (19/0)	32 (32/0)	Pyrimidine nucleoside analogs, Aminoglycosides, Peptidoglycans	nucleoside phosphate binding sites	TS4	DNA metabolism enzymes nucleoside phosphate binding sites	TC7
					aminoacyl-tRNA binding sites	TS6	ribosome 30s subunit aminoacyl-tRNA binding sites	TC18
DC6 (3)	197	5 (3/3)	8 (4/4)	Imino sugars	aminoacyl-tRNA binding sites	TS6	ribosome 30s subunit aminoacyl-tRNA binding sites	TC18
					saccharide binding sites	TS12	monosaccharide metabolism enzyme binding sites	TC31
DC7 (3)	217	15 (15/0)	23 (23/0)	Amino acids with acyclic hydroxyl side chain & derivatives	amino acid binding sites	TS8	amino acid receptors ligand binding sites	TC20
							amino acid metabolism enzymes substrates binding sites	TC21
DC8 (3)	92	6 (6/2)	96 (90/6)	Beta-lactams	peptidoglycan binding sites	TS10	ß-lactam binding protein peptidoglycan binding sites	TC29
DC9 (3)	258	7 (3/4)	8 (4/4)	Larger oligopeptides	amino acid binding sites	TS8	amino acid receptors ligand binding sites	TC20
					oligopeptide binding sites	TS9	proteasome substrate binding sites	TC27
DC10 (3)	237	5 (5/0)	17 (17/0)	Glycosaminoglycans, glucosamines, Lincosamides & derivatives	oligopeptide binding sites	TS9	serine endopeptidase substrate binding sites	TC28
DC11 (4)	67	4 (1/4)	10 (1/9)	Polyketide macrolactones, Carbapenems, Sulfonamides	microtubule sites	TS17	microtubule laulimalide/peloruside site	TC41
DC12 (4)	180	5 (5/0)	9 (9/0)	Purine base analogs, modified purine base analogs	nucleoside binding sites	TS3	purine nucleoside receptor ligand binding sites	TC5
					nucleoside phosphate	TS4	DNA metabolism enzymes	TC7

					binding sites		nucleoside phosphate binding sites	
DC13 (4)	264	18 (17/2)	61 (39/22)	Purine nucleoside analogs, Imidazole analogs, Imidazole oligopeptide hybrids	nucleoside binding sites	TS3	purine nucleoside receptor ligand binding sites	TC5
							purine nucleoside metabolism enzyme substrate binding sites	TC6
					nucleoside phosphate binding sites	TS4	nucleoside phosphate receptor ligand binding sites	TC9
							DNA metabolism enzymes nucleoside phosphate binding sites	TC7
DC14 (7)	410 4	5 (5/1)	31 (15/16)	Larger indole alkaloids	amine binding sites	TS1	amine receptors ligand binding sites	TC1
							amine transporters substrate binding sites	TC2
					oligopeptide binding sites	TS9	exopeptidase substrate binding sites	TC23
DC15 (12)	71	3 (2/1)	5 (4/1)	Mono-, Sesqui-, Di-terpenes with simple ring scaffolds	fatty acid, cannabinoid, eicosanoid, retinoid binding sites	TS15	retinoid receptor ligand binding sites	TC37
DC16 (13)	11	3 (2/3)	9 (3/6)	Small cyclic peptides	amino acid phosphate binding sites	TS7	phosphatase substrate binding sites	TC19
					oligopeptide binding sites	TS9	oligopeptide histone tail metabolism enzymes substrate binding sites	TC24
DC17 (14)	117	16 (15/4)	43 (29/14)	Macrolides, Polyenes, Spinosyns, Acarviosins	amino acid phosphate binding sites	TS7	phosphatase substrate binding sites	TC19
					oligopeptide binding sites	TS9	ribosome 23S rRNA peptidyl transferase sites	TC25
					lipopolysaccharide sites	TS14	outer membrane lipopolysaccharide sites	TC35
DC18 (14)	217	2 (2/0)	2 (2/0)	Steroidal alkaloids, Steroidal glycoalkaloids	steroid binding sites	TS19	Nuclear receptor ligand binding sites	TC44
DC19 (14)	546	37 (32/5)	93 (85/8)	Steroids & derivatives	steroid binding sites	TS19	Nuclear receptor ligand binding sites	TC44
DC20 (14)	34	2 (2/0)	2 (2/0)	Leukotrienes				
DC21 (14)	423	9 (8/1)	27 (26/1)	Fatty acids & derivatives, Prostanoids	fatty acid, cannabinoid, eicosanoid, retinoid binding sites	TS15	retinoid receptor ligand binding sites	TC37
					coenzyme A & analog binding sites	TS16	coenzyme A & analog metabolism enzymes substrate binding sites	TC40
DC22 (15)	185	3 (2/1)	3 (2/1)	Sesquiterpene lactones. Meroterpenoids	oligopeptide binding sites	TS9	exopeptidase substrate binding sites	TC23
DC23 (16)	782	2 (1/1)	2 (1/1)	Lone-tailed phosphate esters, Long tailed polyketide-derived acids	nucleoside phosphate binding sites	TS4	DNA metabolism enzymes nucleoside phosphate binding sites	TC7
							RNA metabolism enzymes nucleoside phosphate binding sites	TC8
DC24 (16)	259	5 (5/0)	5 (5/0)	Oligo-, Poly-, Cyclic-saccharides	cyclic oligosaccharide drug delivery systems	TS13	cyclodextrin drug delivery systems	TC34
DC25 (17)	3	2 (2/0)	7 (7/0)	Secosteroids	steroid binding site	TS19	Nuclear receptor ligand binding sites	TC44
DC26 (17)	9	2 (2/0)	7 (7/0)	Statins	coenzyme A & analog binding sites	TS16	coenzyme A & analog metabolism enzymes substrate binding sites	TC40

DC27 (17)	139 4	8 (4/5)	13 (4/9)	Sesqui-, Di-terpenes with steroid-like multi-ring scaffolds	nucleoside phosphate binding sites	TS4	heat shock protein nucleoside phosphate binding sites	TC16
					oligopeptide binding sites	TS9	ribosome 23S rRNA peptidyl transferase sites	TC25
DC28 (17)	176	8 (8/1)	13 (12/1)	Cardiac glycosides	nucleoside phosphate binding sites	TS4	nucleoside phosphate metabolism enzymes substrate binding sites	TC10
DC29 (20)	169 6	7 (6/1)	9 (7/2)	Saponins, Triterpenoid glycosides, Macrocyclic lactones	nucleoside phosphate binding sites	TS4	steroid metabolism enzyme nucleoside phosphates binding sites	TC12
							calcium channel DHP binding site	TC14
							chloride channel CBS domain	TC15
DC30 (20)	30	2 (2/0)	2 (2/0)	ß-triketones , Cyclic ketones of the hydroaromatic terpene group				
DC31 (8)	399	5 (4/1)	9 (8/1)	Phenethylamines & Benzylamines with a shorter side-chain, Chalconoids	amine binding sites	TS1	amine receptors ligand binding sites	TC1
							amine transporters substrate binding sites	TC2
DC32 (9)	334	5 (3/2)	6 (4/2)	Pyridine alkaloids	nucleoside phosphate binding sites	TS4	ATP-gated channel ligand binding sites	TC13
DC33 (9)	393	4 (4/1)	16 (13/3)	Nitrobenzene analogs, benzene-1,4-dicarboxylic acids & ß-triketones with an intermediate side-chain	nucleoside phosphate binding sites	TS4	ATP-gated channel ligand binding sites	TC13
					oligopeptide binding sites	TS9	ribosome 23S rRNA peptidyl transferase sites	TC25
DC34 (9)	331	3 (2/1)	3 (2/1)	Phenethylamines with a longer side-chain	oligopeptide binding sites	TS9	exopeptidase substrate binding sites	TC23
DC35 (9)	67	6 (2/4)	8 (2/6)	Depsipeptides, Cyclotetrapeptides, Nonapeptides & analogs	oligopeptide binding sites	TS9	oligopeptide histone tail metabolism enzymes substrate binding sites	TC24
							vasoactive peptide receptor binding sites	TC26
					Sites within peptidoglycans	TS11	cell wall peptidoglycan sites	TC30
DC36 (20)	265	10 (5/6)	13 (6/7)	Large cyclic peptides	nucleoside phosphate binding sites	TS4	calcium channel DHP binding site	TC14
					Sites within peptidoglycans	TS11	cell wall peptidoglycan sites	TC30
					saccharide binding sites	TS12	polysaccharide metabolism enzyme substrates binding sites	TC33
					lipopolysaccharide sites	TS14	outer membrane lipopolysaccharide sites	TC35
DC37 (21)	135	3 (3/1)	5 (3/2)	Streptogramins	oligopeptide binding sites	TS9	ribosome 23S rRNA peptidyl transferase sites	TC25
DC38 (21)	33	7 (7/1)	34 (32/2)	Intermediate-sized linear and cyclic peptides	oligopeptide binding sites	TS9	exopeptidase substrate binding sites	TC23
							Neuropeptide receptor ligand binding sites	TC22
					lipopolysaccharide sites	TS14	outer membrane lipopolysaccharide sites	TC35
DC39 (24)	7	3 (1/3)	13 (3/10)	Staurosporine alkaloids	nucleoside phosphate binding sites	TS4	kinase ATP binding sites	TC11
							DNA metabolism enzymes nucleoside phosphate binding sites	TC7
DC40	519	7 (5/3)	12 (8/4)	Porphyrins, Reduced	amine binding sites	TS1	amine receptors ligand binding	TC1

(24)				porphyrins, Prodiginines, Ergoline-, Ellipticine-, Epibatidine- alkaloids			sites	
					nucleobase binding sites	TS2	DNA intercalation sites	TC4
DC41 (26)	78	2 (1/1)	3 (2/1)	Smaller indole alkaloids	amine binding sites	TS1	amine receptors ligand binding sites	TC1
DC42 (26)	512	7 (5/2)	10 (6/4)	Indole-containing amino acid tryptophan analogs, Monoterpenoid indole alkaloids, Yohimbine alkaloids	amine binding sites	TS1	amine receptors ligand binding sites	TC1
DC43 (28)	29	5 (5/0)	10 (10/0)	Tropane alkaloids	amine binding sites	TS1	amine receptors ligand binding sites	TC1
							amine transporters substrate binding sites	TC2
DC44 (28)	358	5 (5/0)	21 (21/0)	Catecholamines, Small alkaloids with an amine group	amine binding sites	TS1	amine receptors ligand binding sites	TC1
					opiate binding sites	TS18	opiate receptor ligand binding sites	TC43
DC45 (30)	447	7 (6/3)	20 (13/7)	Tetracyclines, Capsaicinoids, Disulfide bromotyrosine derivatives	aminoacyl-tRNA binding sites	TS6	ribosome 30s subunit aminoacyl-tRNA binding sites	TC18
DC46 (32)	20	3 (3/1)	7 (5/2)	Vinca alkaloids	microtubule sites	TS17	microtubule taxoid site	TC42
DC47 (33)	173	4 (4/0)	4 (4/0)	Protoberberine alkaloids, Benzylisoquinoline alkaloids	nucleoside phosphate binding sites	TS4	nucleoside phosphate metabolism enzymes substrate binding sites	TC10
					cyclic nucleotide binding site	TS5	cyclic nucleotide phosphodiesterase substrate binding sites	TC17
					amino acid phosphate binding sites	TS7	phosphatase substrate binding sites	TC19
DC48 (32)	943	4 (4/1)	5 (4/1)	Colchicine alkaloids, Phthalideisoquinoline alkaloids	microtubule sites	TS17	microtubule taxoid site	TC42
DC49 (32)	457	6 (6/1)	19 (18/1)	Opiate alkaloids, Phenanthrene alkaloids	amine binding sites	TS1	amine receptors ligand binding sites	TC1
					opiate binding sites	TS18	opiate receptor ligand binding sites	TC43
DC50 (32)	97	5 (4/2)	15 (10/5)	Anthracyclines, Aminocoumarins, Coumarin-related chartarin chromophore analogs	nucleobase binding sites	TS2	DNA intercalation sites	TC4
					nucleoside phosphate binding sites	TS4	DNA metabolism enzymes nucleoside phosphate binding sites	TC7
DC51 (32)	27	2 (2/1)	5 (3/2)	Calicheamicins, Enediynes				
DC52 (10)	663	3 (3/0)	4 (4/0)	Small phenolic molecules	fatty acid, cannabinoid, eicosanoid, retinoid binding sites	TS15	retinoid receptor ligand binding sites	TC37
							eicosanoid metabolism enzyme substrate sites	TC38
DC53 (10)	2531	9 (5/4)	17 (9/8)	Cannabinoids, Small phenolic molecules with a long tail	fatty acid, cannabinoid, eicosanoid, retinoid binding sites	TS15	fatty acid metabolism enzyme substrate sites	TC36
							retinoid receptor ligand binding sites	TC37
							cannabinoid receptor ligand binding sites	TC39

DC54 (10)	349	2 (2/1)	6 (3/3)	Chromone derivatives	naphthoquinone binding sites	TS20	naphthoquinone binding protein naphthoquinone binding sites	TC45
DC55 (10)	472	3 (3/0)	5 (5/0)	Anthraquinones & derivatives	nucleobase binding sites	TS2	DNA intercalation sites	TC4
DC56 (11)	222	2 (1/1)	3 (2/1)	Cannabinoids, Benzofuran derivatives	fatty acid, cannabinoid, eicosanoid, retinoid binding sites	TS15	cannabinoid receptor ligand binding sites	TC39
DC57 (12)	174	2 (2/0)	3 (3/0)	Flavanone glycosides, Flavonol rutinose				
DC58 (12)	105	2 (2/0)	5 (5/0)	Furanochromones				
DC59 (12)	500	3 (3/2)	5 (3/2)	Flavanones	fatty acid, cannabinoid, eicosanoid, retinoid binding sites	TS15	fatty acid metabolism enzyme substrate sites	TC36
DC60 (12)	32	2 (1/1)	2 (1/1)	Phenylpropanoids & derivatives	saccharide binding sites	TS12	monosaccharide receptor binding sites	TC32

Supplementary Table S7 List of 20 target site superclasses and their corresponding target site classes with examples. The target site superclasses are labeled as TS1-TS20 and the target site classes are labeled as TC1-TC45.

Target Site Superclass		Target Site Class		Example of Targets
TS1	amine binding sites	TC1	amine receptors ligand binding sites	Dopamine receptors, Histamine receptors, 5HT-receptors
		TC2	amine transporters substrate binding sites	Serotonin reuptakes, Dopamine reuptakes, Norepinephrine reuptakes
TS2	nucleobase binding sites	TC3	Pyrimidine metabolism enzymes substrate binding sites	Thymidylate synthase
		TC4	DNA intercalation sites	DNA
TS3	nucleoside binding sites	TC5	purine nucleoside receptor ligand binding sites	Adenosine receptor
		TC6	purine nucleoside metabolism enzyme substrate binding sites	Adenosine deaminase
TS4	nucleoside phosphate binding sites	TC7	DNA metabolism enzymes nucleoside phosphate binding sites	DNA topoisomerases, DNA polymerases
		TC8	RNA metabolism enzymes nucleoside phosphate binding sites	t-RNA synthetases
		TC9	nucleoside phosphate receptor ligand binding sites	Adenosine diphosphate receptors
		TC10	nucleoside phosphate metabolism enzymes substrate binding sites	Na+/K+-ATPase, Na+-k+-atpase transmembrane protein
		TC11	kinase ATP binding sites	Kinases
		TC12	steroid metabolism enzyme nucleoside phosphates binding sites	11β-hydroxysteroid dehydrogenase
		TC13	ATP-gated channel ligand binding sites	ATP-sensitive k+ channel, ATP-sensitive inward rectifier potassium channel
		TC14	calcium channel DHP binding site	Ca(2+) channels, N-type voltage-dependent calcium channels
		TC15	chloride channel CBS domain	Glutamate-gated chloride channel
		TC16	heat shock protein nucleoside phosphate binding sites	HSP70
TS5	cyclic nucleotide binding sites	TC17	cyclic nucleotide phosphodiesterase substrate binding sites	cGMP-specific 3',5'-cyclic phosphodiesterase
TS6	aminoacyl-tRNA binding sites	TC18	ribosome 30s subunit aminoacyl-tRNA binding sites	30s ribosomal subunit
TS7	amino acid phosphate binding sites	TC19	phosphatase substrate binding sites	Calcineurin, protein tyrosine phosphatase 1B
TS8	amino acid binding sites	TC20	amino acid receptors ligand binding sites	Glutamine receptors, NMDA receptor
		TC21	amino acid metabolism enzymes substrates binding sites	GABA-transaminase
TS9	oligopeptide binding sites	TC22	Neuropeptide receptor ligand binding sites	Gonadotropin-releasing hormone receptor, Somatostatin receptor
		TC23	exopeptidase substrate binding sites	Aminopeptidase, dipeptidyl peptidase-4, Angiotensin-converting enzyme
		TC24	oligopeptide histone tail metabolism enzymes substrate binding sites	HDAC

		TC25	ribosome 23S rRNA peptidyl transferase sites	23s rRNA
		TC26	vasoactive peptide receptor binding sites	Bradykinin b2 receptor
		TC27	proteasome substrate binding sites	20s proteasome
		TC28	serine endopeptidase substrate binding sites	HIV-1 protease, Factor Xa
TS10	peptidoglycan binding sites	TC29	β-lactam binding protein peptidoglycan binding sites	Penicillin binding proteins
TS11	Sites within peptidoglycans	TC30	cell wall peptidoglycan sites	Bacterial outer membrane
		TC31	monosaccharide metabolism enzyme binding sites	Ceramide glucosyltransferase
		TC32	monosaccharide receptor binding sites	sweet taste receptor
TS12	saccharide binding sites	TC33	polysaccharide metabolism enzyme substrates binding sites	1,3-β-D-glucan synthase
TS13	cyclic oligosaccharide drug delivery systems	TC34	cyclodextrin drug delivery systems	cyclodextrin drug delivery systems
TS14	lipopolysaccharide sites	TC35	outer membrane lipopolysaccharide sites	Bacterial and fungal outer membrane
		TC36	fatty acid metabolism enzyme substrate sites	Carnitine o-palmitoyltransferase, Diglyceride acyltransferase, lipoxygenases
		TC37	retinoid receptor ligand binding sites	Retinoic acid receptors, Retinoid X receptor,
		TC38	eicosanoid metabolism enzyme substrate sites	Cox2
TS15	fatty acid, cannabinoid, eicosanoid, retinoid binding sites	TC39	cannabinoid receptor ligand binding sites	Cannabinoid receptors
TS16	coenzyme A & analog binding sites	TC40	coenzyme A & analog metabolism enzymes substrate binding sites	Serine palmitoyltransferase
		TC41	microtubule laulimalide/peloruside site	Microtubule
TS17	microtubule sites	TC42	microtubule taxoid site	Microtubule
TS18	opiate binding sites	TC43	opiate receptor ligand binding sites	Opioid receptors
TS19	steroid binding sites	TC44	Nuclear receptor ligand binding sites	Estrogen receptor, Progesterone receptor
TS20	naphthoquinone binding sites	TC45	naphthoquinone binding protein naphthoquinone binding sites	Vitamin k epoxide reductase

Supplementary Table S8 List of drugs targeting 27 “new” targets first successfully explored since 1990.

Target (Year of First Successful Exploration)	Drug (Year of Approval)
	Argatroban (1990)
	Lepirudin (1997)
	Hirulog (2000)
	Bivalirudin (2000)
	Desirudin (2003)
	Ximelagatran (2004)
Thrombin (1990)	Dabigatran (2010)
	Acarbose (1990)
	Voglibose (1994)
Maltase-glucoamylase (1990)	Miglitol (1998)
Ornithine decarboxylase (1990)	Eflornithine hcl (1990)
4-hydroxyphenylpyruvate dioxygenase (1991)	Nitisinone (1991)
	Finasteride (1992)
	Azelaic acid (1995)
	Dutaseride (2001)
5-alpha reductase (1992)	Dutasteride (2001)
	Losartan potassium (1994)
	Valsartan (1996)
	Eprosartan (1997)
	Irbesartan (1997)
	Candesartan cilexetil (1997)
	Telmisartan (1999)
Angiotensin receptor (1994)	Olmesartan medoxil (2002)
	Camptothecin (1994)
	Irinotecan hydrochloride (1994)
	Topotecan hcl (1996)
DNA topoisomerase I (1994)	Sphingosomal topotecan (2007)
	Saquinavir mesylate (1995)
	Ritonavir (1996)
	Indinavir sulfate (1996)
	Neflinavir mesylate (1997)
	Amprenavir (1999)
	Lopinavir (2000)
	Atazanavir (2003)
	Fosamprenavir (2003)
	Tipranavir (2005)
HIV-1 protease (1995)	Darunavir (2006)
	Zotarolimus (1996)
	Sirolimus (1999)
mTOR (1996)	Temsirolimus (2007)

	Everolimus (2009)
Histamine N-methyltransferase (1996)	Amodiaquine (1996)
	Latanoprost (1996)
	Travoprost (2001)
Prostaglandin f2-alpha receptor (1996)	Bimatoprost (2001)
	Oseltamivir phosphate (1999)
Neuraminidase (1999)	Zanamivir (1999)
Myeloid cell surface antigen cd33 (2000)	Gemtuzumab ozogamicin (2000)
Ferrochelatase (2001)	methyl aminolevulinate (2001)
Gamma-hydroxybutyrate receptor (2002)	Sodium oxybate (2002)
Ceramide glucosyltransferase (2003)	Miglustat (2003)
	Azacytidine (2004)
DNA (cytosine-5)-methyltransferase (2004)	Decitabine (2006)
Glutamine receptor (2005)	L-alanyl-l-glutamine (2005)
Methionine Aminopeptidase-2 (2005)	Fumagillin (2005)
	Vorinostat (2006)
Histone deacetylase (2006)	Romidepsin (2009)
Chloride channel protein 2 (2006)	Lubiprostone (2006)
Bradykinin B2 receptor (2008)	Icatibant (2008)
CYP17A1 (2009)	Abiraterone (2009)
Sphingosine 1-phosphate receptor (2010)	Fingolimod (2010)
	Crofelemer (2012)
Cystic fibrosis transmembrane conductance regulator (2012)	Ivacaftor (2012)
Somatostatin receptor 5 (2012)	Pasireotide diaspartate (2012)
Smoothened receptor (2012)	Vismodegib (2012)

Supplementary Figure S1 Distribution of the natural product leads of approved and clinical trial drugs in branches 1-4 of the molecular scaffold trees of the 134,097 natural products and 411 natural product leads. The drug-productive scaffolds or scaffold parent-child sub-branches (DSs) are indicated by red dots or red dots connected by red lines, which are marked by the respective label DS1, DS2, etc. The green triangles indicate the natural product leads which are outside the drug-productive scaffolds or scaffold parent-child sub-branches.

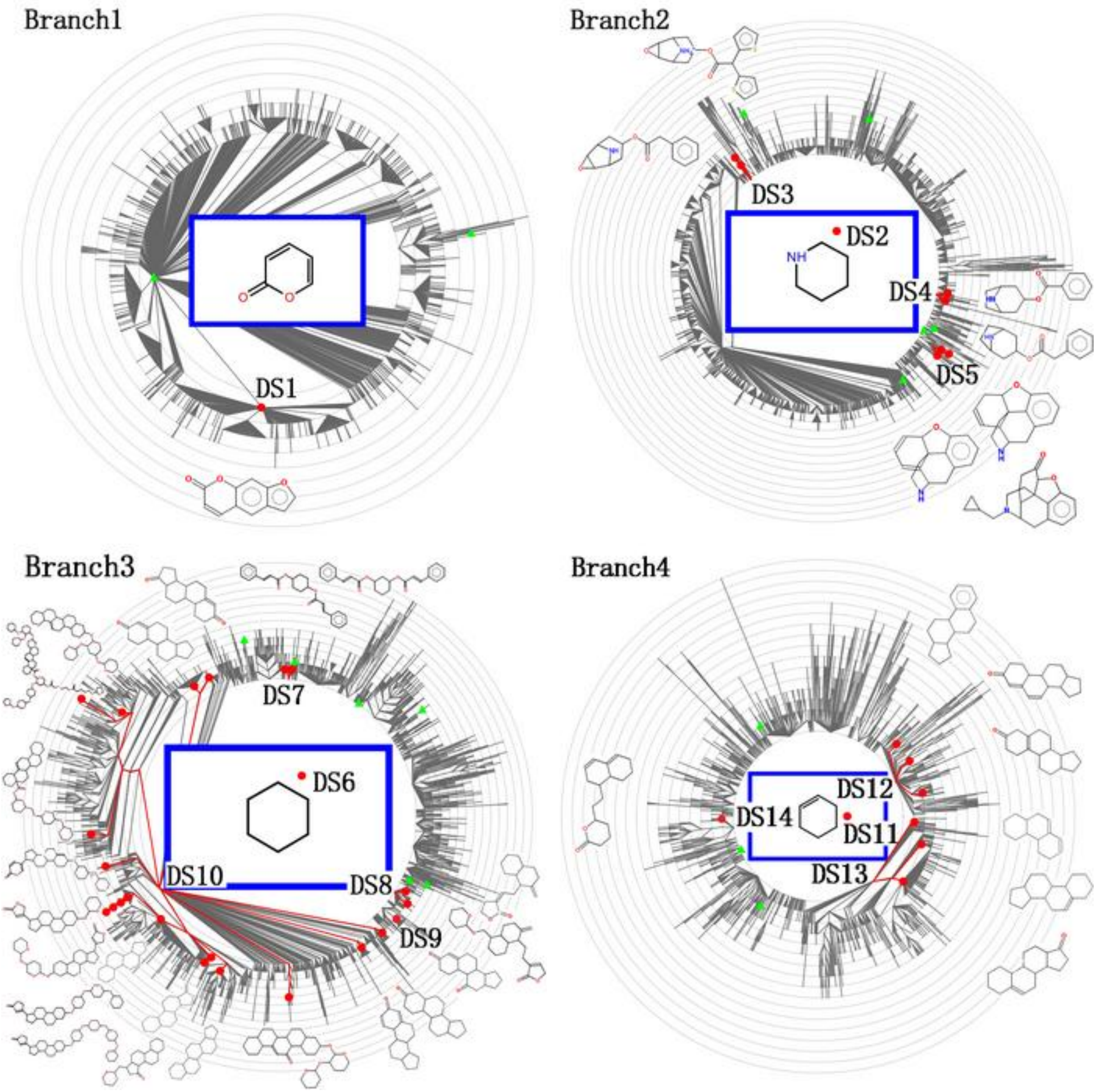

Supplementary Figure S2 Distribution of the natural product leads of approved and clinical trial drugs in branches 5-8 of the molecular scaffold trees of the 134,097 natural products and 411 natural product leads. The coloring and labeling schemes are the same as Supplementary Figure S1.

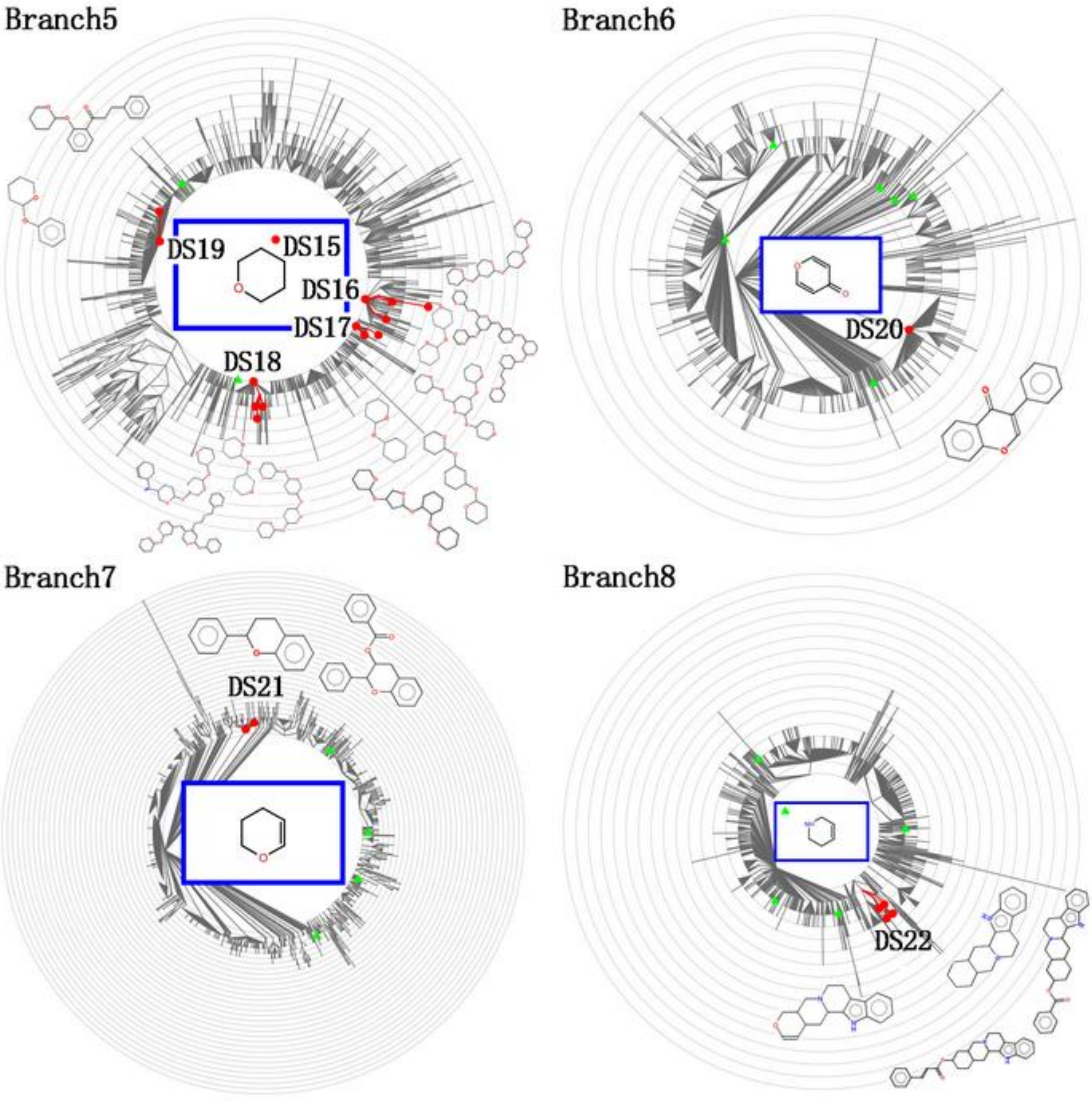

Supplementary Figure S3 Distribution of the natural product leads of approved and clinical trial drugs in branches 9-12 of the molecular scaffold trees of the 134,097 natural products and 411 natural product leads. The coloring and labeling schemes are the same as Supplementary Figure S1.

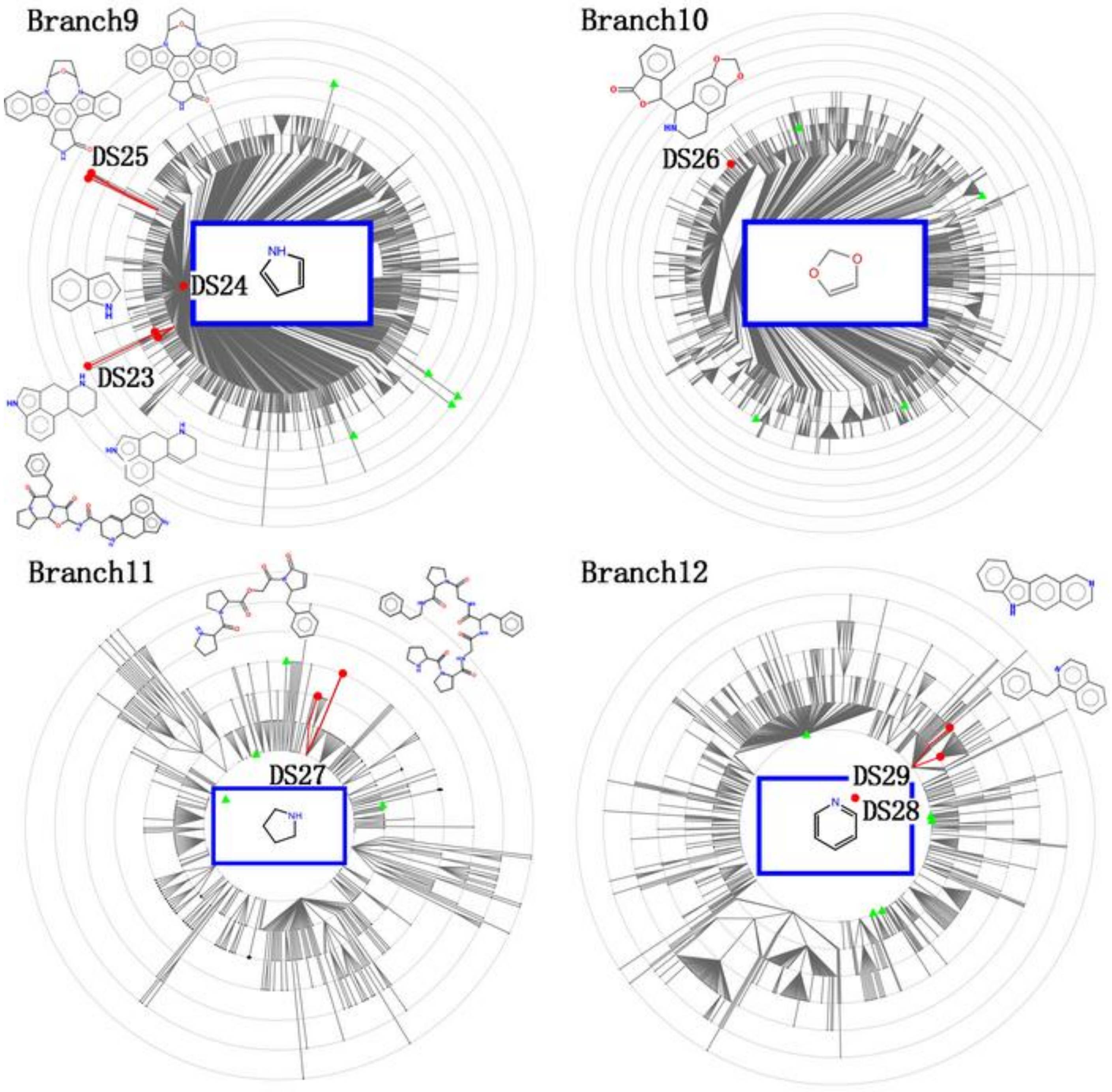

Supplementary Figure S4 Distribution of the natural product leads of approved and clinical trial drugs in branches 13-16 of the molecular scaffold trees of the 134,097 natural products and 411 natural product leads. The coloring and labeling schemes are the same as Supplementary Figure S1.

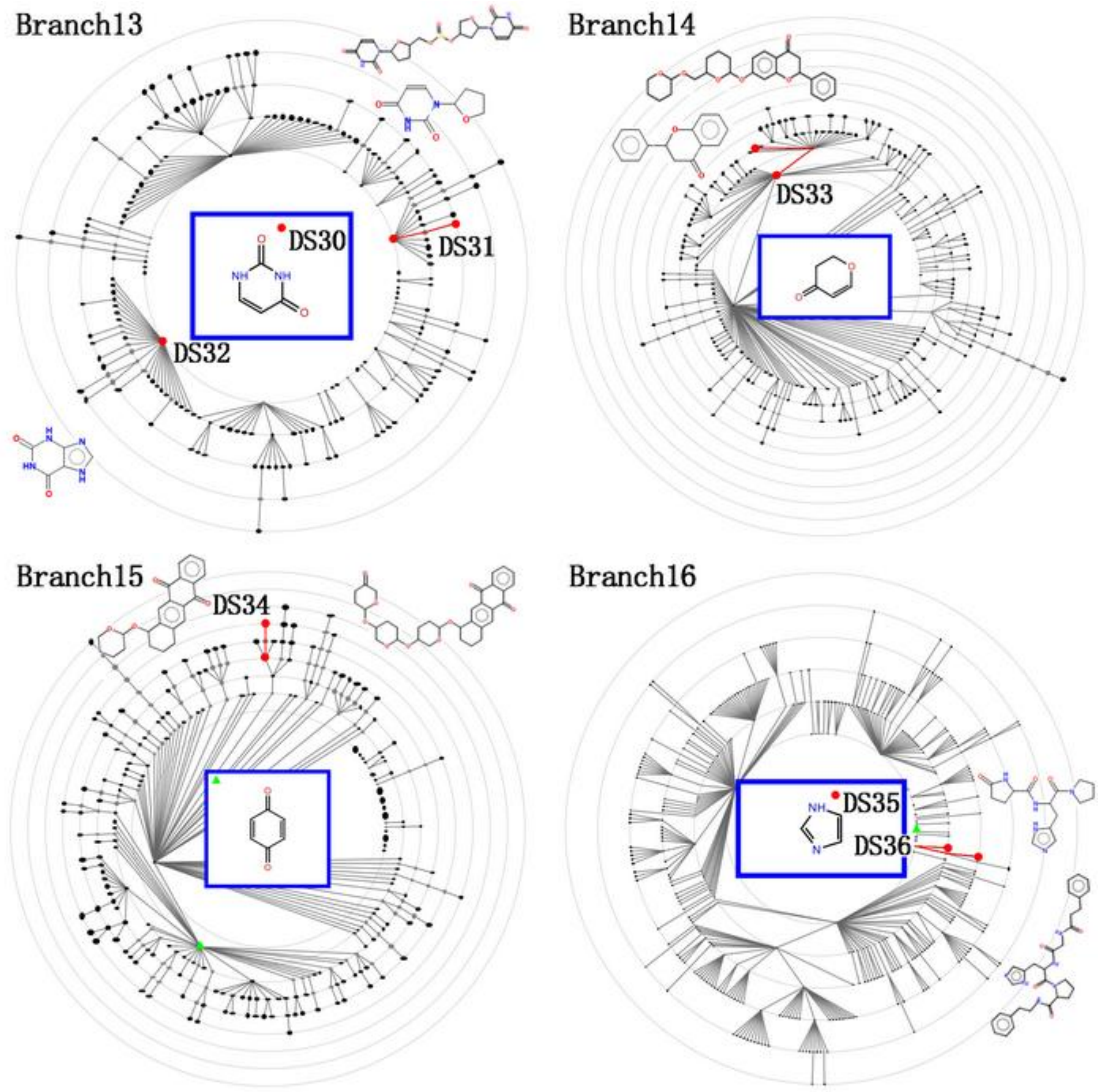

Supplementary Figure S5 Distribution of the natural product leads of approved and clinical trial drugs in branches 17-20 of the molecular scaffold trees of the 134,097 natural products and 411 natural product leads. The coloring and labeling schemes are the same as Supplementary Figure S1.

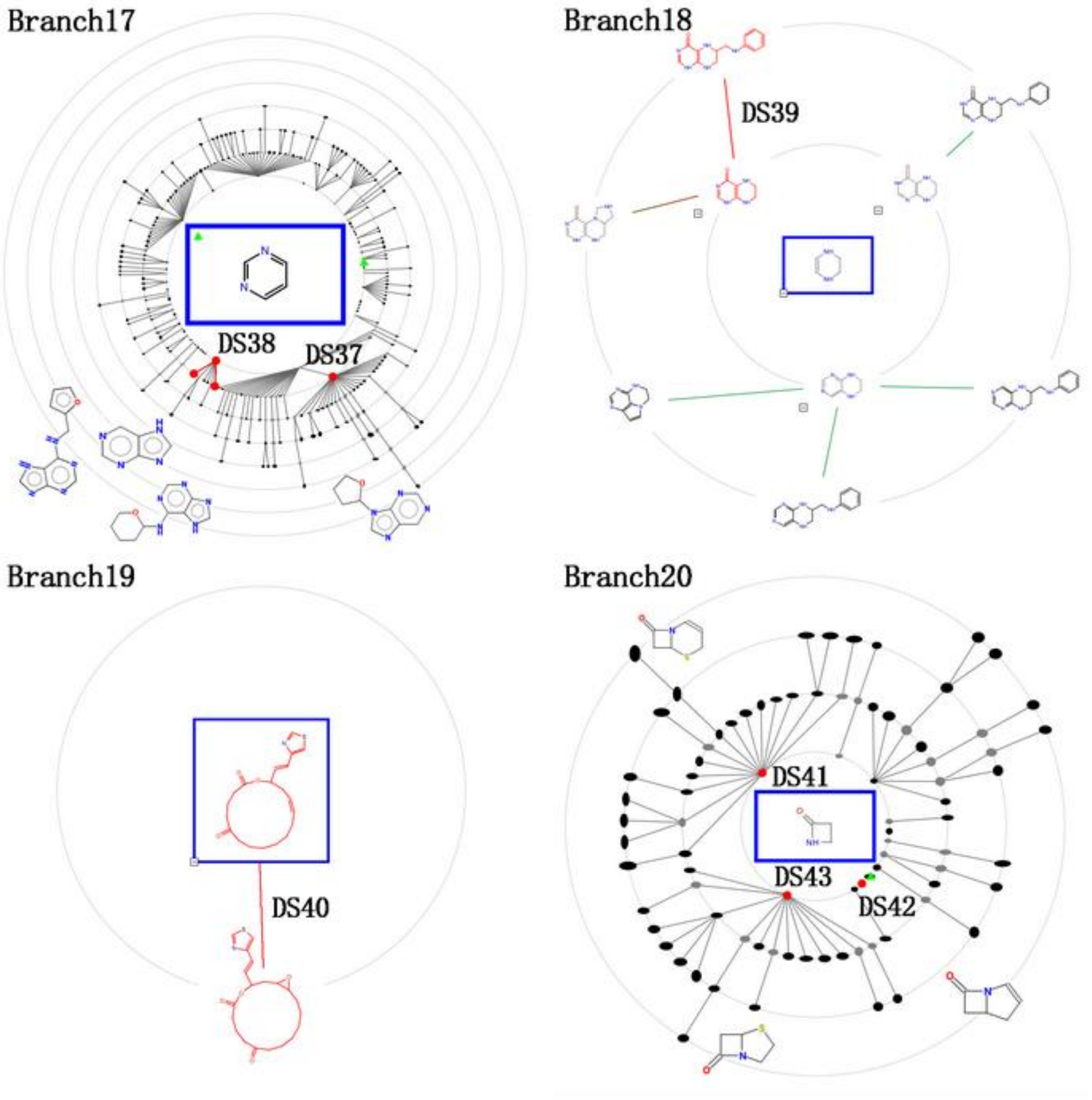

Supplementary Figure S6 The main branches of the MFTCS clustering tree of the 137,836 natural products and 442 natural product leads. The green digit near each branch-end is the number of natural products in that branch. The two red digits near the fruit beside each branch label box are the numbers of approved (proceed the + sign) and clinical trial leads in that branch. The fruit size roughly correlates with the number of leads. The representative molecular scaffold groups of each branch are given in the box beside each branch.

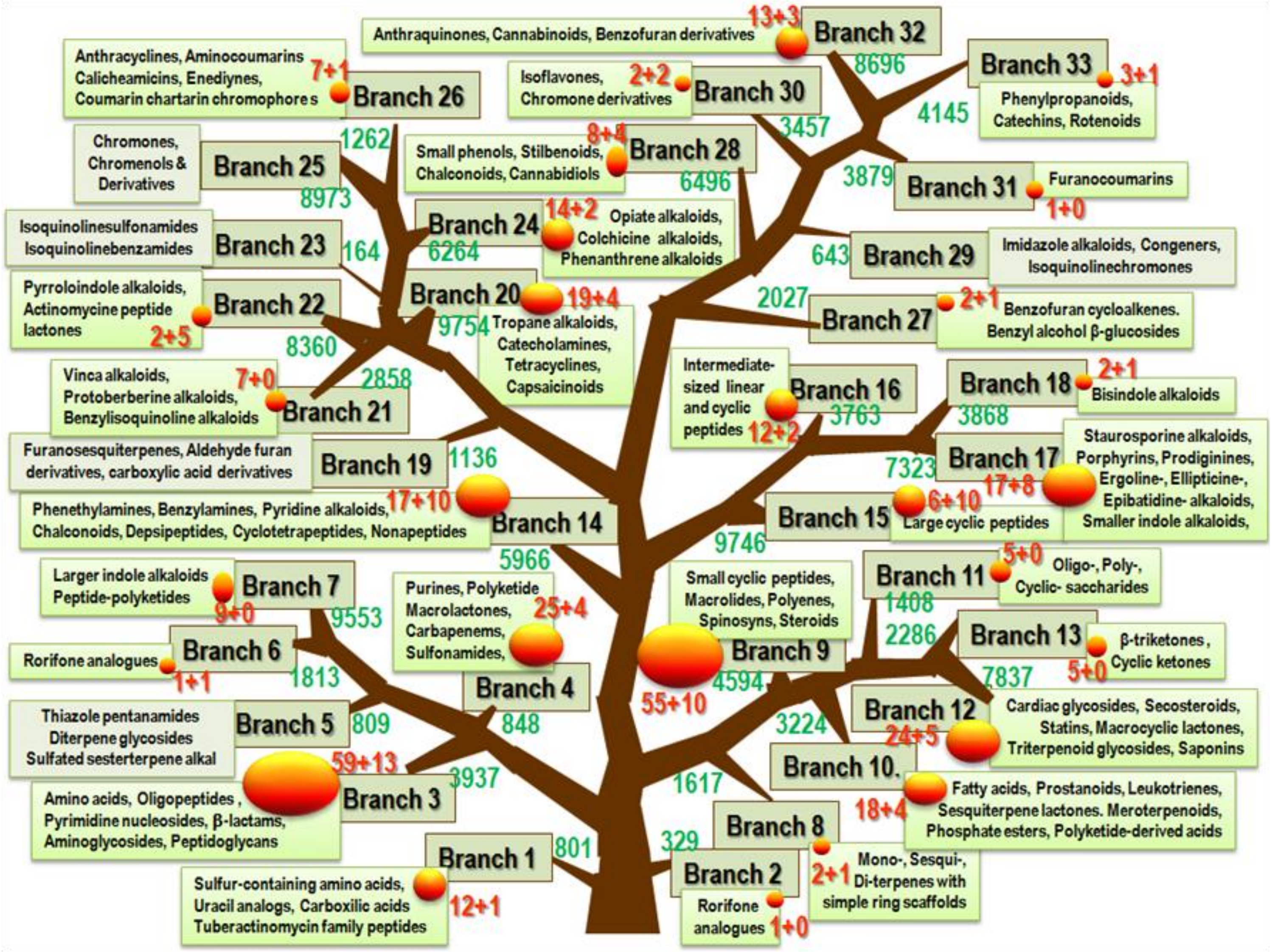

Supplementary Figure S7 Distribution of the natural product leads of approved and clinical trial drugs in branches 1-8 of the molecular-fingerprint Tanimoto-coefficient similarity clustering tree of the 137,836 natural products and 442 natural product leads. The drug-lead productive clusters are red-orange colored and marked by the respective cluster label. The red, purple and blue lines on top of the clustering tree indicate the locations of the approved, approved + clinical trial, and clinical trial drug-leads with the height correlating with the number of approved + clinical trial drugs.

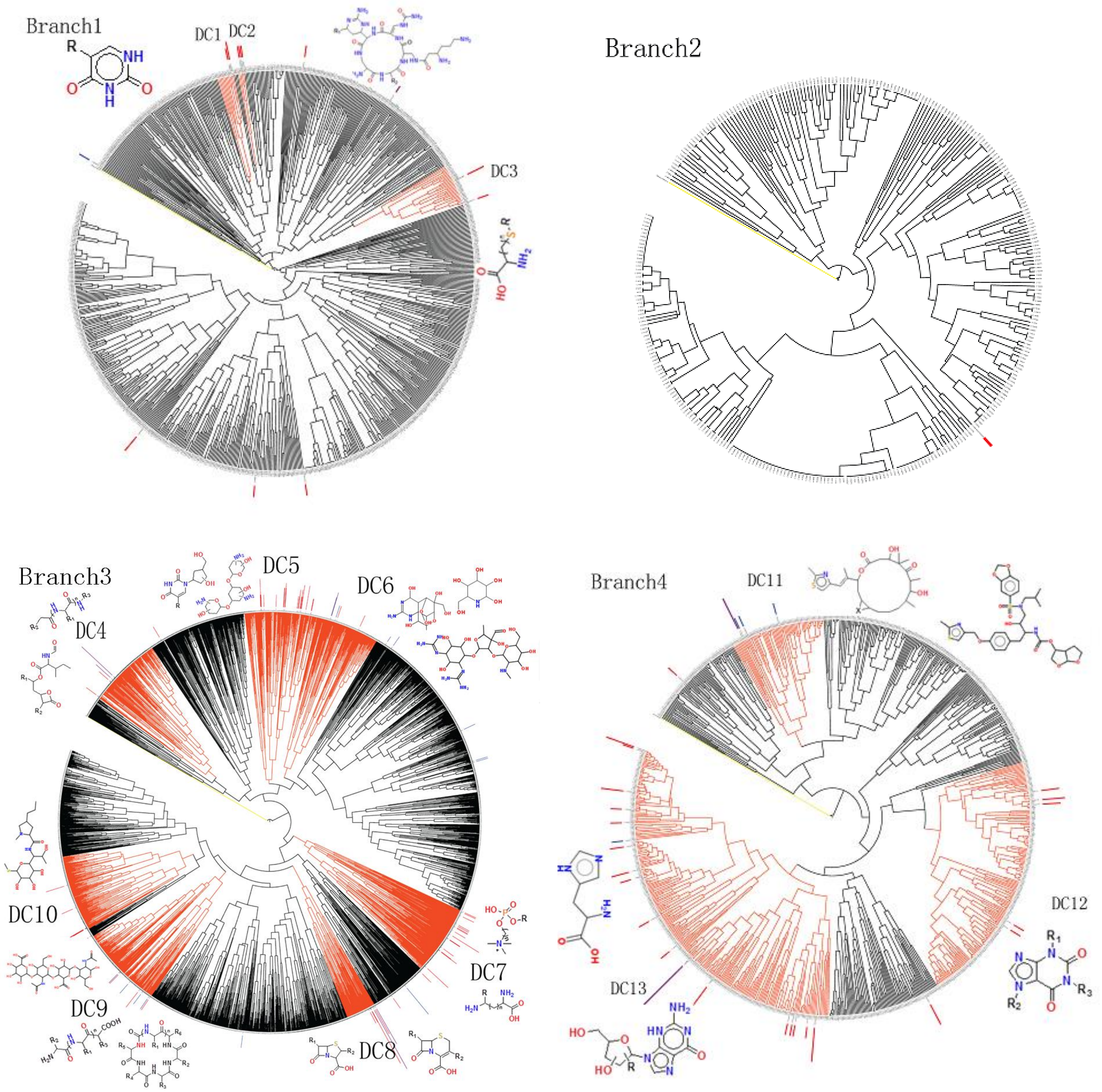

Branch5

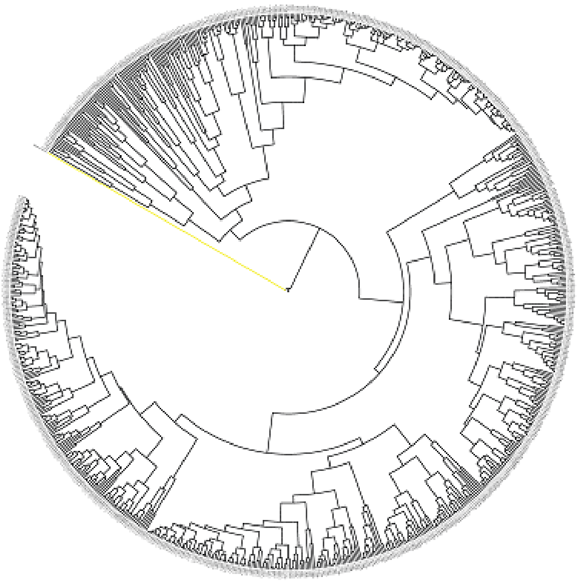

Branch6

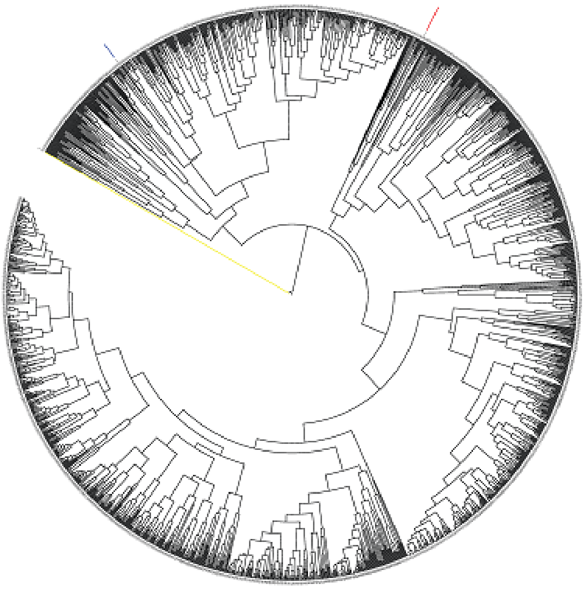

Branch7

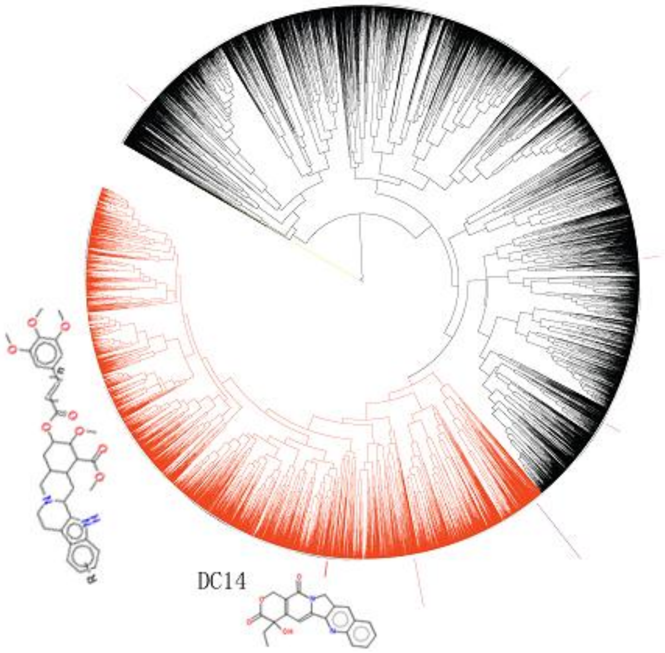

Branch8

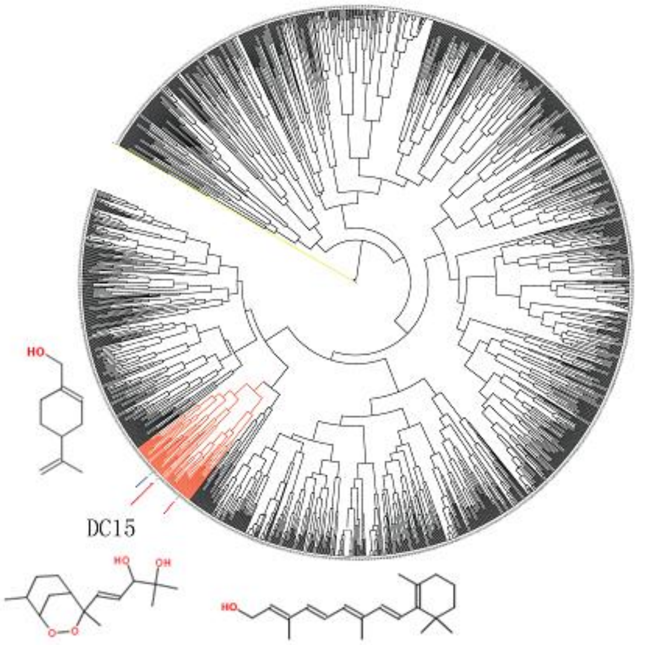

Supplementary Figure S8 Distribution of the natural product leads of approved and clinical trial drugs in branches 9-16 of the molecular-fingerprint Tanimoto-coefficient similarity clustering tree of the 137,836 natural products and 442 natural product leads. The coloring and labeling schemes are the same as Supplementary Figure S7.

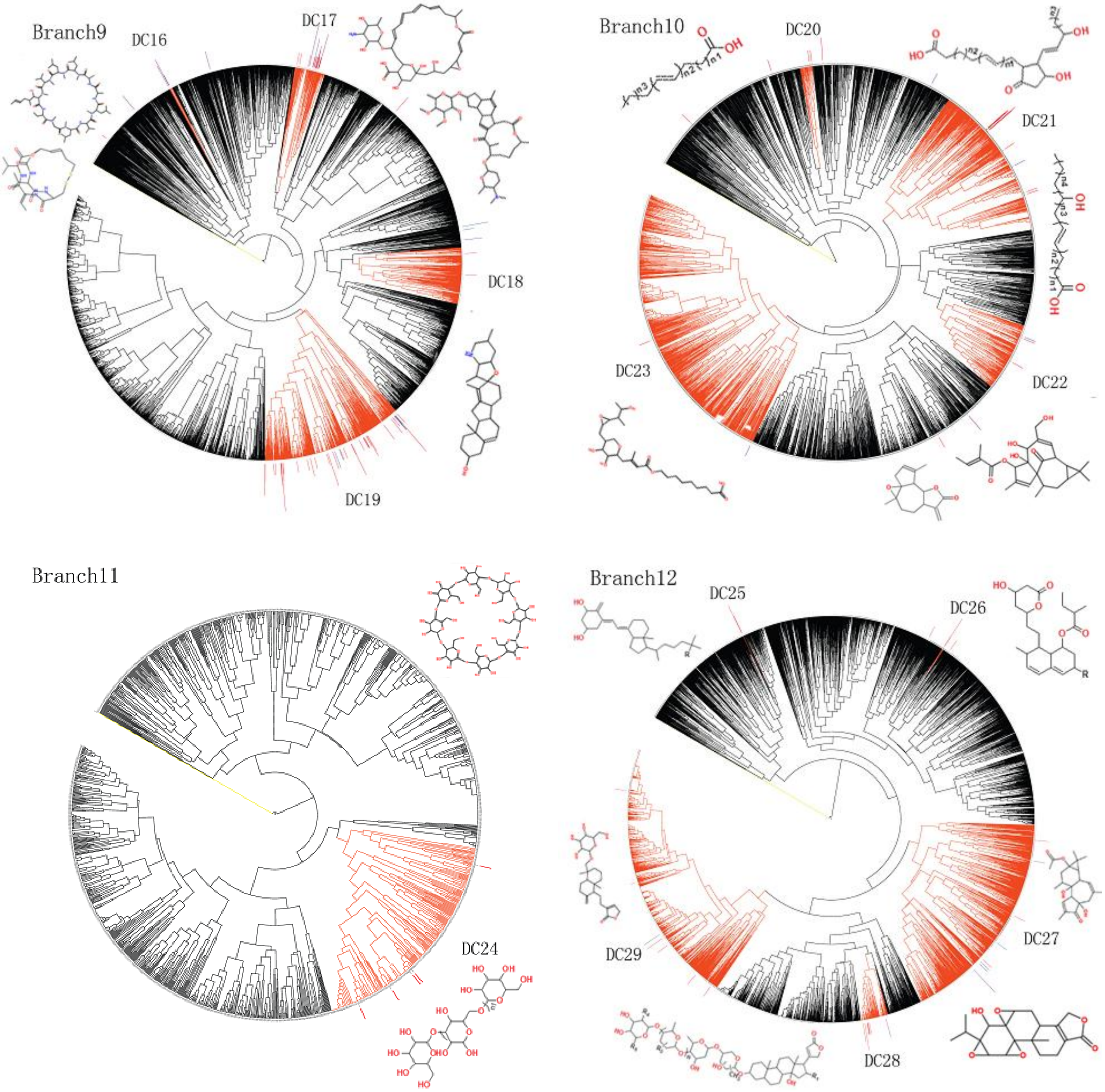

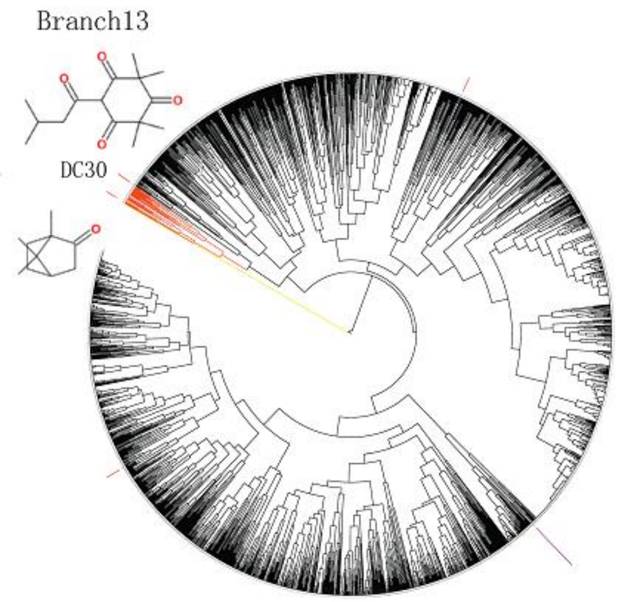
Branch13
DC30

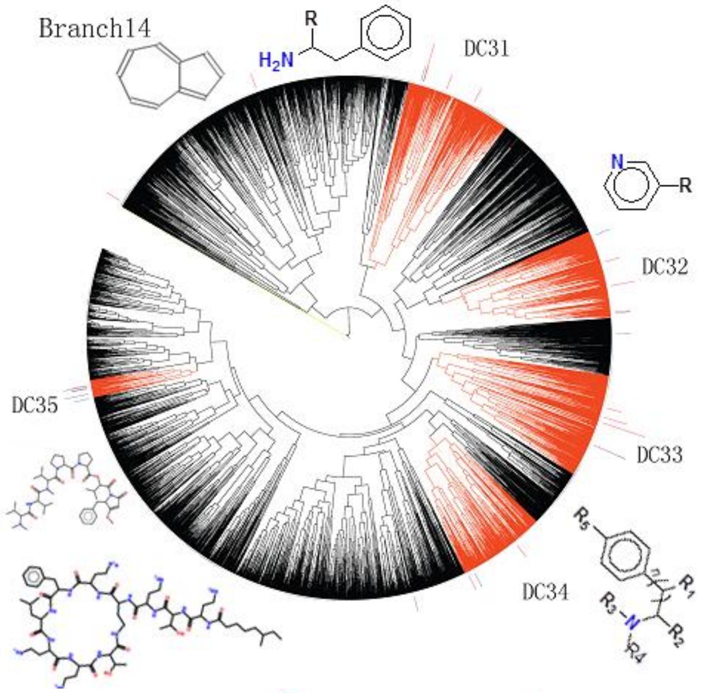
Branch14
DC31
DC32
DC33
DC34
DC35

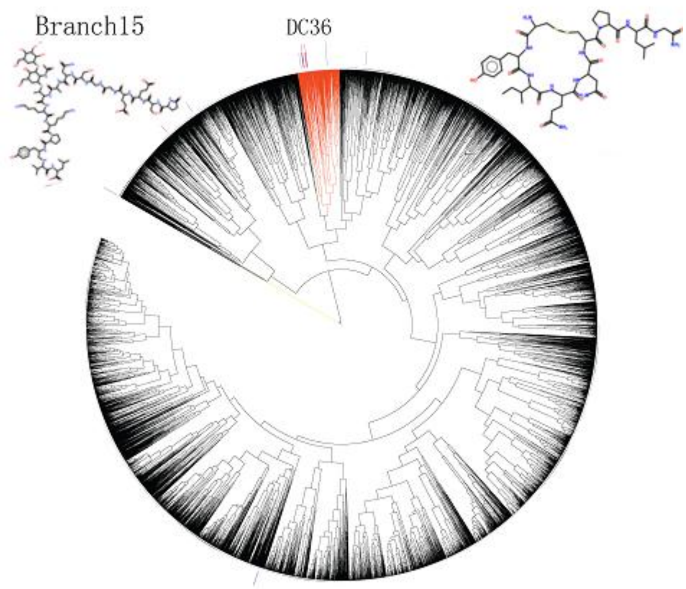
Branch15
DC36

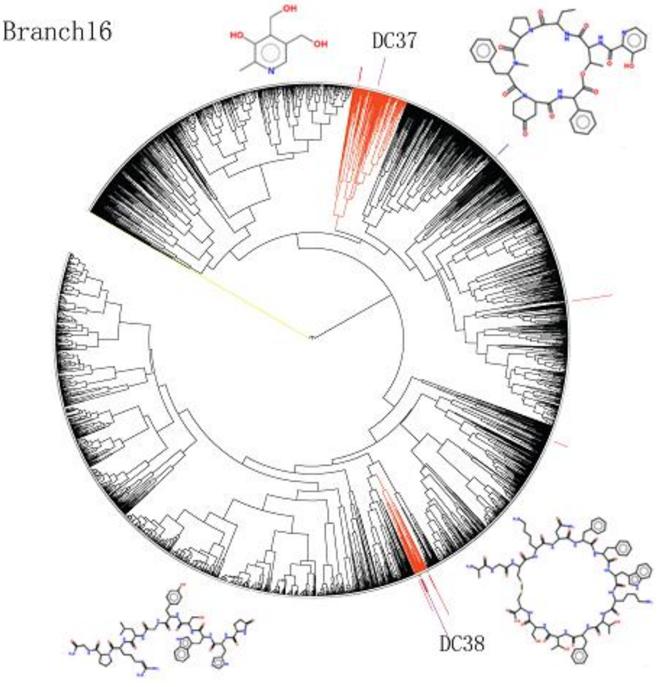
Branch16
DC37
DC38

Supplementary Figure S9 Distribution of the natural product leads of approved and clinical trial drugs in branches 17-24 of the molecular-fingerprint Tanimoto-coefficient similarity clustering tree of the 137,836 natural products and 442 natural product leads. The coloring and labeling schemes are the same as Supplementary Figure S7.

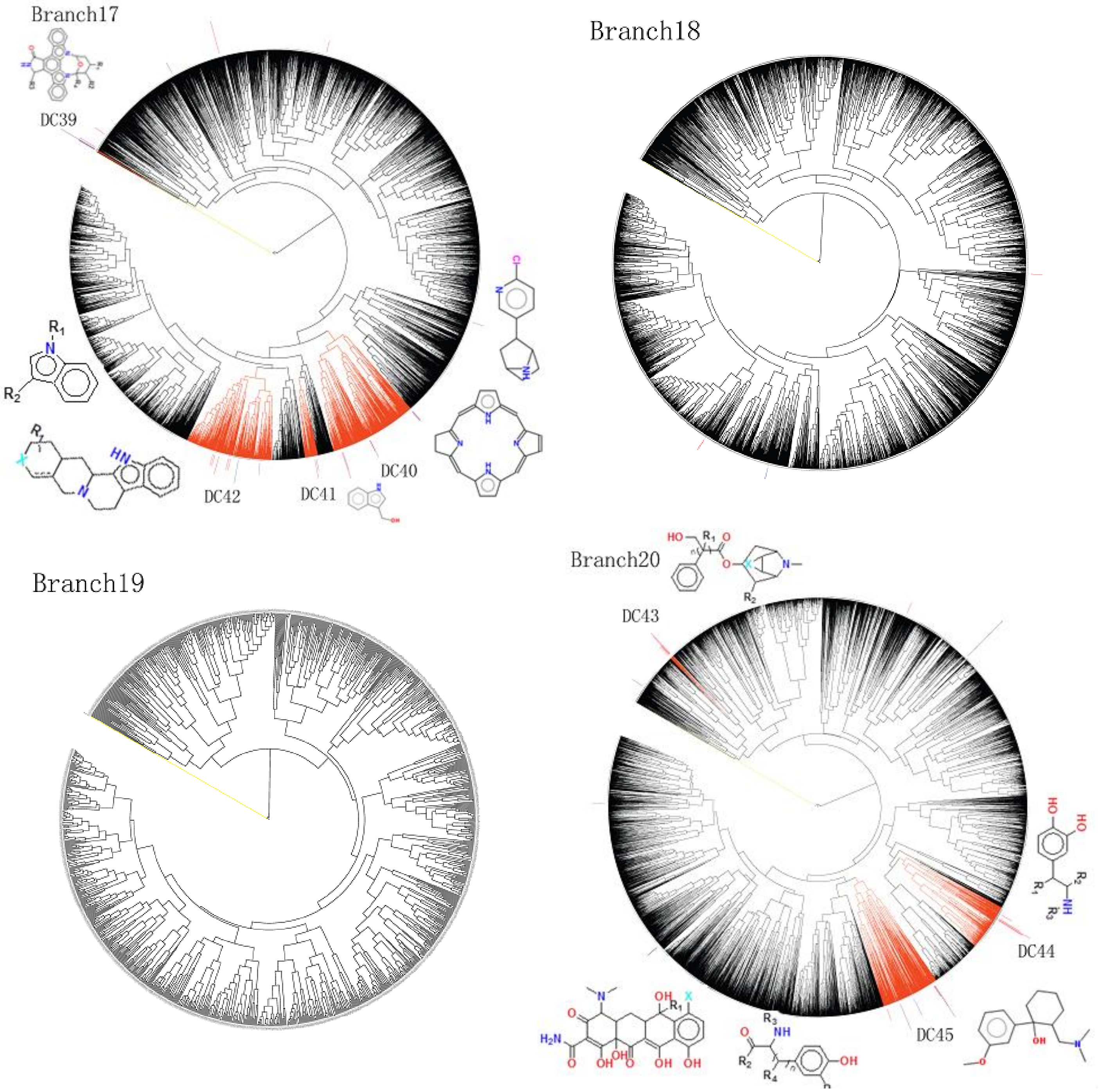

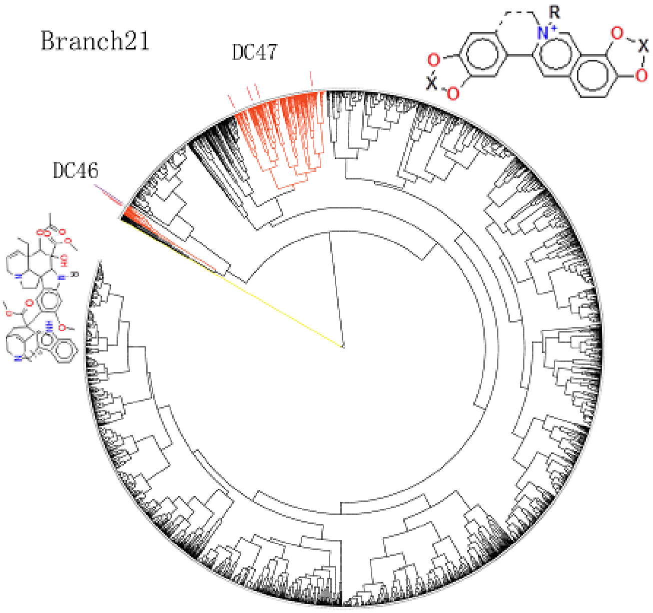
Branch21
DC47
DC46

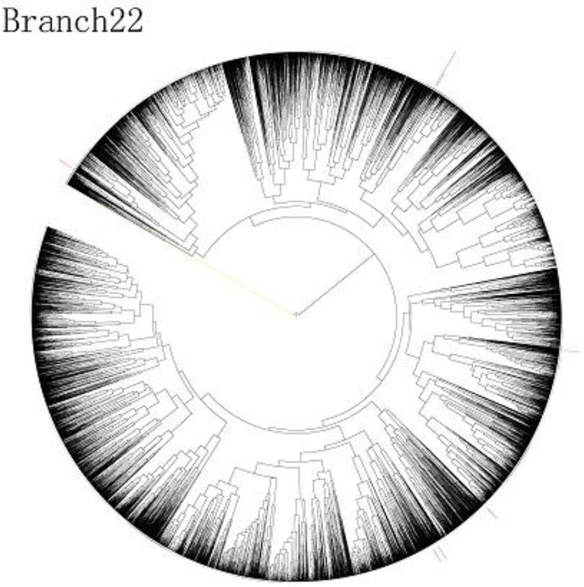
Branch22

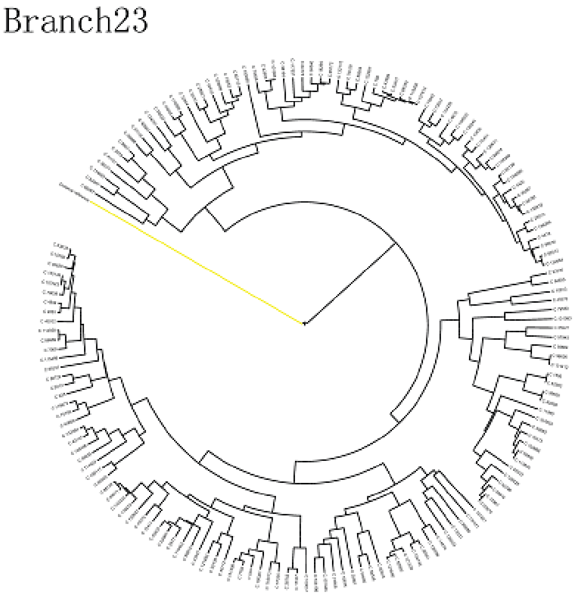
Branch23

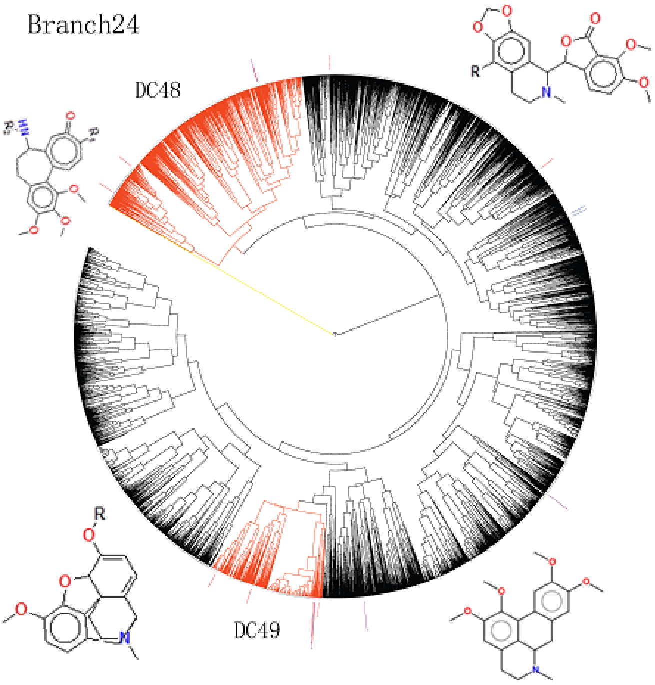
Branch24
DC48
DC49

Supplementary Figure S10 Distribution of the natural product leads of approved and clinical trial drugs in branches 25-33 of the molecular-fingerprint Tanimoto-coefficient similarity clustering tree of the 137,836 natural products and 442 natural product leads. The coloring and labeling schemes are the same as Supplementary Figure S7.

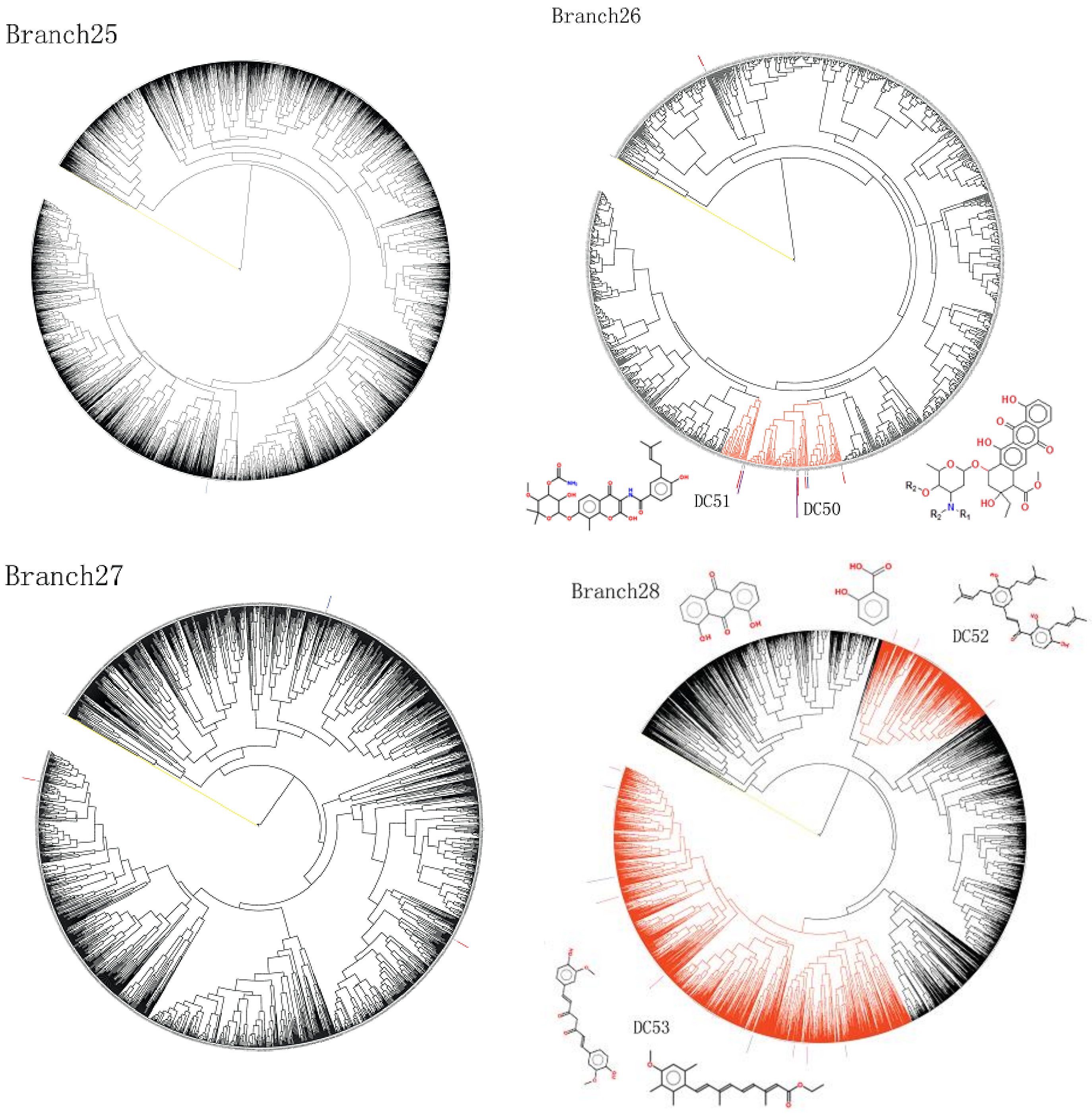

Branch29

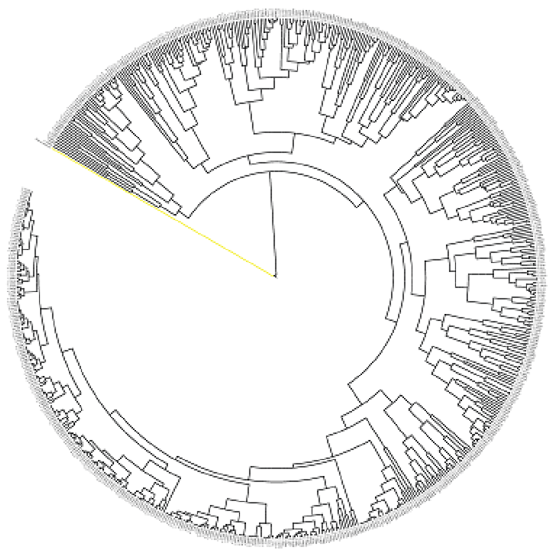

Branch30

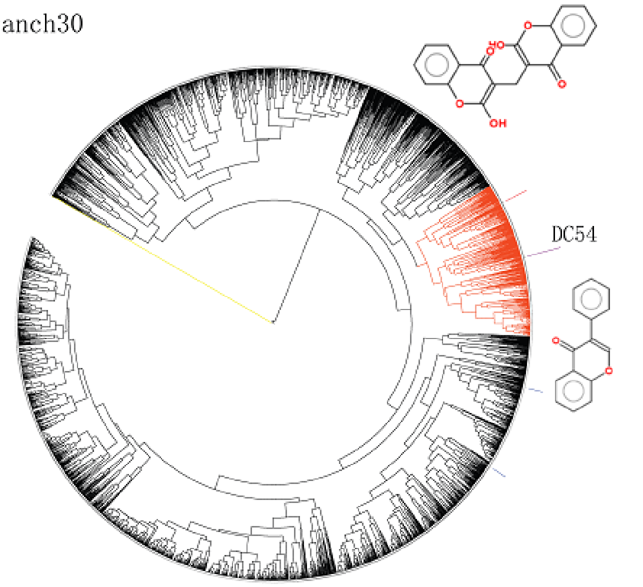

Branch31

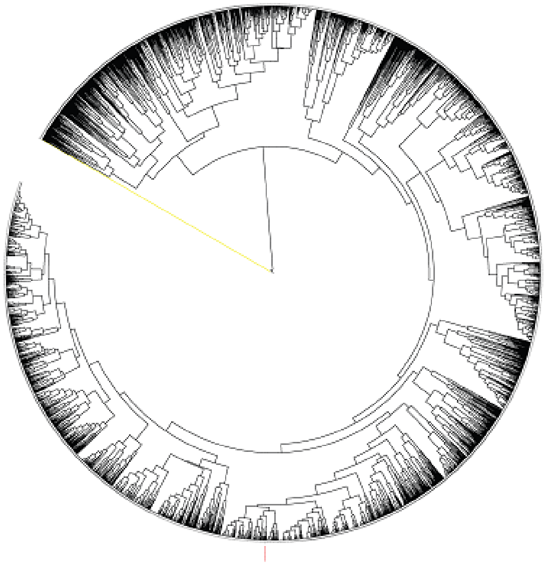

Branch32

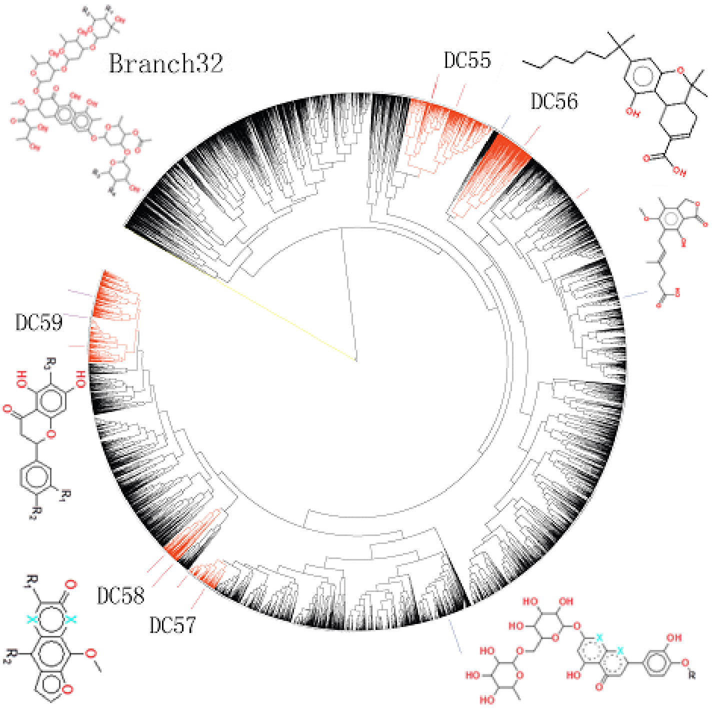

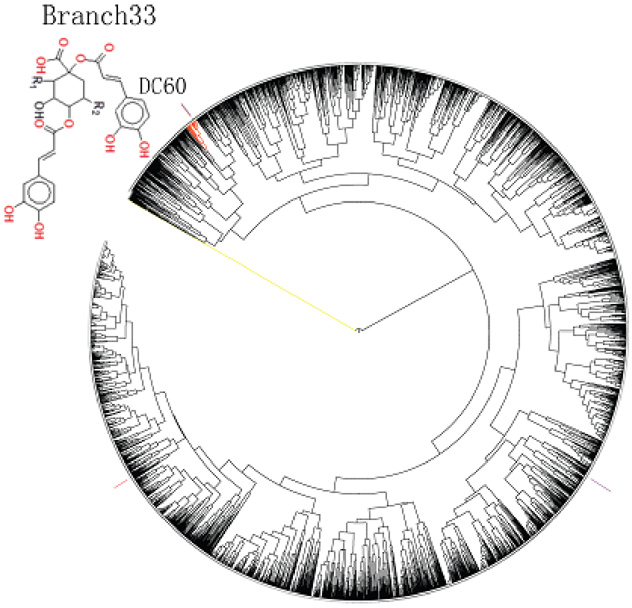
Branch33
DC60

Supplementary Figure S11 The heat map of the proximity matrix of the 442 NPLDs against the NPs in Branch 4. The row and column positions represents the NPLDs and NPs in the same order as their respective positions in the hierarchical clustering tree of the NPLDs and NPs. The column positions of the DCs DC11 (blue), DC12 (green), and DC13 (brown) in this branch are marked on the left side, and the row positions of the NPLDs in DC11 (red), DC12 (green) and DC13 (brown) are marked on the top side of the heat map.

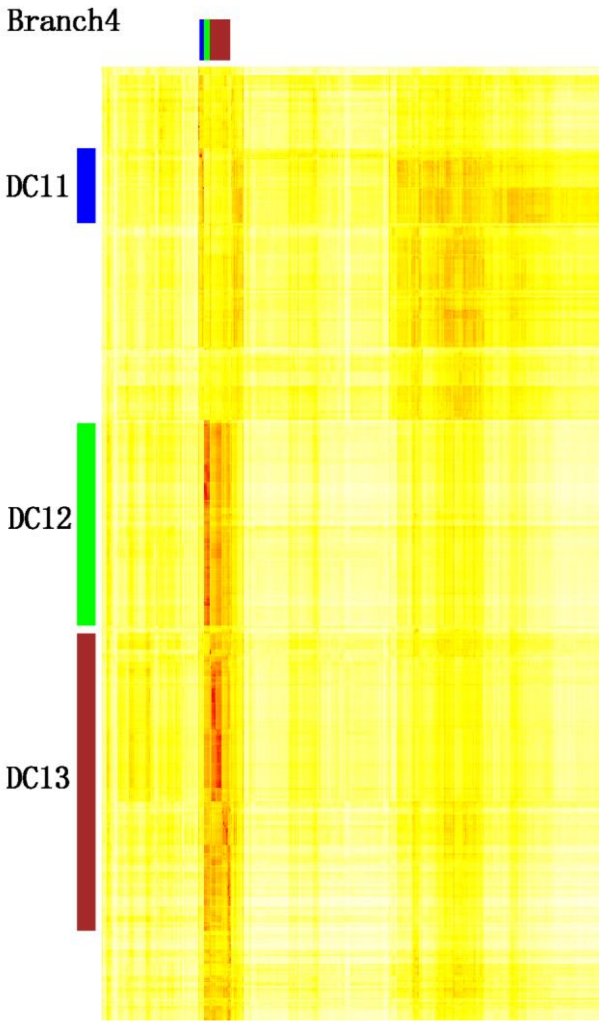

Supplementary Figure S12 The heat map of the proximity matrix of the 442 NPLDs against the NPs in Branch 9. The row and column positions represents the NPLDs and NPs in the same order as their respective positions in the hierarchical clustering tree of the NPLDs and NPs. The column positions of the DCs DC16 (red), DC17 (brown), DC18 (green) and DC19 (blue) in this branch are marked on the left side, and the row positions of the NPLDs in DC16 (red), DC17 (brown), DC18 (green) and DC19 (blue) are marked on the top side of the heat map.

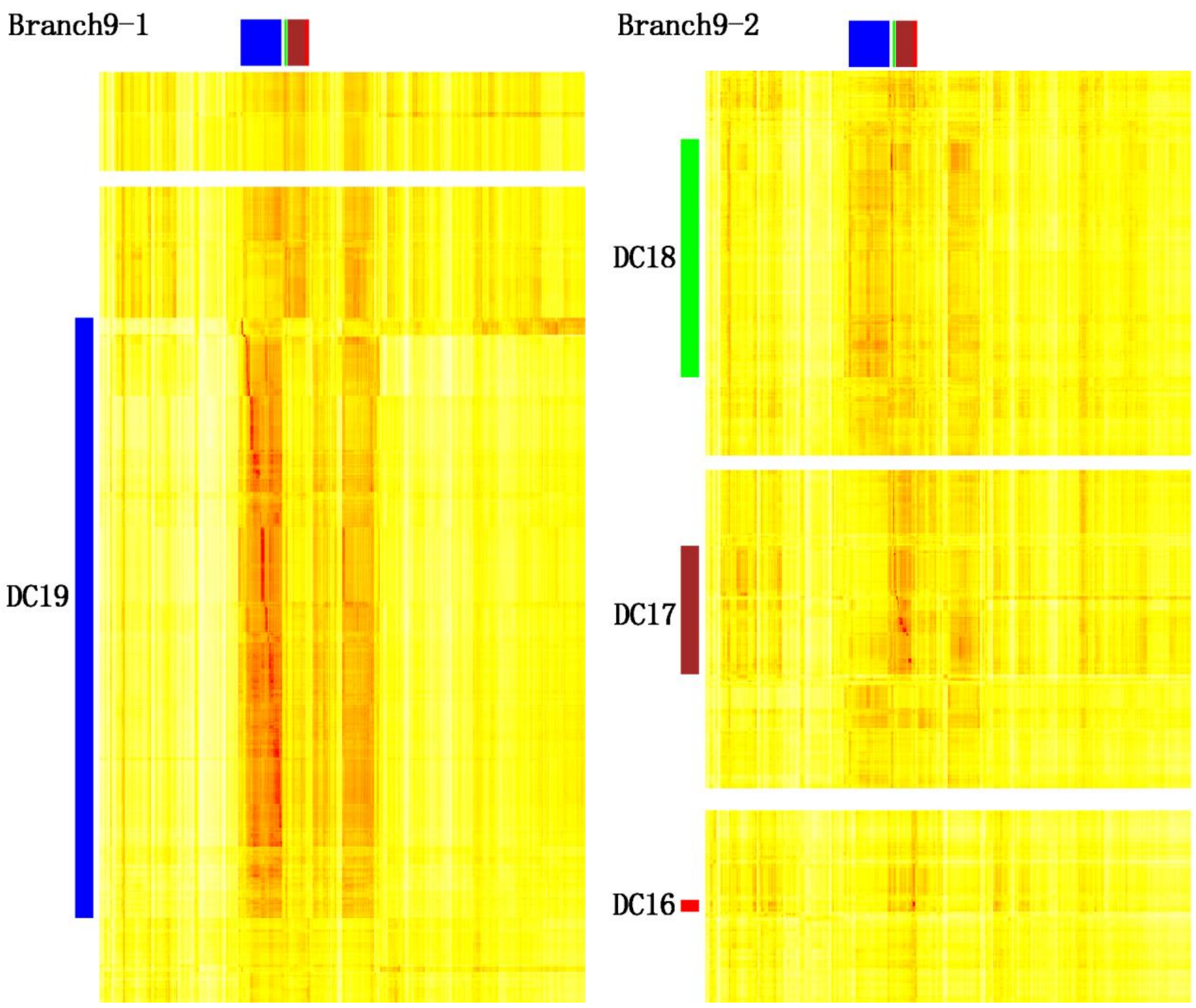

Supplementary Figure S13 The physicochemical landscape of the NPLDs and NPs in branch 9 characterized by the eight physicochemical properties molecular weight, lipophilicity AlogP and logD, polarizability, and the number of O+N, hydrogen bond donor, hydrogen bond acceptor, rotable bonds, and rings. The inner black and red lines represent the NPs outside and inside the DCs in this branch. The lengths of the outer green and non-green (red, purple, blue) lines correspond to the values of the specific physicochemical properties of the NPs and the NPLDs (approved, approved + clinical trial, and clinical trial). The DCs in this branch are marked by their DC ID.

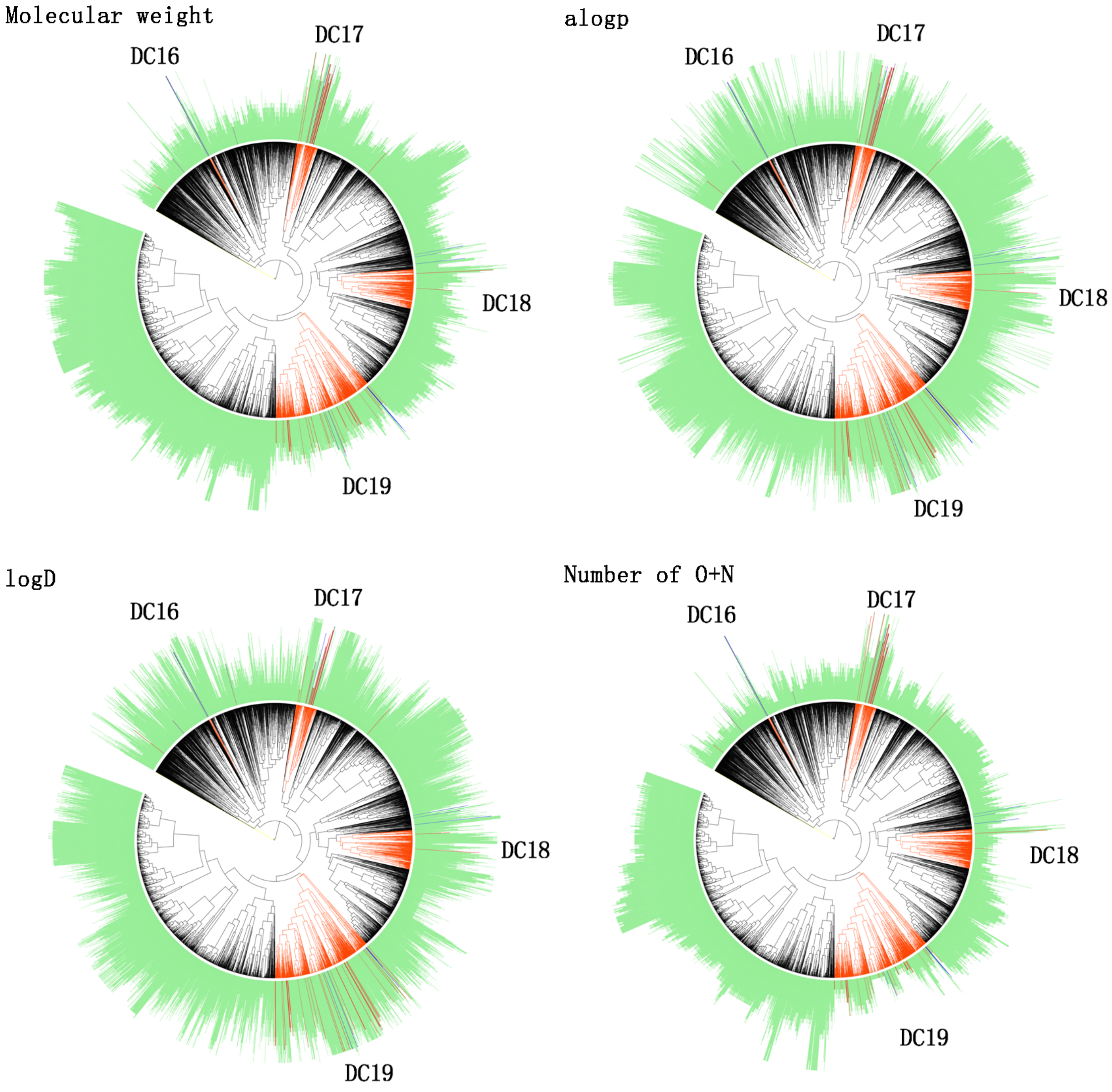

Number of H-bond donnor

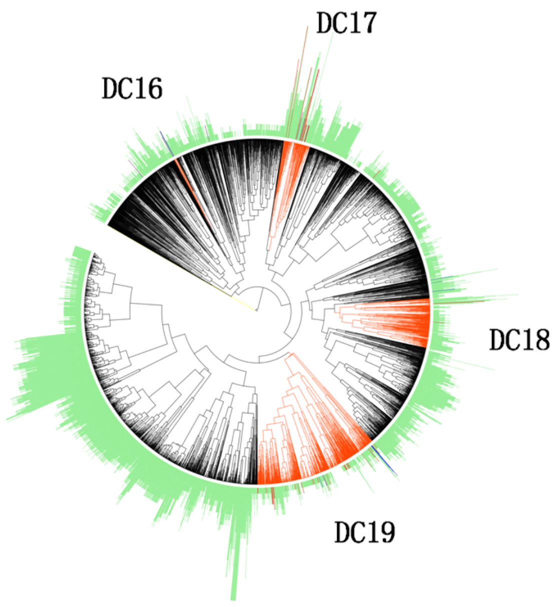

Number of H-bond acceptor

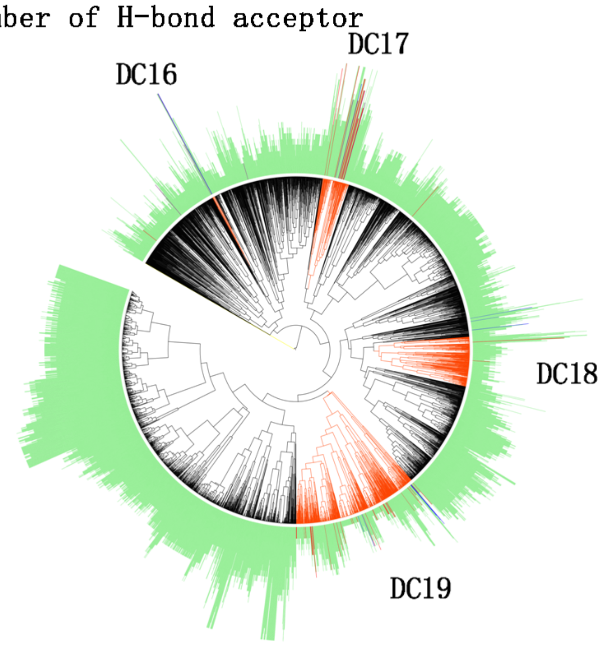

Number of rotable bonds

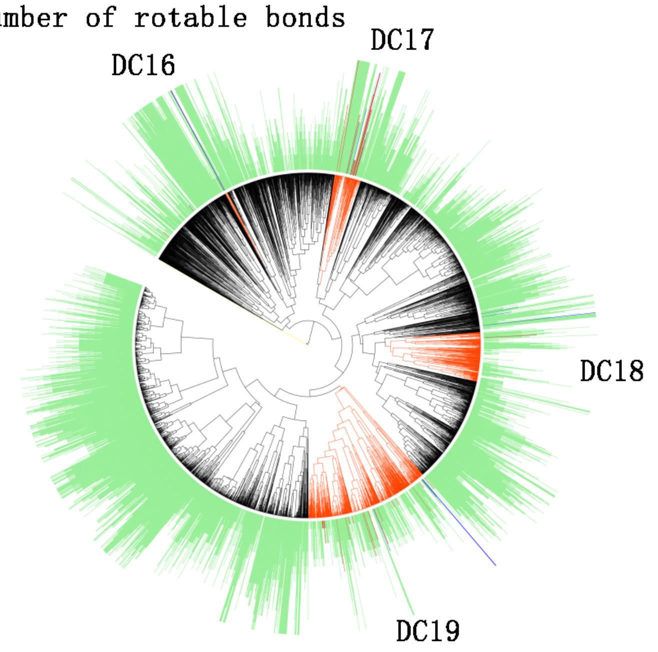

Number of rings

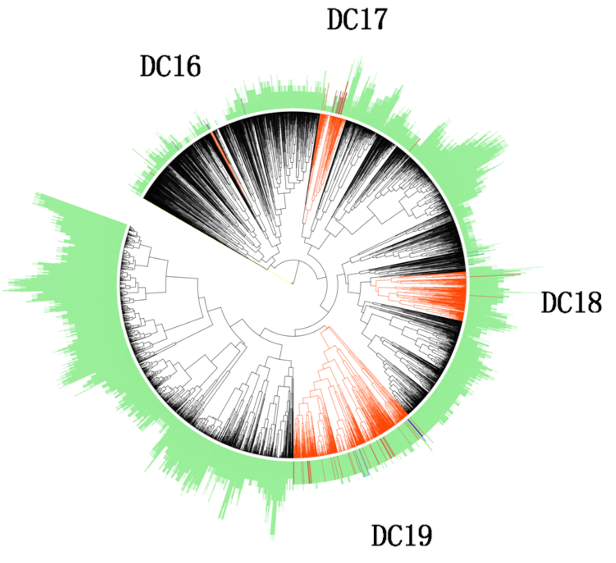

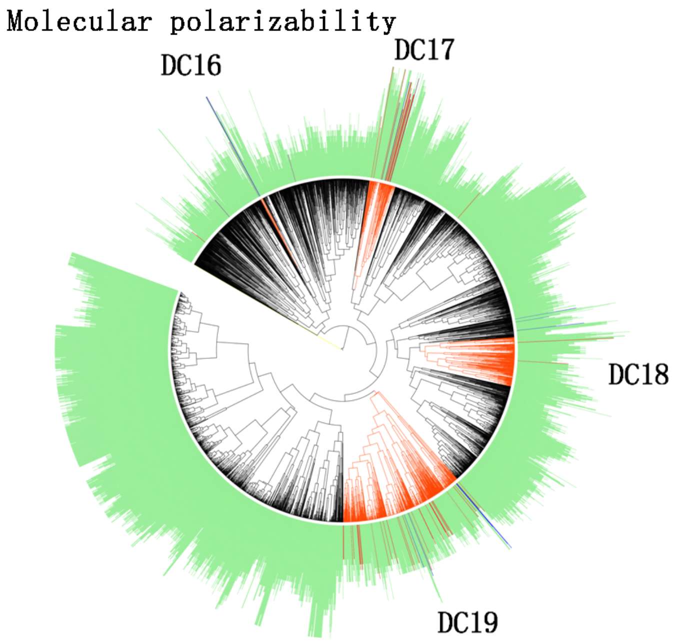
Molecular polarizability
DC16
DC17
DC18
DC19

Supplementary Figure S14 Distribution of the bioactive natural products (green colored lines) with respect to the leads of approved and clinical trial drugs (the red, purple and blue lines on top of the clustering tree) in branches 1-9 of the molecular-fingerprint Tanimoto-coefficient similarity clustering tree of the 137,836 natural products and 442 natural product leads.

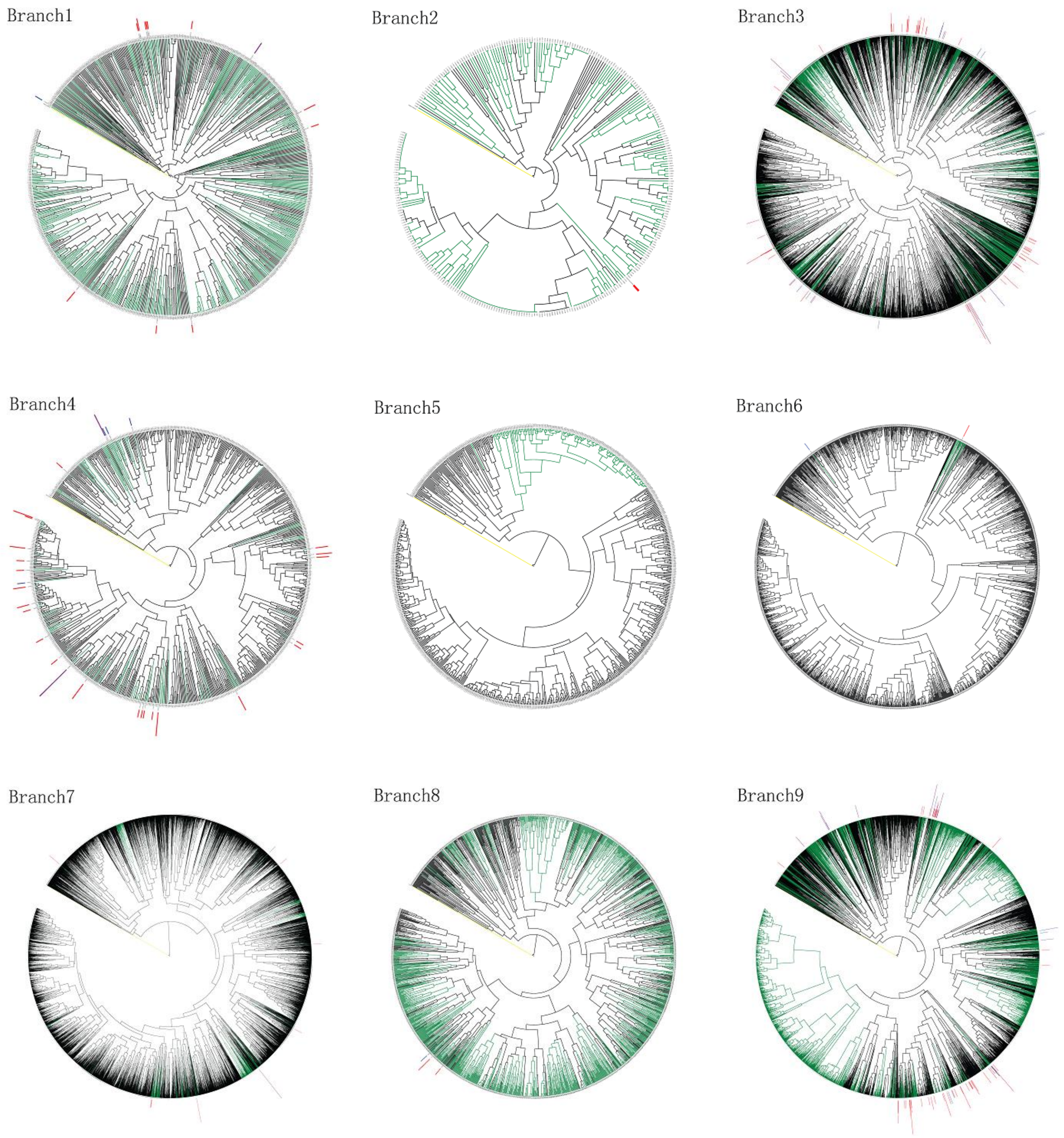

Supplementary Figure S15 Distribution of the bioactive natural products with respect to the leads of approved and clinical trial drugs in branches 10-18 of the molecular-fingerprint Tanimoto-coefficient similarity clustering tree of the 137,836 natural products and 442 natural product leads. The line coloring scheme is the same as Figure S11.

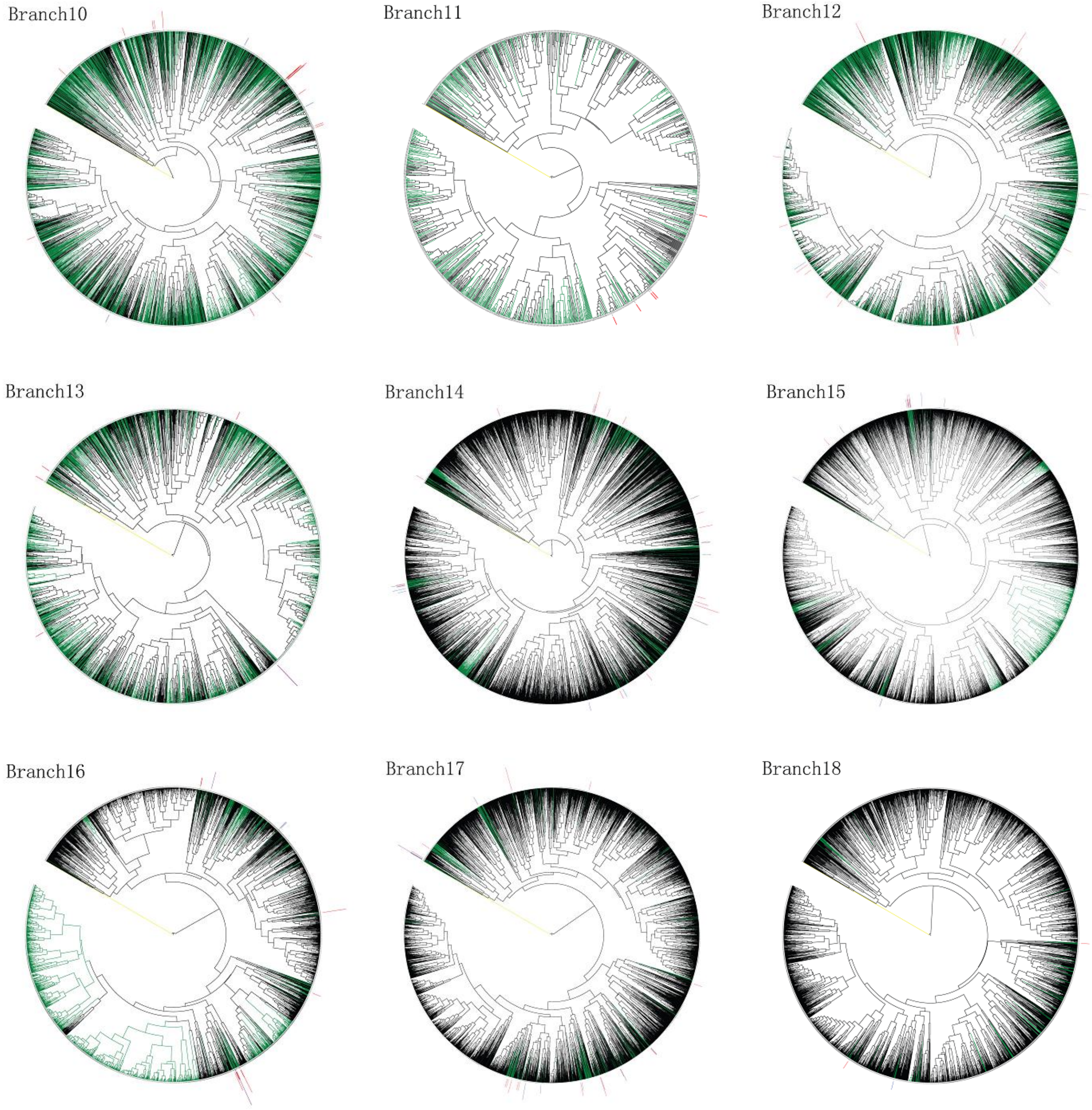

Supplementary Figure S16 Distribution of the bioactive natural products with respect to the leads of approved and clinical trial drugs in branches 19-27 of the molecular-fingerprint Tanimoto-coefficient similarity clustering tree of the 137,836 natural products and 442 natural product leads. The line coloring scheme is the same as Figure S11.

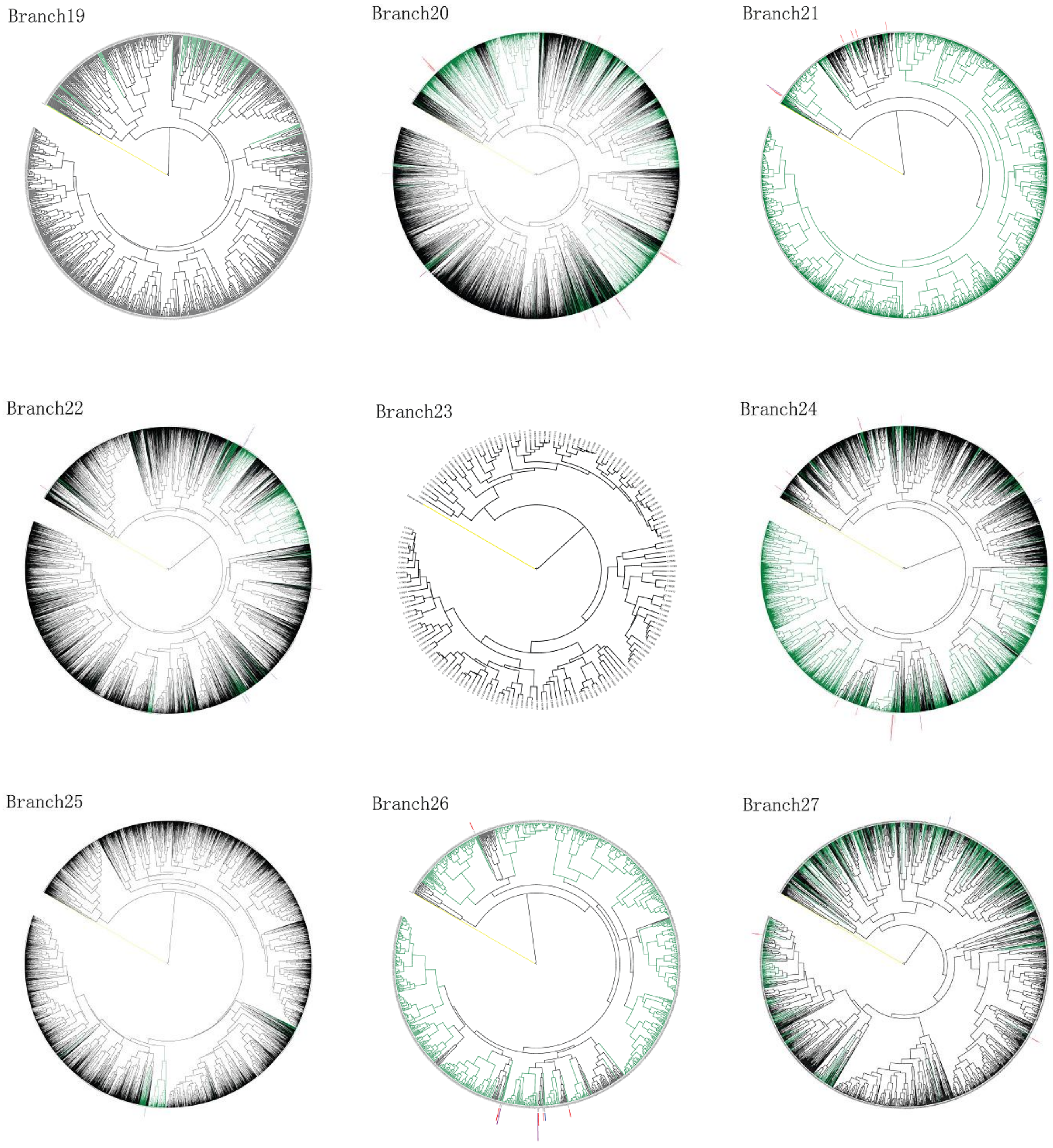

Supplementary Figure S17 Distribution of the bioactive natural products with respect to the leads of approved and clinical trial drugs in branches 28-33 of the molecular-fingerprint Tanimoto-coefficient similarity clustering tree of the 137,836 natural products and 442 natural product leads. The line coloring scheme is the same as Figure S11.

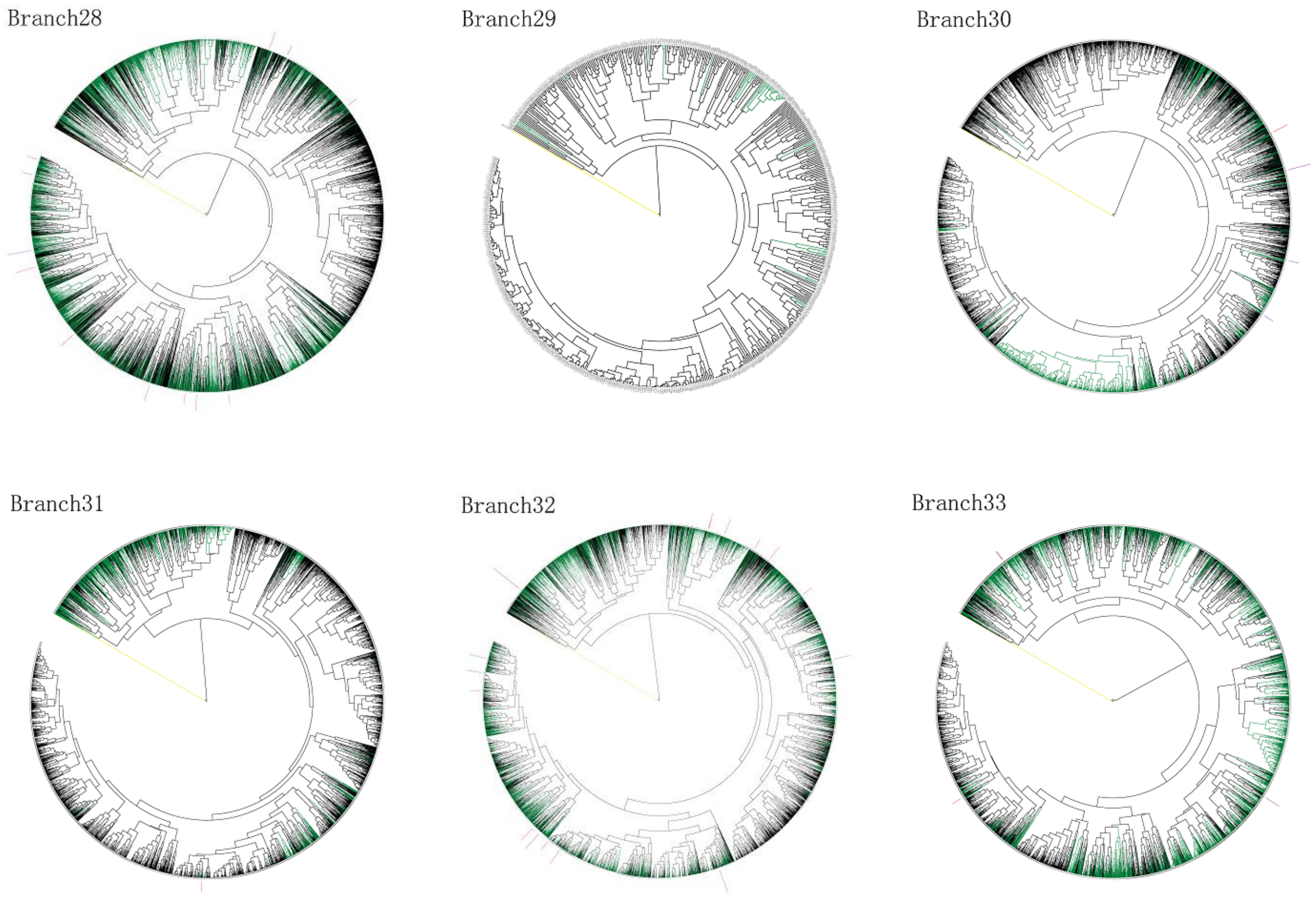

Supplementary Figure S18 The exploration times of the bioactive natural products (green colored lines on top of the clustering tree) and the leads of approved and clinical trial drugs (the red, purple and blue lines on top of the clustering tree) in branches 1-9 of the molecular-fingerprint Tanimoto-coefficient similarity clustering tree. The length of each line on top of the tree correlates to the exploration time of a natural product or a drug lead with a scale of 1 to 11 corresponding to ≤5, 5-10, … , 45-50, and ≥50 years from 2012. The drug-lead productive clusters are in red-orange color.

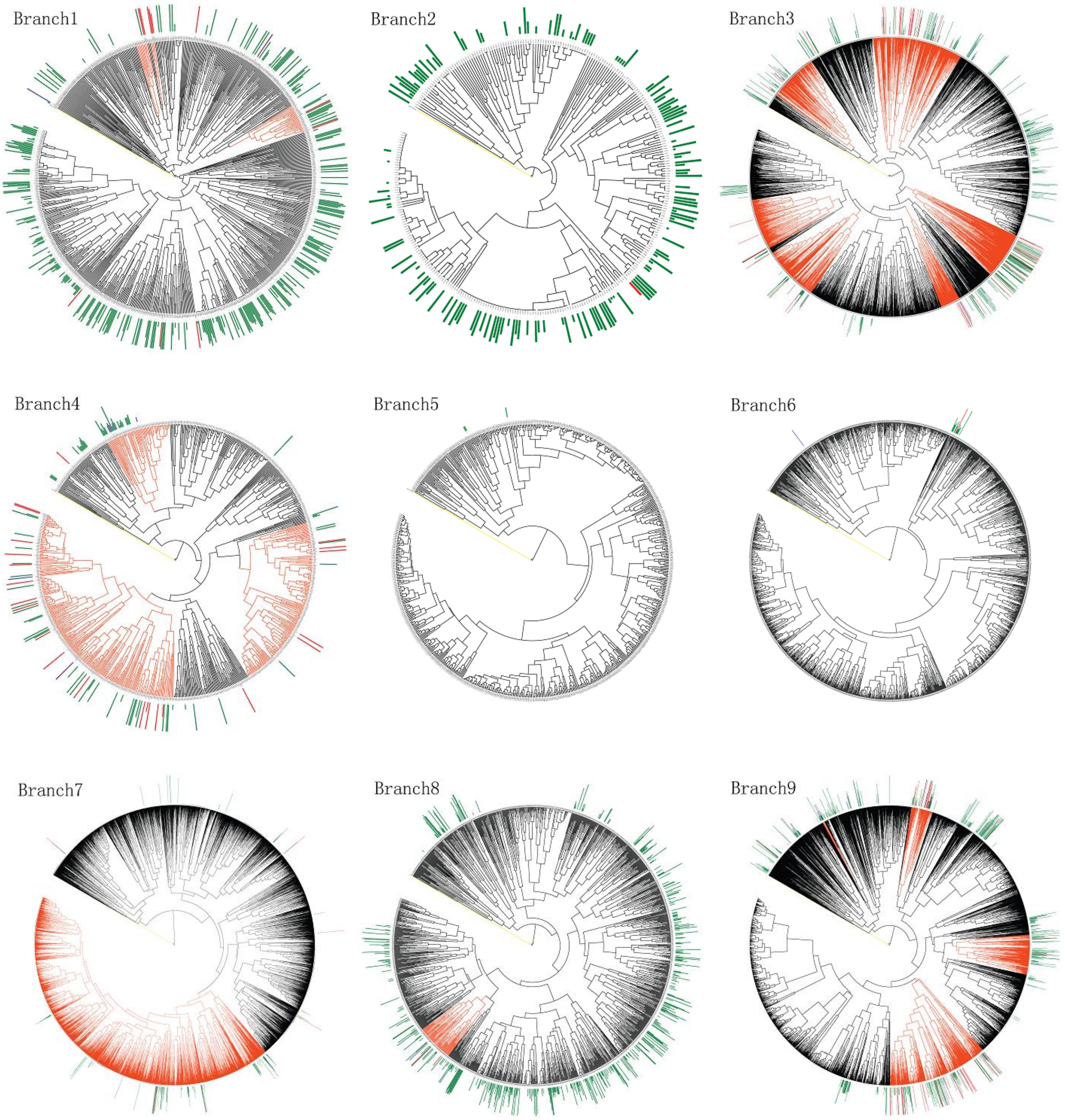

Supplementary Figure S19 The exploration times of the bioactive natural products and the leads of approved and clinical trial drugs in branches 10-18 of the molecular-fingerprint Tanimoto-coefficient similarity clustering tree. The line coloring scheme is the same as in Supplementary Figure S15.

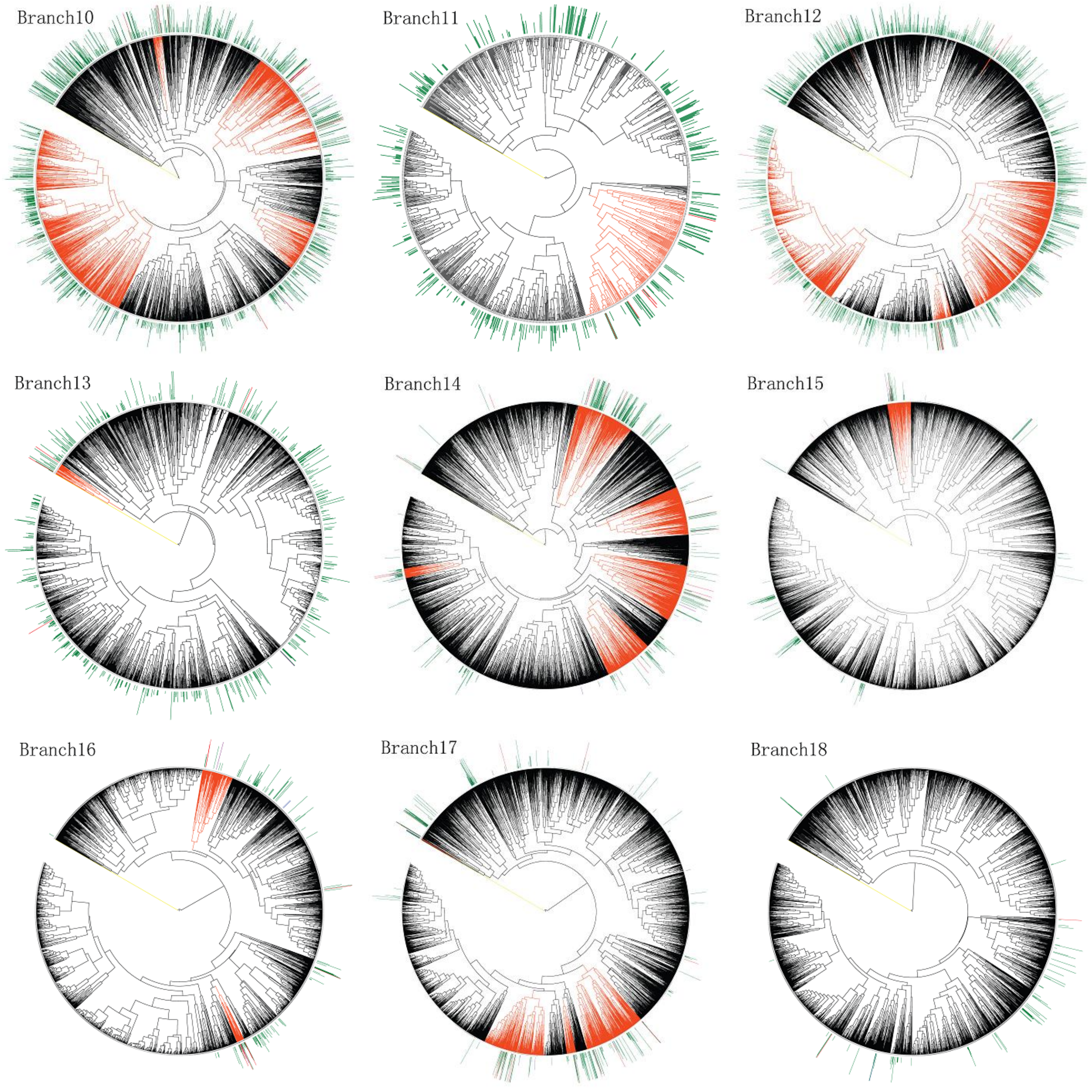

Supplementary Figure S20 The exploration times of the bioactive natural products and the leads of approved and clinical trial drugs in branches 19-27 of the molecular-fingerprint Tanimoto-coefficient similarity clustering tree. The line coloring scheme is the same as in Supplementary Figure S15.

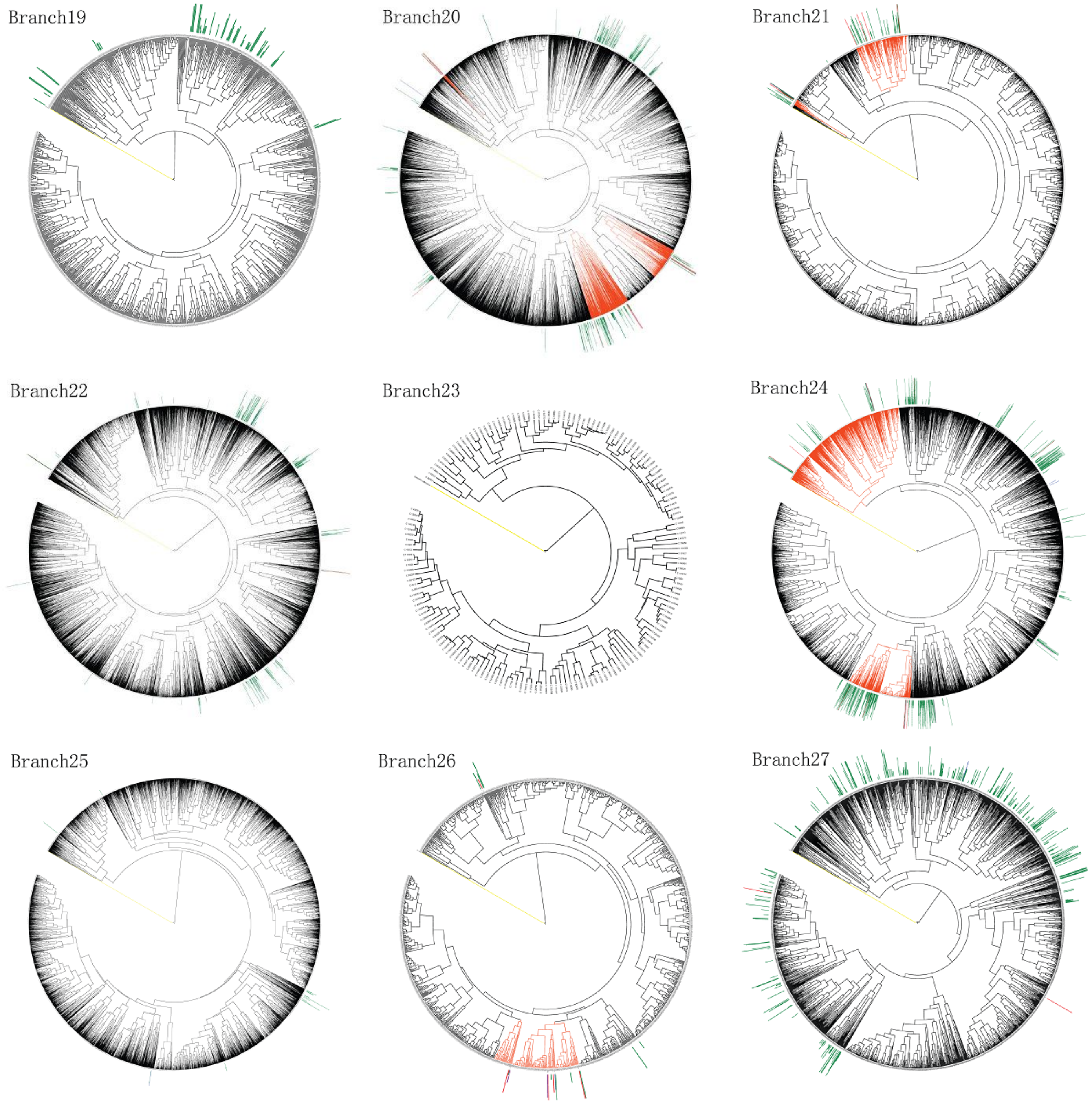

Supplementary Figure S21 The exploration times of the bioactive natural products and the leads of approved and clinical trial drugs in branches 28-33 of the molecular-fingerprint Tanimoto-coefficient similarity clustering tree. The line coloring scheme is the same as in Supplementary Figure S15.

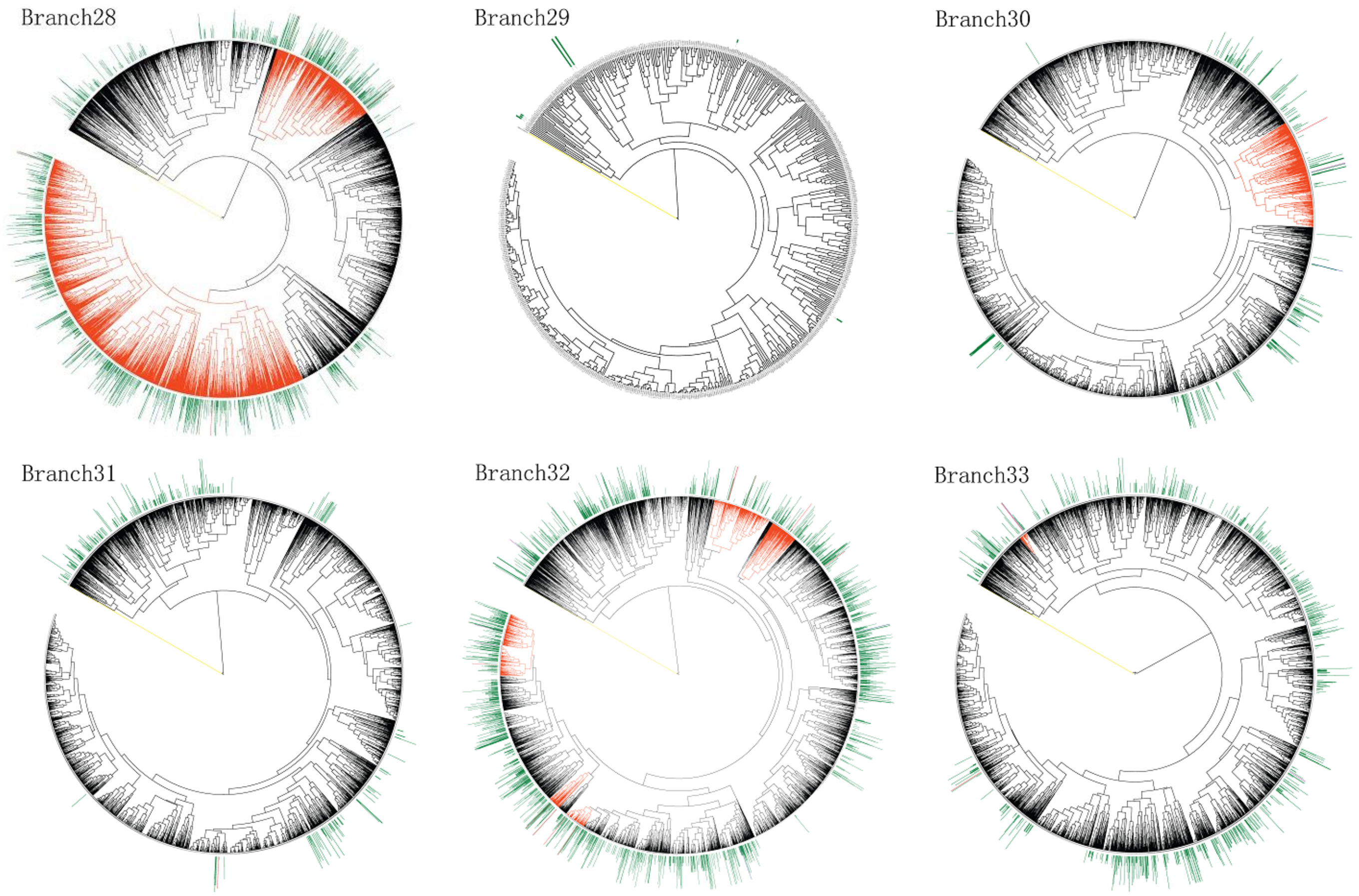

Supplementary Figure S22 Distribution of the approved NP-related drugs, grouped into specific disease classes, in the drug-productive clusters DC1 to DC60. Disease classes are labeled according to the international classification of diseases ICD-10 as: A00-B99 infectious & parasitic diseases (Red), C00-D49 neoplasms (Blue), D50-D89 blood & immune-related diseases, E00-E90 endocrine & metabolic diseases (Purple), F01-F99 mental disorders, G00-G99 nervous system diseases, H00-H59 eye diseases, I00-I99 circulatory system disorders (Green), J00-J99 respiratory system disorders, K00-K95 digestive system disorders, L00-L99 skin diseases, M00-M99 musculoskeletal system & connective tissue disorders, N00-N99 genitourinary system disorders (Orange), O00-O9A reproductive system disorders, P00-P96 perinatal originated diseases, R00-R99 unclassified disorders, S00-T88 injury & poisoning.

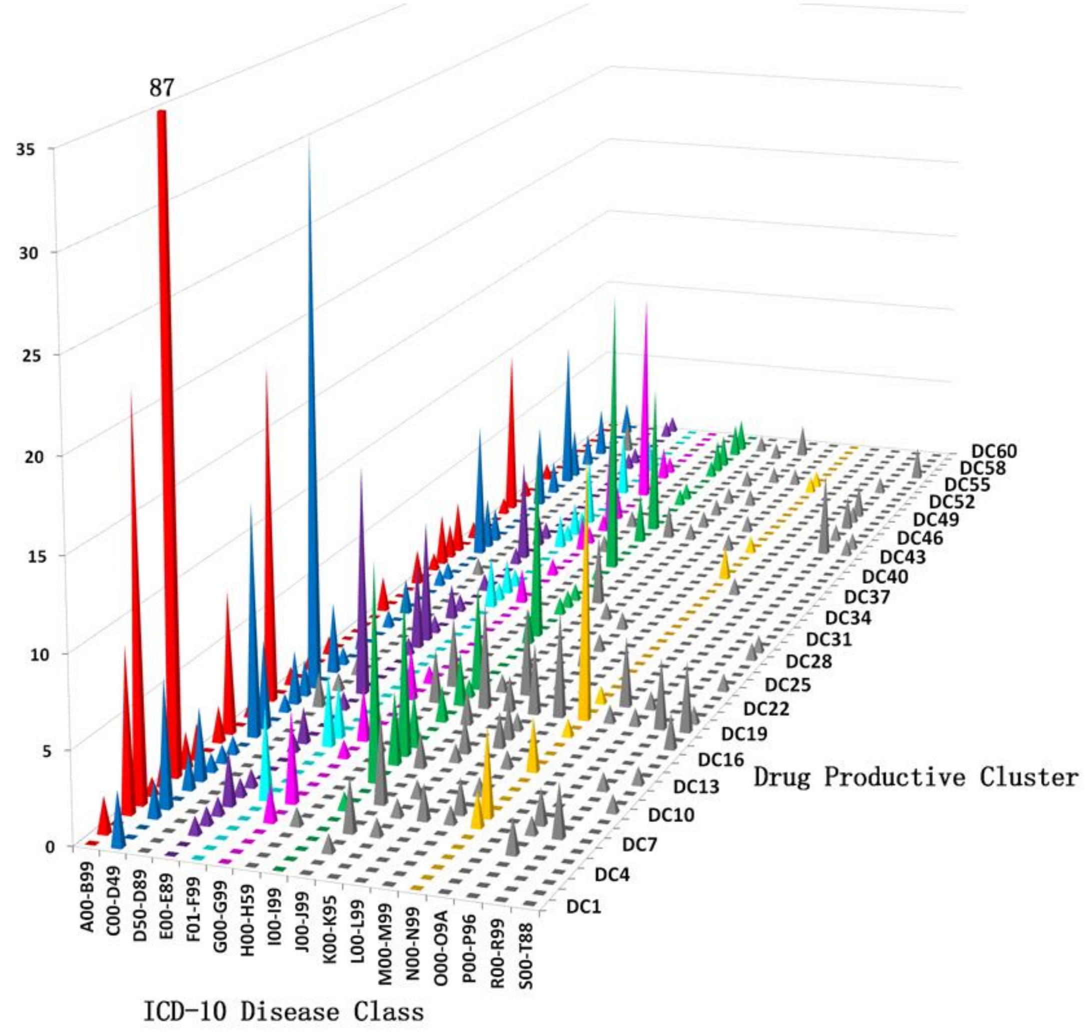

Supplement: Supplementary Information [file srep09325-s1.pdf]
